# Supplementary material for: Impact of population pressure on forest resources depletion in Yayo coffee forest Biosphere Reserve, Southwest Ethiopia
Source: PLoS One. 2026 Jan 5;21(1):e0324407. doi: 10.1371/journal.pone.0324407 (PMC12768366; doi:10.1371/journal.pone.0324407)

Figure S1: Satellite Images Results of Land Use Land Cover data in table 3, Yayo coffee forest Biosphere Reserve from 1984 to 2024, Southwest Ethiopia


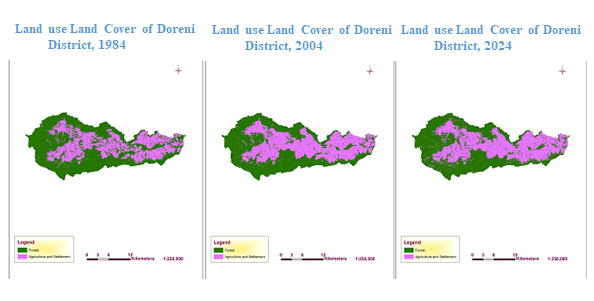

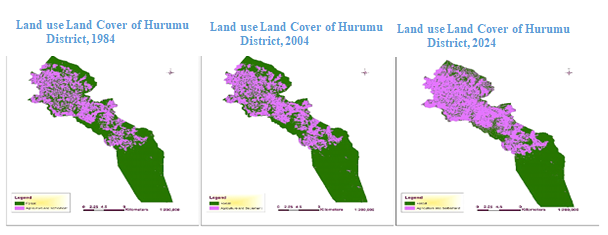

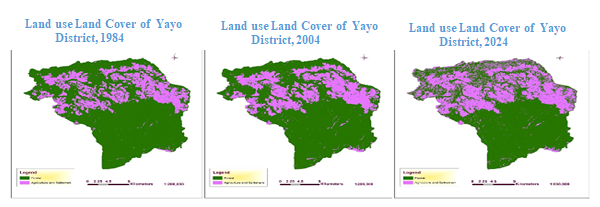

Supplement: S1 Fig — (DOCX) [file pone.0324407.s001.docx]
