# Supplementary material for: Impact of population pressure on forest resources depletion in Yayo coffee forest Biosphere Reserve, Southwest Ethiopia
Source: PLoS One. 2026 Jan 5;21(1):e0324407. doi: 10.1371/journal.pone.0324407 (PMC12768366; doi:10.1371/journal.pone.0324407)
Supplement: S1 Table — (DOCX) [file pone.0324407.s002.docx]

Table S1: Pearson Correlation analysis Data: Population growth Vs Forest cover change, Yayo coffee forest Biosphere Reserve from 1984 to 2024, Southwest Ethiopia

| **Districts** | **Land use land cover** | **1984** | **2004** | **2024** |
| --- | --- | --- | --- | --- |
|  |  | Area (Ha) | Area (Ha) | Area (Ha) |
| Doreni | Forest | 29030.1 | 25395.59 | 24889.52 |
|  | Non-Forest land | 16976.4 | 20610.91 | 21116.98 |
|  | Total | 46006.5 | 46006.5 | 46006.5 |
| Hurumu | Forest | 30848.4 | 29638.65 | 24241.35 |
|  | Non-Forest | 15680.1 | 16889.85 | 22287.15 |
|  | Total | 46528.5 | 46528.5 | 46528.5 |
| Yayo | Forest land | 60208.67 | 57703.34 | 51642.06 |
|  | Non-Forest land | 20608.34 | 23113.66 | 29174.94 |
|  | Total | 80817 | 80817 | 80817 |
| Grand total Forest land | | 120087.17 | 112737.58 | 100772.93 |
