# Supplementary material for: Impact of population pressure on forest resources depletion in Yayo coffee forest Biosphere Reserve, Southwest Ethiopia
Source: PLoS One. 2026 Jan 5;21(1):e0324407. doi: 10.1371/journal.pone.0324407 (PMC12768366; doi:10.1371/journal.pone.0324407)
Supplement: S2 Table — (DOCX) [file pone.0324407.s003.docx]

Table S2: Socio-Economic Determinants Data: Forest Dependency and Conservation Behavior in Yayo coffee forest Biosphere Reserve, Southwest Ethiopia, 2024

| **Participant ID** | **Family Size** | **Income Level (ETB)** | **Education Status (Years)** | **Linear Regression – Dependency on Forest Resources** | **Logistic Regression – Conservation Behavior** |
| --- | --- | --- | --- | --- | --- |
|  |  |  |  | **p-value** | **p-value** |
| 1 | ≤ 3 | 900 | 0 | 0.00 | ≤ 0.05 |
| 2 | 4–5 | 1500 | 1-5 |  |  |
| 3 | 6 –7 | 3000 | 6-12 |  |  |
| 4 | > 7 | 4500 | ≥ Diploma |  |  |
