# Supplementary material for: Impact of population pressure on forest resources depletion in Yayo coffee forest Biosphere Reserve, Southwest Ethiopia
Source: PLoS One. 2026 Jan 5;21(1):e0324407. doi: 10.1371/journal.pone.0324407 (PMC12768366; doi:10.1371/journal.pone.0324407)
Supplement: Attachment — Submitted filename: PONE-D-25-21231_R1.pdf [file pone.0324407.s006.pdf]

PLOS ONE

IMPACT OF POPULATION PRESSURE ON FOREST RESOURCES DEPLETION IN YAYO COFFEE FOREST BIOSPHERE RESERVE, SOUTHWEST ETHIOPIA

--Manuscript Draft--

**Manuscript Number:**	PONE-D-25-21231R1
**Article Type:**	Research Article
**Full Title:**	IMPACT OF POPULATION PRESSURE ON FOREST RESOURCES DEPLETION IN YAYO COFFEE FOREST BIOSPHERE RESERVE, SOUTHWEST ETHIOPIA
**Short Title:**	This study investigates how increasing population pressure contributes to the decline of forest resources
**Corresponding Author:**	Fikru Mosisa Hunde, MSc<br>Mettu University: Mattu University<br>Mattu, ETHIOPIA
**Keywords:**	Biosphere reserve, degradation, forests, population pressure, land use
**Abstract:**	The research was carried out in the Yayo Coffee Forest Biosphere Reserve, located in the Oromia region of southwest Ethiopia. Data were gathered from key informants, kebele leaders, development agents, selected households, focus group discussions, and field observations. Land use and land cover change data were obtained from satellite imagery spanning 1984 to 2007. Population growth data were sourced from the Ethiopian Central Statistical Agency's Jimma branch. Additionally, field visits included direct observations of the forest's condition. A total of 69 participants responded to both structured and semi-structured questionnaires designed to address the study's objectives. The communities in the study area do not rely on a single livelihood strategy but instead engage in various activities such as crop cultivation, forestry, livestock rearing, and off-farm work, with varying levels of dependence on each. Among the forest products collected, coffee, firewood, honey, and wild spices were the most common. The main factors contributing to forest degradation were ranked as population growth (88.6%), illiteracy (75.7%), lack of local participation (73.9%), poverty (64.3%), investment (47.1%), and urbanization (42.9%), in decreasing order. Satellite image analysis revealed a negative correlation between population growth and forest cover, with a significant difference at P<0.05. The study highlights the impact of population pressure on forest resource degradation in the Yayo Coffee Forest Biosphere Reserve, stressing the need for effective strategies focused on sustainable management, conservation, and utilization of the biosphere reserve.
**Order of Authors:**	Fikru Mosisa Hunde, MSc
	Adanech Asfaw Benti, MSc
	Tefera Jogora Kepula, PhD
**Response to Reviewers:**	Response to Reviewer Comments<br>Manuscript Title: Impact of Population Pressure on Forest Resources Depletion in Yayo Coffee Forest Biosphere Reserve, Ilubabor Zone, Oromia Regional State, Southwest Ethiopia<br>Manuscript ID: PONE-D-25-21231<br>Dear PLOS ONE Editorial Team,<br>We sincerely thank you for the opportunity to revise our manuscript and express our appreciation to the reviewers for their insightful comments and suggestions that have helped us to improve the quality and clarity of our paper.<br>We have carefully considered all the reviewer's comments and revised the manuscript accordingly. Below we provide a detailed, point-by-point response to the reviewer's remarks. For clarity, we have included each comment in bold followed by our responses in regular text. All changes made in the manuscript are highlighted for your convenience.<br>Reviewer #1:<br>Comment #1. Dear authors your study topic is interesting but it needs major revisions, especial grammar and lack of citations are the major problems of the study, please solve this problem and related ones. Also make the mape legevisible for readers!

Powered by Editorial Manager® and ProduXion Manager® from Aries Systems Corporation

	Response #1. We sincerely thank the reviewer for the constructive feedback and appreciation of the study's topic. In response:<br>We have thoroughly revised the manuscript for grammar, clarity, and academic writing quality. To ensure correctness, we conducted a full language edit and proofreading.<br>We have reviewed the manuscript and added relevant and up-to-date citations to strengthen the literature support and properly credit prior research.<br>The presentation of the Mean Absolute Percentage Error (MAPE) has been improved. Specifically, we have clarified its definition, usage, and interpretation in the methodology and results sections. We also ensured it is clearly labeled and easy to interpret in the tables and figures.<br>Reviewer #2:<br>Comment #1.<br>1.Nobility of the Study: Critical Review of the Manuscript<br>Strengths: The manuscript focuses on a critical environmental issue in a globally significant biosphere reserve. It addresses the pressing need for sustainable forest management amidst growing population pressures.<br>Weaknesses: The manuscript does not highlight the global implications of its findings. For instance, the impact of forest loss in the Yayo Coffee Forest Biosphere Reserve on carbon sequestration and global biodiversity hotspots could be better contextualized.<br>Recommendation: Explicitly link the study's implications to global environmental challenges, such as climate change and biodiversity loss.<br>Response #1.<br>We sincerely thank the reviewer for the thoughtful evaluation and constructive suggestion. In response, we have revised the introduction, discussion and conclusion sections to better highlight the global significance of our findings. Specifically, we now discuss the broader implications of forest loss in the Yayo Coffee Forest Biosphere Reserve in terms of carbon sequestration, contributions to global biodiversity conservation, and relevance to international climate and sustainability goals. These additions help to clearly position our study within the context of global environmental challenges, such as climate change and biodiversity loss.<br>The revisions can be found on page2, paragraph 3; page 16, paragraph 2 and page 19, paragraph 2<br>Comment # 2. Statement of the Problem and Knowledge Gap<br>• Strengths: The problem is articulated in the context of population pressure and forest degradation.<br>• Weaknesses: The knowledge gap lacks specificity. While the manuscript states that population growth affects forest resources, it does not critically assess why current solutions are insufficient or how this study provides a novel perspective.<br>• Recommendation: Expand the introduction to include a critical review of existing solutions and their shortcomings. Clearly articulate how this research addresses those gaps.<br>Response # 2<br>We thank the reviewer for this insightful observation. In response, we have revised the introduction to include a more detailed and critical review of existing interventions aimed at mitigating forest degradation under population pressure. We now discuss the limitations of current policy frameworks, enforcement mechanisms, and community-based management approaches, particularly in the context of the Yayo Coffee Forest Biosphere Reserve. Furthermore, we have clearly articulated how our study offers a novel contribution by integrating socio-ecological analysis with geospatial data to assess the effectiveness of existing measures. These revisions help clarify the specific knowledge gap and the unique perspective our study brings.<br>The revised content can be found on page 3, paragraph 3.<br>Comment #3: Objective<br>• Strengths: Objectives are clear and focused.<br>• Weaknesses: The objectives could be more concise and aligned directly with the problem statement.<br>• Recommendation: Reframe the objectives to reflect measurable outcomes. For example, "Quantify the impact of population growth on forest cover using satellite imagery from 1984 to 2024."<br>Response # 3<br>We appreciate the reviewer's positive feedback and helpful recommendation. In response, we have revised the objectives to make them more concise and explicitly aligned with the problem statement. The updated objectives now clearly reflect measurable outcomes, including the quantification of forest cover change over time

Powered by Editorial Manager® and ProduXion Manager® from Aries Systems Corporation

and the assessment of population growth impacts using geospatial data from 1984 to 2024. This reframing enhances the clarity and focus of the study and better communicates its purpose to the reader.
The revised objectives are presented in the introduction section on page 3 paragraph 4
Comment #4. Method of Data Collection
• Strengths: The mixed-methods approach adds depth and breadth to the analysis.
• Weaknesses: The manuscript fails to justify the selection of methods, particularly the use of satellite imagery spanning specific years and the choice of SPSS for analysis.
• Recommendation: Provide citations for methodological protocols. For instance, why were 1984, 1994, 2004 and 2024 chosen for satellite imagery? What validation steps were taken to ensure accuracy in satellite data interpretation? Include a clear rationale for the statistical tools employed.
Response #4
We thank the reviewer for this valuable feedback and for recognizing the strengths of our mixed-methods approach. In response to the concerns raised:
•We have now provided a detailed justification for the selection of satellite imagery years (1984, 1994, and 2024). These years were chosen based on data availability, major policy or demographic shifts in the region, and their relevance in capturing long-term trends in land cover change. We have added relevant references to support this temporal selection.
•We have also described the image classification and accuracy assessment procedures, including the use of ground truth data, historical Google Earth imagery, and confusion matrices to validate classification results. The methods and validation steps are now clearly stated in the revised methodology section.
•Furthermore, we have included a rationale for using SPSS, explaining its role in analyzing survey data and conducting descriptive and inferential statistical analyses, such as correlation and regression, to examine relationships between population dynamics and forest cover change. Supporting citations have been added where appropriate.
These revisions are included in the methodology section on pages 18 – 21.

Comment # 5: Method of Data Analysis
• Strengths: The use of SPSS and Pearson correlation is appropriate for quantitative data.
• Weaknesses: The analysis lacks depth in exploring causality. For example, while correlation is established, causation is not discussed.
• Recommendation: Include regression analysis or other advanced statistical methods to explore causality. For qualitative data, provide representative quotes or narratives to substantiate thematic findings.
Response #5
We appreciate the reviewer’s thoughtful comments and suggestions to enhance the rigor of our data analysis. In response:
•We have extended our quantitative analysis by incorporating multiple linear regression models to better explore causal relationships between population growth variables and forest cover change. The regression results are now included and interpreted in the revised results section.
•In addition to Pearson correlation, the use of regression analysis enables us to assess the strength and direction of potential causal links while controlling for confounding variables.
•For the qualitative data, we have included representative quotes from key informant interviews and focus group discussions to support and illustrate the main themes identified. These narratives provide greater context and depth to the findings.
All additions and revisions are detailed in the results and conclusion sections on pages 21-22
Comment #6. Results
• Strengths: The results are comprehensive and supported by tables and figures.
• Weaknesses: The presentation is cluttered and lacks coherence. The socio-economic data is presented without linking it to the broader research question.
• Recommendation: Simplify tables and figures, ensuring each directly addresses a research objective. Use visual aids like trend lines or pie charts to make data more digestible.
Response #6

We thank the reviewer for acknowledging the comprehensiveness of our results and for the valuable suggestions to improve clarity and coherence. In response:
•We have simplified and reorganized the tables and figures to ensure each one directly corresponds to a specific research objective or key finding. Unnecessary or redundant visuals have been removed, and labels and captions have been improved for clarity.
•To enhance readability, we introduced more visual aids such as trend lines, pie charts, and bar graphs where appropriate—particularly for time-series and categorical socio-economic data. These revisions help highlight key trends and make the data more accessible to readers.
•Additionally, we revised the narrative surrounding the socio-economic data to better connect the findings with the main research question and theoretical framework. This strengthens the linkage between data and interpretation.
The revised figures and results are presented in the results section on pages Fig 1 on page 4, Figure 2 on page 10, figure 3 on page 15, and figure 4 on page 16, figure 5 on page 18, and Table 5 on page 21.
Comment #7. Discussion
• Strengths: The discussion connects findings to conservation and socio-economic issues.
• Weaknesses: There is insufficient engagement with contemporary literature. Key findings are not critically analyzed against previous studies.
• Recommendation: Incorporate a comparative analysis with global studies on forest degradation. Critically evaluate the implications of key results, such as the role of local governance in conservation efforts.

Response # 7
We thank the reviewer for this important suggestion to deepen the analytical strength of the discussion. In response:
•We have substantially revised the discussion section to include a broader engagement with recent and relevant global literature on forest degradation, land use change, and population dynamics. Comparative insights from studies in other tropical biosphere reserves and conservation areas (e.g., Southeast Asia, Central Africa, and Latin America) have been incorporated to contextualize our findings.
•We have critically examined how our results align with or diverge from previous studies, particularly regarding the drivers of forest loss and the socio-economic dimensions of resource pressure.
•Moreover, we have expanded our evaluation of the role of local governance in forest conservation. This includes a discussion of community-based forest management, institutional challenges, and the effectiveness of local bylaws and stakeholder engagement, supported by recent studies and best practices from similar settings.
These additions provide a more nuanced understanding of our findings and better position them within the global discourse on forest conservation. The revised discussion is found on pages 7- 20
Comment # 8. Recommendations
Strengths: The recommendations are actionable and community-oriented.
Weaknesses: They are broad and lack prioritization.
Recommendation: Categorize recommendations into immediate, medium-term, and long-term actions. Justify recommendations with data and evidence from the study.

Response # 8
We thank the reviewer for the constructive feedback and for recognizing the community-oriented nature of our recommendations. In response:
•We have revised the recommendations section to categorize proposed actions into immediate, medium-term, and long-term interventions. This prioritization provides a clearer implementation framework for stakeholders.
•Each recommendation is now more explicitly supported by data and findings from our study—for example, deforestation hotspots identified through satellite analysis and community perceptions gathered through surveys and interviews.
•We also clarified the linkage between the proposed actions and the underlying causes

of forest degradation identified in the research, ensuring that each recommendation is both evidence-based and aligned with the study objectives.
These improvements are presented in the revised conclusion and recommendations sections on pages 23, and are highlighted accordingly in the updated manuscript.
Comment # 9: Alignment of Research Problem, Objective, Methods, Results, and Discussion
Strengths: The manuscript maintains alignment throughout its sections.
Weaknesses: The linkage between objectives, findings, and recommendations is weak. For example, the methods section does not explain how the data supports specific recommendations.
Recommendation: Create explicit connections between each section. For instance, demonstrate how the satellite imagery data directly informs the policy recommendations.

Response # 9
We thank the reviewer for highlighting the importance of internal consistency and coherence across the manuscript. In response:
•We have revised the methods and discussion sections to more clearly explain how each type of data collected—particularly the satellite imagery and socio-economic survey results—contributes directly to the study's objectives and informs the recommendations.
•The discussion now includes explicit references to how land cover change data (e.g., forest loss from 1984 to 2024) supports targeted policy interventions such as zoning, reforestation efforts, and land use planning.
•We have also refined the recommendations section to include cross-references to the specific findings they are based on. This creates a stronger narrative thread from research problem to objective, methods, results, and actionable solutions.
•Where applicable, we included a summary table that links each research objective with the corresponding data, key findings, and resulting recommendations to make these connections more transparent to the reader.
These improvements appear on pages 18 to 21
Comment # 10: Suggestions for Authors
o Refine the introduction to frame the study within a global conservation context.
o Enhance methodological transparency by justifying data collection and analysis choices.
o Integrate more critical analysis in the discussion, contrasting findings with global studies.
Response # 10
We sincerely thank the reviewer for these valuable and constructive suggestions. In response:
•We have refined the introduction to more clearly position the study within a global conservation context. This includes discussion of the relevance of biosphere reserves to global biodiversity conservation, climate change mitigation, and sustainable development goals. We have also added citations to recent international studies and frameworks to support this global framing.
•To enhance methodological transparency, we have added justifications for our choices in data collection (e.g., selection of satellite imagery years, survey tools) and data analysis (e.g., use of SPSS, regression analysis). Supporting literature has been cited to provide methodological grounding and demonstrate adherence to established protocols.
•The discussion section has been revised to incorporate more critical analysis, with a comparative perspective that contrasts our findings with similar research conducted in other tropical and subtropical forest regions globally. This includes discussion of consistencies, divergences, and the broader implications of our results in terms of forest governance and conservation policy.
These revisions are reflected in the updated manuscript on pages 7 to 21, and the changes have been clearly marked for ease of review.
We would like to thank the editor and reviewers once again for their constructive comments and valuable suggestions. We believe the revisions have substantially improved the clarity, rigor, and relevance of our manuscript. All changes have been carefully addressed and are highlighted in the revised version. We hope the

	manuscript now meets the journal's standards and expectations, and we respectfully submit it for your further consideration.<br>Sincerely,<br>Fikru Mosisa Hunde (Assistant professor)<br>On behalf of all authors<br>Department of Biology, College of Natural Sciences, Mattu University, Ethiopia<br>fikrumosisa@gmail.com
Additional Information:	
Question	Response
**Financial Disclosure**<br><br>Enter a financial disclosure statement that describes the sources of funding for the work included in this submission. Review the submission guidelines for detailed requirements. View published research articles from *PLOS ONE* for specific examples.<br><br>This statement is required for submission and **will appear in the published article** if the submission is accepted. Please make sure it is accurate.<br><br>**Funded studies**<br>Enter a statement with the following details:<br>• Initials of the authors who received each award<br>• Grant numbers awarded to each author<br>• The full name of each funder<br>• URL of each funder website<br>• Did the sponsors or funders play any role in the study design, data collection and analysis, decision to publish, or preparation of the manuscript?<br><br>Did you receive funding for this work?	The author(s) received no specific funding for this work.
**Competing Interests**<br><br>Use the instructions below to enter a competing interest statement for this submission. On behalf of all authors, disclose any competing interests that could be perceived to bias this work—acknowledging all financial support and any other relevant financial or non-financial competing interests.	The authors have declared that no competing interests exist.

This statement is required for submission and **will appear in the published article** if the submission is accepted. Please make sure it is accurate and that any funding sources listed in your Funding Information later in the submission form are also declared in your Financial Disclosure statement.<br><br>View published research articles from *PLOS ONE* for specific examples.<br><br>**NO authors have competing interests**<br><br>Enter: *The authors have declared that no competing interests exist.*<br><br>**Authors with competing interests**<br><br>Enter competing interest details beginning with this statement:<br><br>*I have read the journal's policy and the authors of this manuscript have the following competing interests: [insert competing interests here]*<br><br>* typeset	
**Ethics Statement**<br><br>Enter an ethics statement for this submission. This statement is required if the study involved:<br><br>• Human participants<br>• Human specimens or tissue<br>• Vertebrate animals or cephalopods<br>• Vertebrate embryos or tissues<br>• Field research<br><br>Write "N/A" if the submission does not require an ethics statement.<br><br>General guidance is provided below. Consult the submission guidelines for detailed instructions. **Make sure that all information entered here is included in the Methods section of the manuscript.**	258/Biol./2024

**Format for specific study types**<br><br>**Human Subject Research (involving human participants and/or tissue)**<br>• Give the name of the institutional review board or ethics committee that approved the study<br>• Include the approval number and/or a statement indicating approval of this research<br>• Indicate the form of consent obtained (written/oral) or the reason that consent was not obtained (e.g. the data were analyzed anonymously)<br><br>**Animal Research (involving vertebrate animals, embryos or tissues)**<br>• Provide the name of the Institutional Animal Care and Use Committee (IACUC) or other relevant ethics board that reviewed the study protocol, and indicate whether they approved this research or granted a formal waiver of ethical approval<br>• Include an approval number if one was obtained<br>• If the study involved *non-human primates*, add *additional details* about animal welfare and steps taken to ameliorate suffering<br>• If anesthesia, euthanasia, or any kind of animal sacrifice is part of the study, include briefly which substances and/or methods were applied<br><br>**Field Research**<br><br>Include the following details if this study involves the collection of plant, animal, or other materials from a natural setting:<br>• Field permit number<br>• Name of the institution or relevant body that granted permission	
**Data Availability**<br><br>Authors are required to make all data underlying the findings described fully available, without restriction, and from the time of publication. PLOS allows rare exceptions to address legal and ethical concerns. See the PLOS Data Policy and FAQ for detailed information.	Yes - all data are fully available without restriction

A Data Availability Statement describing where the data can be found is required at submission. Your answers to this question constitute the Data Availability Statement and **will be published in the article**, if accepted.<br><br>**Important:** Stating 'data available on request from the author' is not sufficient. If your data are only available upon request, select 'No' for the first question and explain your exceptional situation in the text box.<br><br>Do the authors confirm that all data underlying the findings described in their manuscript are fully available without restriction?	
**Describe where the data may be found in full sentences. If you are copying our sample text, replace any instances of XXX with the appropriate details.**<br><br>• If the data are **held or will be held in a public repository**, include URLs, accession numbers or DOIs. If this information will only be available after acceptance, indicate this by ticking the box below. For example: *All XXX files are available from the XXX database (accession number(s) XXX, XXX.*).<br>• If the data are all contained **within the manuscript and/or Supporting Information files**, enter the following: *All relevant data are within the manuscript and its Supporting Information files.*<br>• If neither of these applies but you are able to provide **details of access elsewhere**, with or without limitations, please do so. For example:<br><br>*Data cannot be shared publicly because of [XXX]. Data are available from the XXX Institutional Data Access / Ethics Committee (contact via XXX) for researchers who meet the criteria for access to confidential data.*<br><br>*The data underlying the results presented in the study are available*	All relevant data are within the manuscript and its Supporting Information files.

from (include the name of the third party and contact information or URL).<br>• This text is appropriate if the data are owned by a third party and authors do not have permission to share the data.<br><br>* typeset	
Additional data availability information:	Tick here if the URLs/accession numbers/DOIs will be available only after acceptance of the manuscript for publication so that we can ensure their inclusion before publication.

Powered by Editorial Manager® and ProduXion Manager® from Aries Systems Corporation

Manuscript Click here to access/download;Manuscript;R1_manuscript.docx

IMPACT OF POPULATION PRESSURE ON FOREST RESOURCES DEPLETION IN YAYO COFFEE FOREST BIOSPHERE RESERVE, SOUTHWEST ETHIOPIA

Fikru Mosisa [1,*], Adanech Asfaw [2], and Tefera Jogora [2]

[1]*Department of Biology, College of Natural Science, Mattu University, Mattu, Ethiopia*

[2] *Department of Forestry, College of Agriculture and Forestry, Mattu University, Mattu, Ethiopia*

[*]*Corresponding Author: Email: fikrumosisa@gmail.com, phone number: +251917851910, P. O. Box: 381*

ABSTRACT

Forests regulate the climate and support rich biodiversity, but growing population pressure threatens these vital functions through deforestation and habitat loss. The research was carried out in the Yayo Coffee Forest Biosphere Reserve, located in the Oromia region of southwest Ethiopia. Data were gathered from key informants, kebele leaders, development agents, selected households, focus group discussions, and field observations. Land use and land cover change data were obtained from satellite imagery spanning 1984 to 2024. Population growth data were sourced from the Jimma branch of the Ethiopian Central Statistical Agency. Additionally, field visits included direct observations of the forest's condition. A total of 148 participants responded to both structured and semi-structured questionnaires designed to address the study's objectives. The communities in the study area do not rely on a single livelihood strategy but instead engage in various activities such as crop cultivation, forestry, livestock rearing, and off-farm work, with varying levels of dependence on each. Among the forest products collected, coffee, firewood, honey, and wild spices were the most common. The main factors contributing to forest degradation were ranked as agricultural expansion (23.13%), lack of local participation (21.95%), timber extraction (15.27%), urbanization (14%), effects of climate change (13.46%) and investment (12.2%) in decreasing order. Satellite image analysis revealed a negative correlation between population growth and forest cover, with a significant difference at $P<0.05$. The study highlights the impact of population pressure on forest resource degradation in the Yayo Coffee Forest Biosphere Reserve. Therefore, key players in forest management and biosphere reserve

implementation, policy makers, and community members should focus on risk mitigation and on sustainable management, conservation, and utilization of the biosphere reserve.

Key words: *degradation; forest management; habitat loss; population pressure; land use change*

1 INTRODUCTION

Forests play a crucial role in keeping life on earth intact as the world's lungs, taking in carbon dioxide and expelling oxygen, which keeps the climate stable [1, 2]. They shelter incredible diversity of flora and fauna, with over 80% of land-based biodiversity in the world [2]. Besides keeping ecosystems intact, forests are needed for water purification, preventing soil erosion, and flood regulation. For millions, especially rural and indigenous populations, forests are a critical source of livelihood, food, fuel, medicine, and much more [3]. Forests hold cultural, spiritual, and recreational importance for communities worldwide [4]. The concept of biological carrying capacity, which defines the maximum population that an environment can sustain on a sustainable basis, is widely used to study the impact of demographic change on natural resources [5]. This capacity depends on a range of factors including economic development, socio-political processes, technology, trade, and patterns of consumption [6].

Sub-Saharan Africa land degradation in most areas is a result of a combination of direct and indirect human intervention [7]. Direct causes includes deforestation, overgrazing, unsustainable timber extraction, shifting cultivation and mismanagement of water and soil resources [3, 8, 9] These are compounded by inadequate conservation measures, poorly chosen crop rotations, marginal land use for agriculture, improper fertilizer application, inefficient irrigation, and excessive groundwater abstraction. [10]. Indirect causes include high population growth rates, restricted availability of land for cultivation, insecure tenure regimes, poverty, and pervasiveness of economic hardship [11].

Ethiopia is a limited exception where deforestation, after acute growth in population with low socio-economic development, has led to serious environmental degradation [9, 11]. Forest cover that once covered approximately 40% of land area in the early 1900s has drastically declined to less than 2.2% in recent decades [11 - 13]. The Yayo Coffee Forest Biosphere Reserve plays a critical role in carbon sequestration, supports international conservation of biodiversity, and aids global climate and sustainability agendas such as the SDGs and the Paris Agreement

[14]. Reforestation efforts under the Green Legacy Initiative have nonetheless been an influence that has witnessed modest forest cover increases, rising to approximately 6.4% from 17.2% in 2019 to 23.6% in 2023 [15]. Despite ongoing conservation efforts within the Yayo Coffee Forest Biosphere Reserve, there remain current challenges such as low community engagement and inadequate linkage of local livelihoods [16]. The majority of restoration initiatives aim at ecological goals while neglecting socio-economic needs [17]. Therefore, this research aims to overcome the impacts of population pressure on forest resource depletion in the Yayo coffee forest biosphere reserve, Ilubabor Zone, Oromia Regional State, Southwest Ethiopia.

2 METHODS AND MATERIALS

2.1 Study area description

The Yayo Coffee Forest Biosphere Reserve is located in the Ilu Abba Bora Zone of the Oromia Regional State in southwestern Ethiopia. It is recognized as the center of origin for Coffea arabica, the world's most widely consumed coffee, and Yayo is the largest and most significant forest for conserving wild coffee populations globally [18]. The reserve plays a crucial role in preserving both natural and cultural landscapes [19]. Situated in the southwestern part of the Oromia region, the Yayo Biosphere Reserve includes the Woredas of Hurumu, Yayo, Chora, Nopha, Alge Sachi, and Doreni, covering coordinates from 8°0'42" to 8°44'23" N and 35°20'31" to 36°18'20" E [Fig. 1, "Reprinted from Abera *et al.*, 2021 under a CC BY 4.0 license, with permission from Elsevier, original copyright 2021].

The district's elevation ranges from 1,139.2 to 2,581.9 meters above sea level, with the lowest point located at Gaba River (1,100 meters) and the highest point at the summit of Sayi Mountain (2,581 meters) in Keresi. The area experiences a hot and humid climate, with an average annual temperature of around 23°C, ranging from a mean minimum of 18.59 °C to a mean maximum of 27.88 °C [20]. The varied physical conditions and altitudinal differences contribute to a rich diversity of climate, soil, and vegetation, fostering the development of numerous plant species with a high level of diversity [21]. The rainfall pattern of the districts varies annually from 1,191.6 to 1,960.7 mm, showing variations from year to year. It is a unimodal type of rainfall that increases from May to October and declines in November [22].

The study focuses on the Hurumu, Yayo, and Doreni districts located in the Yayo Coffee Forest Biosphere Reserve in southwestern Ethiopia, which is famous for its ecological importance and genetic diversity of wild Coffea arabica [23]. The biosphere reserve has a total surface area of 167,021 hectares and consists of three zones: a core zone (27,733 ha), a buffer zone (21,552 ha), and a transition zone (117,736 ha), balancing conservation and sustainable development [24]. Land in the study area comprises 3.5% highland (5,750.4 ha), 85% temperate zone (138,465.85 ha), and 11.47% lowland (18,684.75 ha), reflecting its heterogeneity and altitudinal gradients [25]. This diversity accounts for much of the biodiversity of the region and explains its status as a key location for environmental studies and resource management [26].

The diverse climatic conditions and habitats in these districts have contributed to a high level of species diversity in both plants and animals [27]. This biodiversity richness is one of the reasons why Ethiopia is considered one of the 20 most biodiverse countries in the world [28].

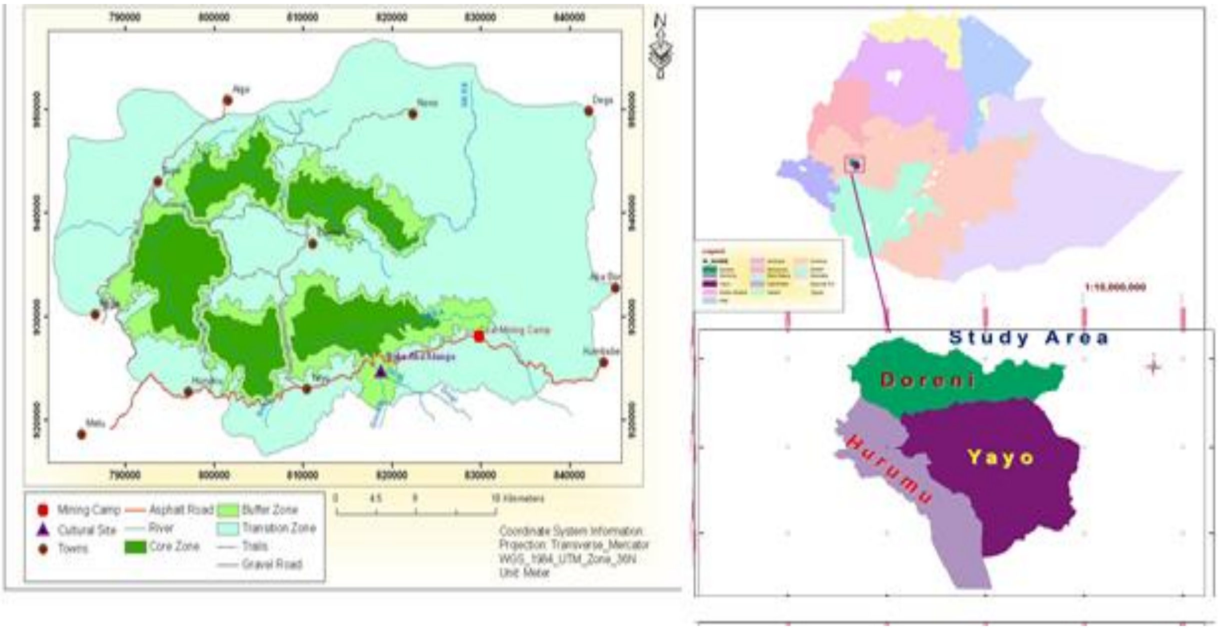

Fig. 1: Maps of the study area [“Reprinted from [Abera *et al.*, 2021] under a CC BY 4.0 license, with permission from Elsevier, original copyright 2021.”]

2.2 Study Design

This research employs a mixed-methods research design, integrating both quantitative and qualitative approaches to comprehensively investigate the impacts of population dynamics on the

Yayo Biosphere Reserve. The design is structured to systematically address the following objectives: assessing population growth, evaluating forest cover change, examining conservation practices, understanding community resource relationships, identifying stakeholder roles, and proposing sustainable management strategies.

2.3 Data collection

Following ethical approval, the study was conducted in several data collection waves between May 2024 and January 2025. The data for this study includes a sample household survey and land use/land cover change data. The research approach involves a consultative and interactive process, engaging respondents who are willing to provide essential information [29]. Interviews were conducted to collect data on the role of local communities in forest conservation, the impacts of human activities on the forest, and perceptions toward conservation efforts, as well as the challenges and consequences associated with these activities [30]. To collect data, formal survey questionnaires were used to gather quantitative information from selected households [31]. Focus group discussions and interviews were conducted to collect primarily qualitative data. Satellite imagery was utilized to generate land use land cover change data [32].

2.4 Population

The population of interest for this study comprised rural households and key stakeholders residing in and around the Yayo Coffee Forest Biosphere Reserve, specifically within the selected woredas of Yayo, Hurumu, and Doreni. During the study time, there were a total of 91,694 households; 89,039 were males and 2,659 were females, with a total population of 458,472 individuals. These communities were chosen due to their direct dependence on forest resources for their livelihoods and their proximity to the core and buffer zones of the biosphere reserve [33, 34].

The sampling frame included residents from nine kebeles, three from each of the selected woredas, identified based on geographic location, accessibility, and relevance to forest conservation activities [35]. Households were selected using purposive sampling techniques to ensure the inclusion of individuals with lived experience in forest resource use and conservation [36]. Criteria such as length of residence in the area, proximity to the forest, and knowledge of local forest management practices were considered during the selection process [37].

2.5 Sample Size and Sampling Techniques

To ensure a comprehensive understanding of the study area, a combination of preliminary assessments and quantitative and qualitative surveys was employed [38]. An initial exploratory survey was conducted to gain an overview of the spatial distribution of the Yayo Coffee Forest Biosphere Reserve and to facilitate the selection of appropriate study sites [23]. The Yayo Coffee Forest Biosphere Reserve encompasses six woredas of the Ilubabor and Bunobedele zones, namely Yayo, Hurumu, Chora, Bilo Nopha, Alge Sachi, and Doreni. From the foregoing, for the present study, Yayo, Hurumu, and Doreni were utilized as representative samples [39]. Among these sampled woredas, three rural kebeles were selected randomly, and nine kebeles were ultimately selected in the end: Haro, Gaba, and Wangegne of Hurumu woreda; Waboo, Geci, and Wixete of Yayo woreda; and Bocho, Badesa, and Henna of Doreni woreda. These kebeles were chosen on purpose since they are nearest to the forest zone. Respondents in these kebeles were chosen according to purposive criteria, including location near the forest, residence in the biosphere reserve for years, and knowledge on forest resource use and conservation methods [39].

To gather quantitative information, a systematic household survey was conducted with 148 respondents from the chosen kebeles. The survey collected baseline data on household characteristics, land use, livelihood strategies, and perceptions towards forest conservation [40, 41]. At the same time, qualitative information was gathered through key informant interviews and FGDs. 18 key informants, two from each kebele, were interviewed. These informants included village officials, kebele administration, development agents (DAs), and the relevant subject-matter experts. Their feedback provided contextual depth to the quantitative data and enriched the analysis as a whole [42]. Three FGDs were also organized to garner community-level perspectives regarding forest management and socio-economic issues [43]. Three FGDs were made, each containing seven members, one from each woreda, resulting in a total of 21 participants. These interviews enriched the qualitative data through the injection of locally grounded knowledge and lived experience [44].

2.6 Data Analysis

The quantitative data collected through household surveys were first cleaned, validated, and coded in Microsoft Excel to promote consistency and accuracy [45]. The cleaned data were then exported into SPSS version 20 for analysis. Descriptive statistics were used in summarizing key variables such as household characteristics, forest resource use, and conservation knowledge [46].

To analyze causal relationships, multiple linear regression and logistic regression tests were used. These methods allowed the assessment of the impact of independent variables, i.e., household size, income level, and education, on dependent variables like dependency on forest resources and conservation behavior [47]. The validity of the regression models was checked by using diagnostic tests [48].

For the qualitative data collected through key informant interviews and focus group discussions, thematic analysis was applied [49]. The data were coded, transcribed, and categorized into local perceptions, emerging themes, traditional knowledge, forest governance, and livelihood strategies. Representative statements of the participants were utilized to elaborate on these themes [50]. Through this mixed-methods approach with regression-based quantitative analysis complemented by rich qualitative feedback, there was a guaranteed holistic understanding of the drivers, effects, and community perspectives about the use and preservation of forest resources [51].

2.7 Ethical Considerations

The study was conducted in accordance with ethical standards, with ethical clearance granted by the Institutional Research and Ethics Review of Mattu University. Participants were informed about the study's purpose, procedures, and voluntary nature, and their identities were kept confidential. Participation was voluntary, with the option to withdraw at any point without consequence. No financial or material incentives were provided to influence participation. The research tools and interview protocols were culturally sensitive and respectful of local customs and norms. Permission to conduct the study was obtained from local authorities and community leaders, particularly in selected kebele and woreda. Data was securely handled and stored to protect participants' privacy, and access was restricted to the research team only.

3 RESULTS AND DISCUSSION

3.1 Socio-economic characteristics

The majority of the households, 89,039 (97.1%), were male-headed, while only 2,659 (2.9%) were female-headed. The ages of the respondents ranged from 20 to 80 years, with a mean age of 48 years. The family size varied between 1 and 12 members, with an average of 5.18, which is consistent with the national average family size. Regarding education, 15.9% of the respondents

were illiterate (unable to read or write), 30.4% had attended grades 1-5, 42% had completed grades 6-12, and 11.6% had completed secondary. On income, 39.8% of the households were low-income, 35.1% middle-income, and only 10.8% high-income. In terms of household size, the largest share (55.4%) included 4–5 members, then 23.6% included 6–7 members, and 12.8% included over 7 [Table 1]. Similarly, the family's socioeconomic status includes the household income, earners' education and occupation, as well as combined income when their own attributes are assessed [52]. In fact, the socio-economic status can be measured in a number of different ways, and most commonly, it is measured by education, occupation, and income [53]. Family illiteracy drastically restricts economic opportunities via limited availability of well-paying employment, resulting in poverty; affects the health services; and is unlikely to possess the capacity to aid their education [54- 56].

Table 1: Socio-demographic and economic characteristics of the participants among households in Yayo coffee forest Biosphere Reserve, Southwest Ethiopia, 2024.

Variables	Category	Frequency	Percent (%)
Sex	Male	144	97.2
	Female	4	2.8
	Total	148	100.0
Age	20-30	4	2.9
	31-40	58	39.1
	41-50	34	23.2
	51-60	22	14.5
	>61	30	20.3
	Total	148	100.0
Educational status	Illiterate (no formal education)	24	15.9
	Grade 1-5	45	30.4
	Grade 6-12	62	42.0
	Diploma & above	17	11.6
	Total	148	100.0
Income level	No income	21	14.2
	Low income	59	39.8
	Middle income	52	35.1
	High income	16	10.8
	Total	148	100
Family size	≤ 3	12	8.0
	4–5	82	55.4
	6 –7	35	23.6
	> 7	19	12.8
	Total	148	100.0

3.2 Livelihood strategies

In this study, among the sample households that participated in the survey, 37.7% of respondents indicated that their income came from crop production, 22.7% from forest products, and 18.7% from livestock production. Additionally, 27.5% reported that their income sources were crop production and forest products. The remaining 8.7% of respondents indicated that they relied on all types of household income-generating activities (Fig.2). Against the above result, 73% of forest dwellers depend on these products as a source of revenue in the Bale Zone, southern Ethiopia [57]. The value of income sources like forest products, crop production, livestock production, and off-farm activities depends on each other [58]. Research conducted in the adjacent district of Yayo, included under one National Forest Priority Area

(NFPA) with Gabba-Dogi, i.e., Yayo NFPA [59], revealed that 92.6 percent of the population in the study area have coffee in the forest, from which 57.3 kg of honey on average is harvested per household per year [59, 60].

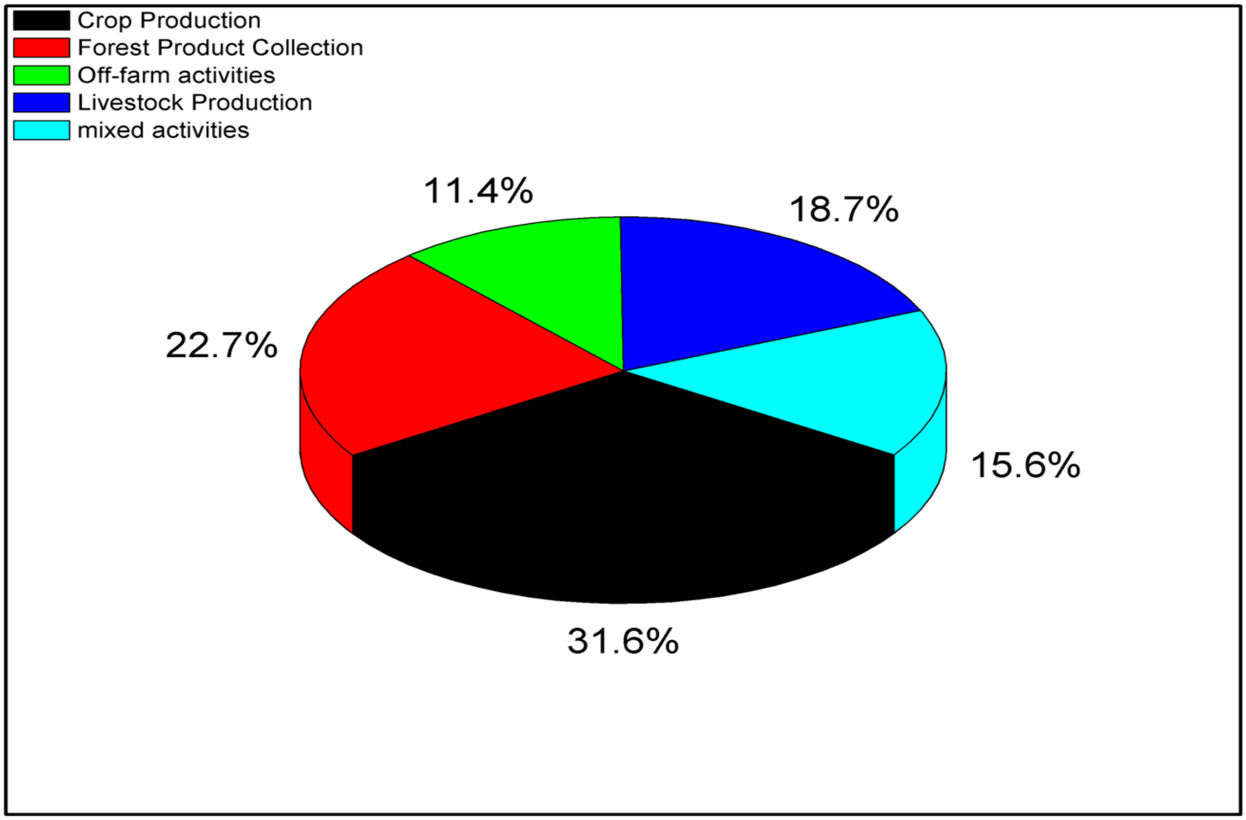

Fig. 2: Livelihood strategies among study participants in Yayo coffee forest Biosphere Reserve, Southwest Ethiopia, 2024

3.3 Formal Institutions in forest management

Based on the information gathered from interviews and focus group discussions, various institutions at the federal, regional, and local levels were identified, along with their interlinkages concerning the management of the coffee forest resource under investigation[61].

Changes in the institutional structure of the Ministry of Agriculture (MoA) since the early 1990s have failed to establish a dedicated government body for the management, conservation, and sustainable use of coffee forests [18]. This gap has contributed, to some extent, to the increasing lack of effective institutions for the sustainable management of coffee forests. Federal institutions,

mainly under the Institution for Biodiversity Conservation (IBC), provide technical support for Yayo Coffee Forest Biosphere Reserve conservation [62].

At the regional level, two institutions with potential connections to the coffee forest are examined: the Oromia Forest and Wildlife Enterprise Supervising Agency and the regional agriculture and rural development bureaus established in 1999 under proclamation 90/1999 as parts of decentralization [52]. The primary issues identified within these organizations include a lack of technical and direct focus on forest coffee biodiversity conservation, insufficient budget and technical personnel at the agricultural and rural development bureaus, inadequate decentralization of the budget, and limited community involvement in planning and execution at the Forest Enterprise Supervising Agency [63]. Another gap noted in the State Forest Enterprise is the absence of incentives to motivate local communities to conserve specific forests [64]. The study confirms that local indigenous institutions have been gradually marginalized due to the increasing control of the state, which enforced formal institutions under various regimes in the past [65].

At the local level, the institutions involved in the use, conservation, and management of the coffee forest include the Yayo Coffee Forest Biosphere Reserve Conservation Project, the district administration (comprising the kebele and development team), and the district [66].

3.4 Informal institutions contributing for forest management.

The study examines various informal institutions in terms of their structure and role in the livelihoods of the community in the study area. These institutions are categorized into four groups, with particular emphasis placed on two clusters [67]. The territorial-based administrative indigenous/customary institutions are further divided into four groups: Tuullaa, Xuxee, Shane, and Jaarsa Biyya, along with Muchoo. The study conducted by Tulu and Getahun revealed the same result with current study. The first cluster consists of territorial-based administrative indigenous/customary institutions, while the second includes a variety of self-help work organizations [68].

Shane, Xuxee, and Tuullaa make two important contributions to the management of coffee forests. A fundamental role in bringing local governance systems into alignment is played by Tuullaa, which organizes, leads, and enforces the work, norms, and regulations of various local customary institutions and self-help labor associations. By controlling coffee harvesting and enforcing customary regulations to stop overexploitation and resource degradation, for example, these organizations manage coffee forests both directly and indirectly [69]. This finding aligns with the broader literature on common-pool resource management, which emphasizes that strong social norms, particularly those founded on reciprocity and generalized trust, are instrumental for the sustainable management

and conservation of collective natural resources. The result highlights the central role of Tuullaa as a culturally grounded institution that governs not only the social and economic existence of the community in the coffee forest zone but also exercises a significant influence over governing the environment [70].

The ability to govern conduct among community members and facilitate the crafting and enforcement of local rules situates it as a significant mechanism for maintaining collective action for sustainable forest use. This is in harmony with common-pool resource governance theory, which praises the effectiveness of locally crafted institutions in shared ecosystem management [71].

Jaarsa Biyya and Muchoo, as customary Oromo institutions, play a crucial role in the sustainable management of the coffee forest and other natural resources by being local enforcers of customary law and mediators in resource disputes. Their authority, which has its foundation in respect by the community, allows them to enforce existing rules and, if supported by the locals, create, and implement new rules. This aligns with the contention that locally embedded institutions are more effective and legitimate in the governance of resources than externally introduced formal institutions [72]. Similarly, it contends for the importance of facilitating resource users to participate in rule-making, a function these institutions are strategically positioned to undertake [73]. This is also supported by who adds that local self-help associations among the Oromo have traditionally worked to improve food security and economic resilience [74 - 75]. Interpreted through the theory of institutional bricolage, Jaarsa Biyya and Muchoo demonstrate how traditional authority can be reshaped to contemporary challenges, blending cultural practice with evolving governance imperatives for promoting sustainability [76].

3.5 Conservation policies and its drawbacks

As indicated by various informants in the coffee forest study area, different forms of ownership rights existed prior to the demarcation process. The publicly owned and strictly conserved portion of 50,000 hectares of the Yayo Coffee Forest Biosphere Reserve is the most significant. The remaining 117,000 hectares fall under a mix of community, cooperative, and private regimes, which work to strengthen traditional forest coffee production. Such sites support over 150,000 people, with 40–50% of the reserve in mixed regimes [77]. Federal Forest Development, Conservation, and Utilization Proclamation no. (5, 6) has a sure implication for natural resource

management, even though in the practical implementation they have faced their own limitation [78].

3.6 The contributions of the Biosphere reserve to the local communities

In this study, the collection of forest products was identified as the primary source of household income. Approximately 43.9% of respondents stated that they are highly dependent on forest products to support their households. Additionally, 37.8% of the households surveyed indicated that collecting forest products is their top priority for sustaining their livelihoods, While 16.2% of the respondents said forest product collections are serving my household as a second income source, and 12.8% of them have ranked forest products as the third option for their livelihood [Table 2]. This finding is similar with many studies conducted in different areas in Ethiopia [79]. For instance, in the Yayo district, the same study area, in the Sheka zone, and in the Bale Mountains, forest products were revealed as the primary source of income, with contributions of 54%, 49%, and 44.7%, followed by crop production [80, 81]. In contrast, other studies have indicated that forest products contribute as the fourth most important source of livelihood for households. For instance, at Liban Woreda, Borena, southern Ethiopia (32%), and at Gore District, southwest Ethiopia, similar agro-ecology (23%) [82, 83].

Table 2: Forest products priority to support the household's livelihood among study participants in Yayo coffee forest Biosphere Reserve, Southwest Ethiopia, 2024

Preference Category	No. of Respondents	Percentage (%)	Rank
General dependency on forest products	65	43.9%	1
Forest products as 1st priority	36	37.8%	2
Forest products as 2nd priority	24	16.2%	3
Forest products as 3rd priority	19	12.8%	4
Not engaged in forest products	4	2.7%	5
Total	148	100.0%	

3.7 The major factors of forest depletion

The analysis of the household survey results highlighted several factors contributing to the depletion of forest stocks in the Yayo Coffee Forest Biosphere Reserve. Among the households surveyed, approximately 23.13% of respondents identified agricultural expansion as a major factor in the depletion of forest resources due to the local population's reliance on primary activities such as wood logging, non-timber forest product collection, and farming. The findings in the Yayo Coffee Forest Biosphere Reserve from the survey identify three major causes of forest degradation: lack of indigenous community participation (21.95%), Timber extraction (15.27%) and Urbanization (14%). The Yayo Fertilizer Factory, which has removed about 115 hectares of forestland from the buffer zone of Yayo Biosphere Reserve, is a poor example of industrial investment, which 12.2% of respondents cited as one of the primary causes of forest degradation. This is consistent with research showing that industrial operations significantly speed up deforestation and pose serious risks to biodiversity [Fig.3]. These are strongly supported by findings in some well-accredited studies in comparable environmental and socio-economic contexts. For instance, it is projected that urban expansion would account for the conversion of over 2.5 million hectares of forests globally by 2030, primarily in developing nations, as a testament to the impact of urban spread on forest degradation [84].

The respondents' high agreement with the statement that "the lack of indigenous involvement results in forest depletion" indicates that 73.9% of respondents strongly agree with this statement, which has been confirmed [85]. Similarly, the 75.7% who identified timber extraction as a major cause aligns with previous studies and reports, which rank unsustainable and illegal logging among the leading drivers of forest loss [86, 87].

These trends emphasize the pressing need for more stringent environmental regulation and more environmentally friendly development practices. Moreover, the residents reported that the Yayo Fertilizer Factory displaced a high number of farmers from their farms, and they resorted to daily work as a source of survival [86, 88]. The research revealed that approximately 2,200 farming families were physically displaced, losing their farms, houses, and wild coffee farms as sources of livelihood to accommodate the factory's production [89].

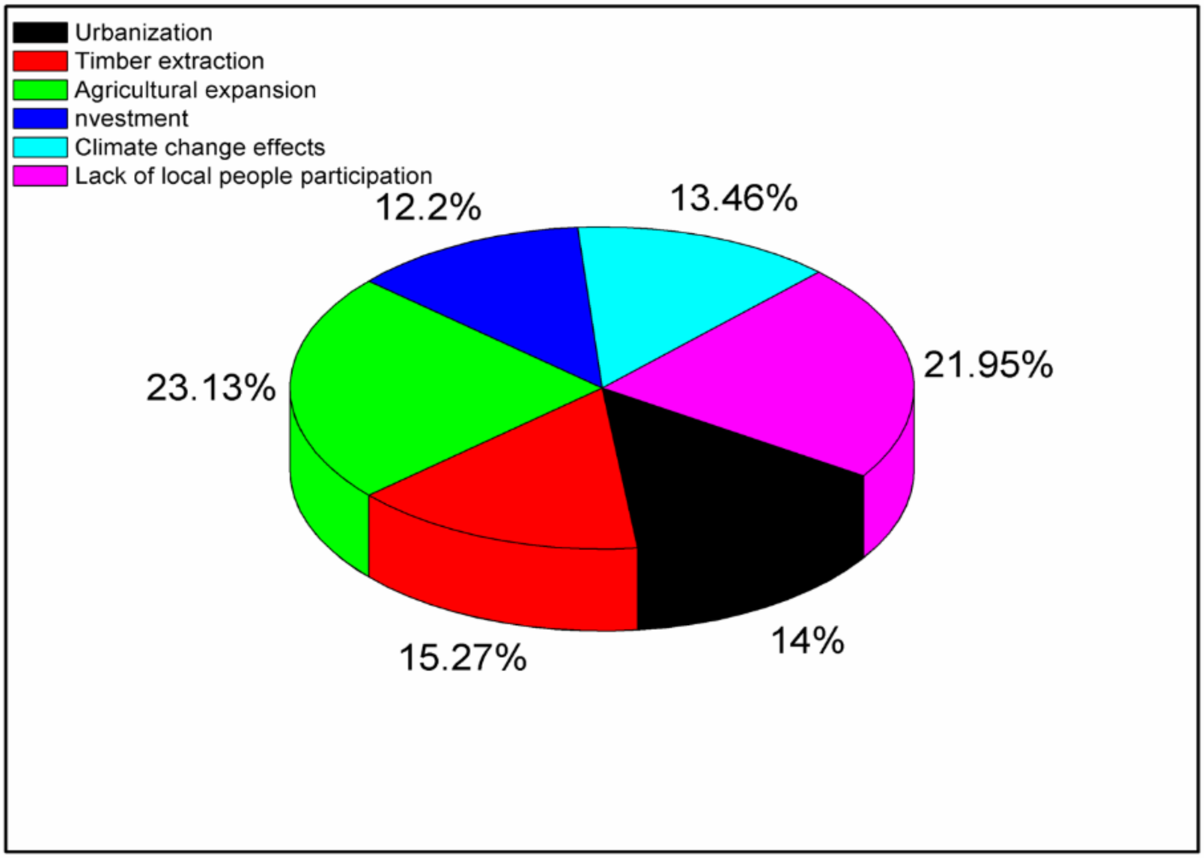

Fig. 2: Factors contributing for the possible depletion of forest stocks in the Yayo coffee forest Biosphere Reserve, Southwest Ethiopia, 2024.

Additionally, the crew saw firsthand how more than 26 low-income families depended on daily wage labor to make ends meet. The Community Forest Coffee Project's (COFCOP) arrival was considered noteworthy in this context because it provided some alternative livelihood support [90].

The coal mining initiative, launched in 2010, aimed to generate employment for thousands of jobless locals in the region. Between 2012 and 2013, over 6,000 skilled and unskilled workers were hired by the Yayo Fertilizer Factory, with most resettled in heavily forested villages such as Achebo and Wutete. However, starting in 2018, coal mining at the Achebo site came under the control of organized youth from displaced families. As a result of the project’s strict security measures, hundreds of villagers were physically displaced and denied access to their coffee farms, forest lands, and water sources. While compensation was provided, it was limited to losses of seasonal crops (like maize, sorghum, and barley), perennial crops (such as mango, pawpaw, and oranges), garden trees, and village homes and structures. The study also revealed that compensation was calculated for only 420 of the 2,200 displaced individuals. For example, compensation for a coffee stand was estimated at just 6.50 birr, and for one hectare of farmland, it was only 400 birr [90].

Other study results indicated a lack of economic opportunities and social amenities in rural areas; rural-urban migration has resulted in a considerable population increase in many sub-Saharan African cities [91].

3.8 Population growth and forest cover change

According to the statistical findings from the household survey, the main drivers of this activity were identified as farmland expansion (40.5%), forest product collection (29.6%), settlement (12%), and overgrazing (17.9%), with these factors emerging as the most significant contributors (Fig. 4). Ethiopia continues to have one of the highest population growth rates in sub-Saharan Africa, 2.5% to 3% annually, which adds pressure to land and other natural resources [92]. Over the past few decades, the region has been a big hub for migrants, with both rising numbers and rising ethnic diversity. Other groups who settled in the area include people from the Southern Nations, Nationalities, and Peoples' Region (SNNPR), Amhara, Tigray, and eastern Oromia [93]. Such movements relate to broader national patterns of domestic migration, often predicated on voluntary migration as well as government-sponsored resettlement programs aimed toward relatively sparsely populated forest zones [94].

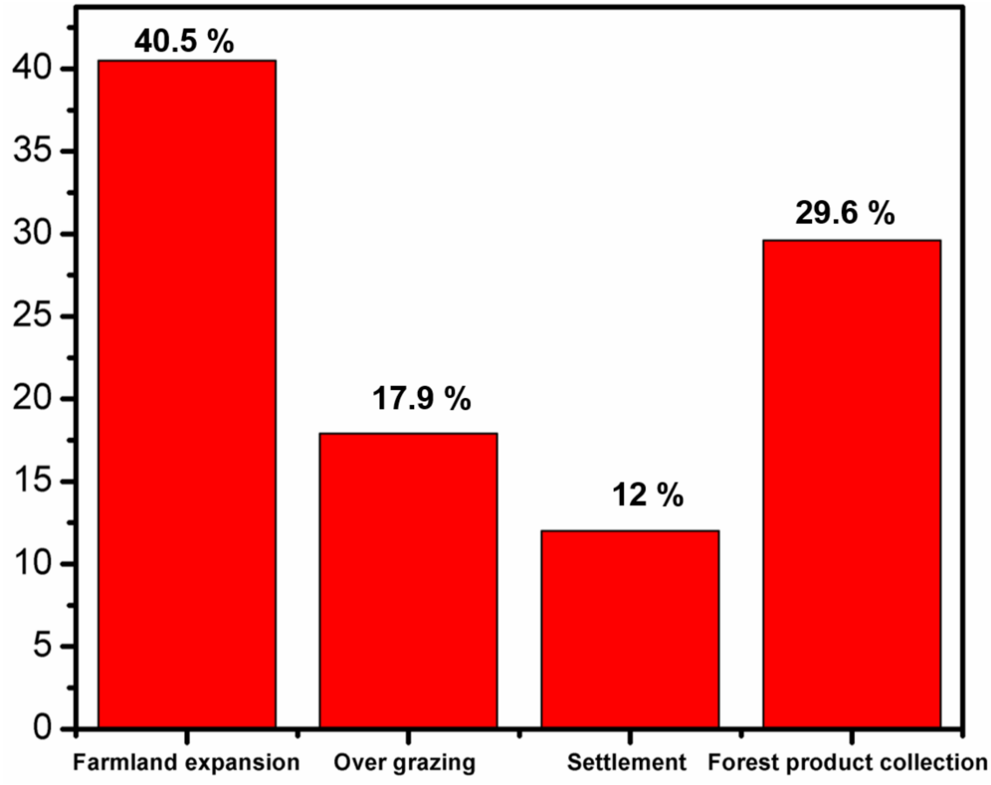

Fig. 3: Forest land Conversion in the Yayo coffee forest Biosphere Reserve, Southwest Ethiopia, 2024.

Household survey reports that 39.2% of the respondents view forest product collection, mainly firewood and forest coffee, as a leading cause of forest degradation. Hurumu Woreda informants report widespread overexploitation of tree species like Cordia africana for illegal timber [Fig. 5]. This aligns with general trends in Ethiopia and sub-Saharan Africa, where wood fuel and timber overextraction drive deforestation [95]. Cordia africana, of economic value for timber and shade, is most susceptible, threatening biodiversity and agroforestry. Despite its importance for livelihood, forest product collection accelerates degradation, particularly when combined with agricultural encroachment and settlement [96].

Farmland expansion, identified by 35.8% of respondents, is a leading cause of forest degradation in the area, while settlement activities contribute to 18.9% of the degradation [Table 5]. Overgrazing, however, is not as prominent since it entails the use of certain grazing fields (kaloo) that are different from forests. Similar findings in the Ethiopian highlands show that spatial land-use management, like exclosures, can avert degradation [97, 98]. In contrast, regions lacking these management systems, such as Afar and Borana, are facing severe soil degradation and loss of biodiversity, largely driven by uncontrolled overgrazing and the absence of sustainable land-use practices [99, 100].

3.9 Demographic Trends from 1984 to 2024 and forest cover change satellite image result

Population data for the past 40 years were obtained from the Central Statistical Agency (CSA), Jimma Branch, based on the Ethiopian Population Censuses conducted in 1984 and 1994, as well as projections from the 2007 census. According to the census data, the population of Ilubabor Zone was over 847,048 in 1984, over 970,243 in 1994, 1,271,609 in 2007 and 2,301,242 in 2024.The population of Yayo district increased from 29,837 in 1984 to 52,851 in 1994, then to 83,579 in 2007, and further to 139,001 by 2024. Similarly, the population of Hurumu district was 27,586 in 1984, rising to 42,667 in 1994, 106,294 in 2007, and 165,143 by 2024. In Dorani district, the population was 20,015 in 1984, increased to 36,705 in 1994, reached 58,938 in 2007, and grew to 97,491 by 2024 (Fig. 5).

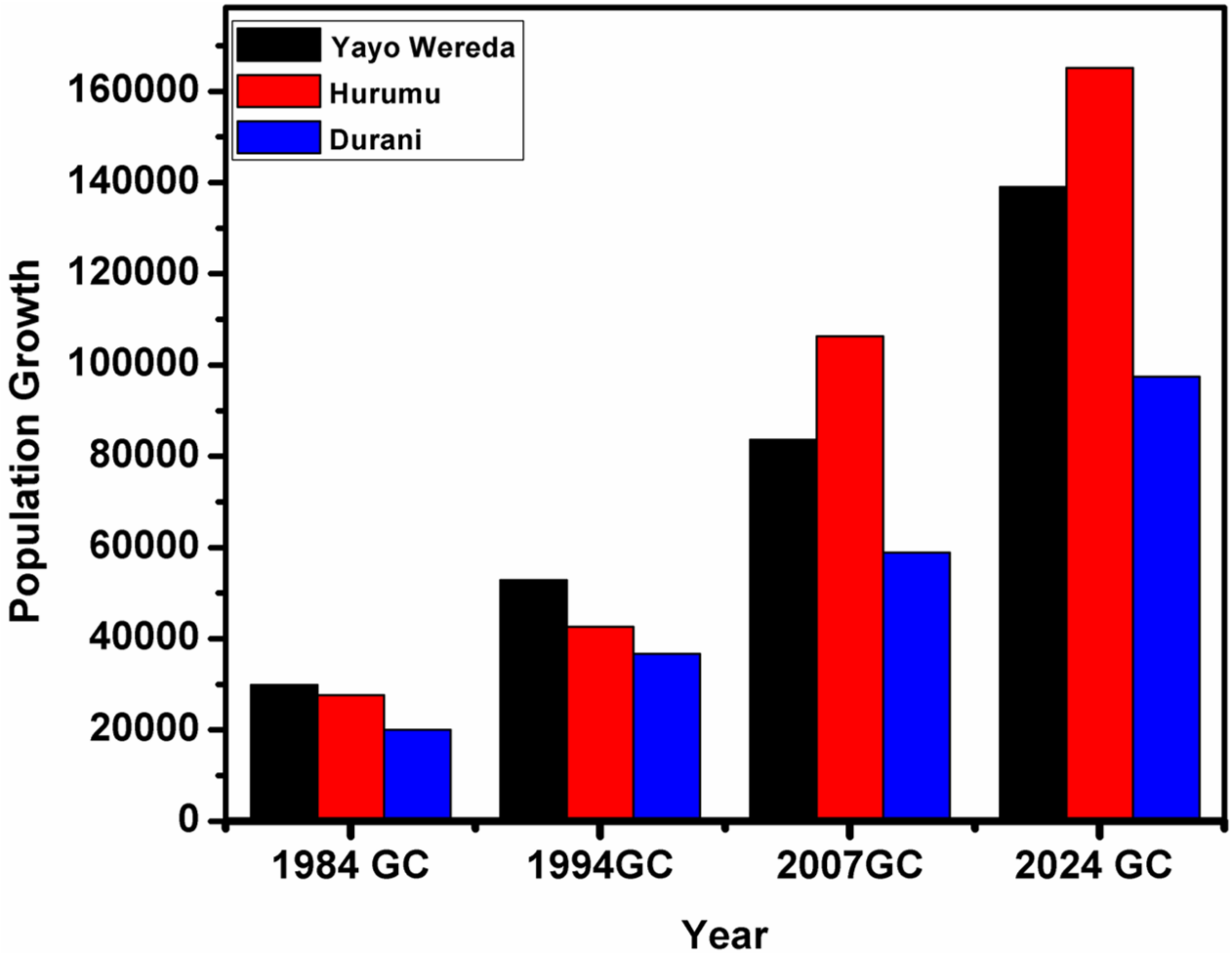

Fig.4: Demographic Changes in the Yayo coffee forest Biosphere Reserve from 1984 to 2024, Southwest Ethiopia [Yayo, Hurumu, and Doreni Districts]

The forest cover change in Yayo Biosphere Reserve over the past 40 years (from 1984 to 2024) is presented as a satellite image comparison. Landsat 5 TM for 1984, Landsat 5 TM and ETM for 1994, and Landsat 7 TM and ETM+ for 2024, each with a 30-meter resolution satellite image cover, showed Forest coverage decreased from 120087.2 hectares to 100772.9 hectares, or by 11.6%, over the 40-year period [Table 3]. Forest coverage is replaced by agricultural land, and settlement land is the dominant land use type in the study area, which is divided into three compartments: core, buffer, and transitional zones, within the Yayo Biosphere Reserve, and LANDSAT/TM satellite images from 1986 to 1990 show that Ethiopia's forest cover had since then been reduced to 3.93%, or 45,055 sq km [101, 102].

The Yayo Coffee Forest Biosphere Reserve is a significant ecological location that sequesters carbon and mitigates climate change, which makes it both a national and global priority [103]. Agroforestry systems in the region, particularly those for coffee grown under shade, are a

sustainable land use practice that conserves the environment and promotes local development and illustration of a climate-resilient approach in that they enhance carbon sequestration and sustain rural livelihoods [104].

The identification of these land use changes as significant contributors to the region's forest degradation further supported the findings of statistical analysis and satellite imaging, and it makes it exceedingly dangerous for the biosphere reserve to maintain the biodiversity that made it possible for it to be identified and About 15 households have unlawfully established on 8 hectares of the biosphere reserve's core zone, according to data acquired from focus groups and key informant interviews with farmers in Yayo District, particularly in the Kori area close to Sayi Forest [105]. Similarly, in Ilu Abba Dinka Kebele, Janeh area, about 28 households have settled on 14 hectares of the core zone. In total, approximately 22 hectares of forest in the core zone have been cleared due to agricultural expansion and coffee production. Additionally, in some areas, particularly in Gechi Kebele, local people have raised concerns about the planting of exotic tree species, which they believe could rapidly threaten and negatively affect the indigenous species by clearing the native vegetation [106].

The present results align with a study conducted in the Hawa Galan district of Kelem Wollega, Ethiopia, which found that forest cover in the district was negatively correlated with both the district's overall population and the population within the forest area [107]. Another study reported that population growth has intensified pressure on scarce natural resources, leading to the degradation and destruction of forests and woodlands [108].

Table 3: Satellite Images Results of Land Use Land Cover in the Yayo coffee forest Biosphere Reserve from 1984 to 2024).

**Districts**	**Land use land cover**	**1984**		**2004**		**2024**	
		Area (Ha)	%	Area (Ha)	%	Area (Ha)	%
Doreni	Forest	29030.1	63.1	25395.59	55.2	24889.52	54.1
	Agriculture & Settlement	16976.4	36.9	20610.91	44.8	21116.98	45.9
	Total	46006.5	100	46006.5	100	46006.5	100
Hurumu	Forest	30848.4	66.3	29638.65	63.7	24241.35	52.1
	Agriculture & Settlement	15680.1	33.7	16889.85	36.3	22287.15	47.9
	Total	46528.5	100	46528.5	100	46528.5	100
Yayo	Forest	60208.67	74.5	57703.34	71.4	51642.06	63.9
	Agriculture & Settlement	20608.34	25.5	23113.66	28.6	29174.94	36.1
	Total	80817	100	80817	100	80817	100

Grand total Forest land	120087.17	69.3	112737.58	65	100772.93	58.1

3.10 Socio-Economic Determinants of Forest Dependency and Conservation Behavior

The Pearson Correlation analysis results indicate a strong negative correlation between forest cover and population growth rate over the past 40 years in the surveyed forest biosphere reserve, with a coefficient value of -0.998. This negative value confirms an inverse relationship between population growth and forest cover, meaning that as population growth increases, forest area decreases, and vice versa [109]. The statistical analysis also shows that this correlation is significant at the 0.05 confidence level ($P < 0.05$) ［Table 4］. In line with the above findings, the impact and pressure of population on land use and land cover (LULC) changes are strongly influenced by population density. Pearson correlation analysis showed that forest cover (P = -0.006) is negatively associated with population pressure on forests [109].

Table 4: Pearson Correlation analysis: Population growth Vs Forest cover change, Yayo coffee forest Biosphere Reserve from 1984 to 2024, Southwest Ethiopia, Supplementary Table (Table S1)

Factors	Population growth	Forest cover change	Year of change
Population growth Pearson Correlation	1	-0.998*	0.999*
Sig. value		0.043	0.020

A study of 148 households in the Yayo Coffee Forest Biosphere Reserve identified key socio-economic factors influencing forest dependence. Multiple linear regression analysis showed that household size ($\beta = -0.32$), income ($\beta = -0.0011$), and education ($\beta = -0.21$) were all significantly and negatively associated with forest dependency ($p < 0.001$ for all). Larger families, higher income, and better education were linked to reduced reliance on forest resources, likely due to diversified livelihoods and greater access to alternatives. These findings support previous research on the socio-economic drivers of forest use and conservation [110]. The inverse relationship between education and forest dependency ($\beta = -0.21$, $p < 0.001$), and its positive contribution to conservation behavior ($\beta = 0.31$, $p < 0.001$), the environmental awareness is enhanced and pressure on forest resources is reduced through education [111]. Conservation behavior was analyzed using

a logistic regression model, with a binary variable indicating whether households participated in conservation. Education had a positive and significant effect ($\beta = 0.31$, $p < 0.001$), suggesting that higher education promotes environmental awareness. Income also showed a positive association ($\beta = 0.0017$, $p = 0.005$), implying that wealthier households are more likely to engage in sustainable practices. Similarly, household size was positively linked to conservation behavior ($\beta = 0.28$, $p = 0.002$), possibly due to greater labor availability for community forest management activities [Table 5].

Table 5: Socio-Economic Determinants of Forest Dependency and Conservation Behavior in Yayo coffee forest Biosphere Reserve, Southwest Ethiopia, 2024, Supplementary Table (Table S2)

Variable	Linear Regression – Dependency on Forest Resources		Logistic Regression – Conservation Behavior	
	Coefficient (A)	*p-value*	Coefficient (B)	*p-value*
Family Size	-0.32	0.000	0.28	0.002
Income Level (ETB)	-0.0011	0.000	0.0017	0.005
Education status (Years)	-0.21	0.000	0.31	0.000

Similarly, income's role in decreasing forest dependency ($\beta = -0.0011$, $p < 0.001$) and increasing conservation participation ($\beta = 0.0017$, $p = 0.005$) aligns with the finding that income diversification enables households to meet their demands without over-exploiting forests [112]. Although it appears contradictory to assume that family size would be positively associated with conservation conduct, this study found that larger families were less dependent on the forest ($\beta = -0.32$, $p < 0.001$) and more involved in conservation activities ($\beta = 0.28$, $p = 0.002$), consistent with those who argued that larger families are capable of rendering more effort and labor towards conservation [113].

4 CONCLUSION AND IMPLICATIONS

The Yayo Coffee Forest Biosphere Reserve, which supports biodiversity conservation and sustainable development, is facing threats from population growth, agricultural encroachment, forest product extraction, and unregulated investments like coal mining. Deforestation is primarily driven by settlement and coffee land clearance, poor local involvement, poverty, and weak institutional coordination. To ensure long-term conservation, community participation, customary institutions, coffee production outside the forest, and impact assessments of extractive activities

are crucial. Good governance, harmonization of customary systems with government conservation policies, and guidelines for resource management and local acceptance are also essential. The government must ensure investment supports local people and does not displace them, ensuring sustainable biosphere use and resilient livelihoods.

Furthermore, the finding suggests the Yayo Coffee Forest Biosphere Reserve is a key example of community-based conservation efforts that contribute to global agricultural climate change resilience. As the world grapples with climate change and biodiversity loss, biosphere reserves like Yayo should be prioritized for sustainable development and climate adaptation. Increasing household income can reduce pressure on forest ecosystems, and alternative income-generating opportunities like eco-tourism, apiculture, coffee certification schemes, and agroforestry-based enterprises can be promoted. Current forest restoration efforts often prioritize ecological goals over socio-economic development, emphasizing the need for holistic approaches that consider education, income, and household structure in forest use and conservation.

ABBREVIATIONS

CBOs: Community Based Organization; **DAs**: Developmental Agents; **EARO**: Ethiopian Agricultural Research Organization; **FAO:** Food and Agricultural Organization; **IUCN**: International Union for Conservation of Nature and Natural Resources; m.a.s.l.: Meter above sea level; **MAB**: Program on Man and the Biosphere; **NGOs:** Non-Governmental Organizations; **SPSS**: Statistical Package for Social Sciences; **STCP**: Sustainable Tree Crops Program; **UNESCO**: United Nations Educational, Scientific and Cultural Organization; and **ZEF**: Zentrum fur Entwicklungsforschung (Center for Development Research).

DATA AVAILABILITY STATEMENT

The original contributions of the study are provided in the article/Supplementary Material; any additional inquiries can be directed to the corresponding author.

SUPPORTING INFORMATION

- Figure S1: Satellite Images Results of Land Use Land Cover in the Yayo coffee forest Biosphere Reserve from 1984 to 2024, Southwest Ethiopia [Hurumu, Doreni and Yayo districts]

- Table S1: Pearson Correlation analysis Data: Population growth Vs Forest cover change, Yayo coffee forest Biosphere Reserve from 1984 to 2024, Southwest Ethiopia
- Table S2: Socio-Economic Determinants Data: Forest Dependency and Conservation Behavior in Yayo coffee forest Biosphere Reserve, Southwest Ethiopia, 2024

AUTHOR CONTRIBUTIONS

FM and AA, and TJ: Conceptualization, methodology, and software. TJ: formal analysis, FM: writing original draft preparation, TJ and FM; writing review and editing. All authors have read and agreed to the published version of the manuscript.

FUNDING

The authors have not declared a specific grant for this research from any funding agency in the public, commercial or not-for-profit sectors.

CONFLICTS OF INTEREST

The authors declare no conflicts of interest.

REFERENCES

1. Bonan, G.B., Forests and climate change: forcings, feedbacks, and the climate benefits of forests. science, 2008. **320**(5882): p. 1444-1449.

2. Canton, H., Food and agriculture organization of the United Nations—FAO, in The Europa directory of international organizations 2021. 2021, Routledge. p. 297-305.

3. Kumar, R., A. Kumar, and P. Saikia, Deforestation and forests degradation impacts on the environment, in Environmental degradation: Challenges and strategies for mitigation. 2022, Springer. p. 19-46.

4. Ngo, T.T.H., et al., Forest-related culture and contribution to sustainable development in the northern mountain region in Vietnam. Forest and Society, 2021. **5**(1): p. 32-47.

5. Odum, E. and G. Barrett, Fundamentals of Ecology.(5thedn). Belmont, Thomson Brooks/Cole, 2005.

6. Inversini, A., et al., The rise of regenerative hospitality. Journal of Tourism Futures, 2024. **10**(1): p. 6-20.

7. Nkonya, E., et al., Economics of land degradation in sub-Saharan Africa. Economics of land degradation and improvement–a global assessment for sustainable development, 2016: p. 215-259.

8. Vlek, P.L., A. Khamzina, and L.D. Tamene, Land degradation and the sustainable development goals: Threats and potential remedies. 2017.

9. Osman, K.T., Soil degradation, conservation and remediation. Vol. 820. 2014: Springer.

10. Sanz, M.J., et al. Sustainable land management contribution to successful land-based climate change adaptation and mitigation: A report of the science-policy interface. in Bonn, Germany: United Nations Convention to Combat Desertification (UNCCD). 2017.

11. Kidane, B., et al., Journal of Agriculture and Food Research.

12. Ramankutty, N., et al., Global land-cover change: Recent progress, remaining challenges. Land-use and land-cover change: local processes and global impacts, 2006: p. 9-39.

13. Kassaye, S.M., et al., Quantifying the climate change impacts on the magnitude and timing of hydrological extremes in the Baro River Basin, Ethiopia. Environmental Systems Research, 2024. **13**(1): p. 1-15.

14. Chofore, A.T., Native tree species based afforestation/reforestation for carbon sequestration: contributions to sustainable development through clean development mechanisms in Ethiopia. 2021: University of South Africa (South Africa).

15. Kovačević, V., et al., 6. Adaptation to climate change in agriculture–status, gaps and recommendations in Serbia. CLIMATE CHANGE ADAPTATION IN AGRICULTURE–STATUS AND PROSPECTS IN WESTERN, 2024: p. 271.

16. Muluneh, T., A. Belayneh, and M. Chimdessa, DIVERSITY AND CONSERVATION STATUS OF NON-TIMBER FOREST PRODUCTS (NTFPS) IN AMIGNA DISTRICT, EAST ARSI ZONE OF OROMIA REGIONAL STATE. 2023, Haramaya University.

17. Prabawani, B., et al., Socioeconomic perspective of agroforestry development in Central Java. Environmental and Sustainability Indicators, 2024. **22**: p. 100354.

18. Tulu, Z.J., Institutions, Incentives and Conflict in Coffee Forest Use and Conservation: the Case of Yayo Forest in Iluu Abba Bora Zone, Southwest Ethiopia. 2010.

19. Gole, T.W. and F. Senbeta, Sustainable management and promotion of forest coffee in Bale, Ethiopia. Bale Eco-Region Sustainable Management Programme SOS Sahel/FARM-Africa, Addis Ababa, 2008.

20. Assen, Y.M., et al., Climate change threats to UNESCO-Designated world heritage sites: empirical evidence from Konso cultural landscape, Ethiopia. Sustainability, 2024. **16**(19): p. 8442.

21. Donoghue, M.J., A phylogenetic perspective on the distribution of plant diversity. Proceedings of the National Academy of Sciences, 2008. **105**(supplement_1): p. 11549-11555.

22. Abera, W., et al., Characterizing and evaluating the impacts of national land restoration initiatives on ecosystem services in Ethiopia. Land Degradation & Development, 2020. **31**(1): p. 37-52.

23. Duguma, M.S., D.H. Feyssa, and L. Biber-Freudenberger, Agricultural biodiversity and ecosystem services of major farming systems: a case study in Yayo Coffee Forest Biosphere Reserve, Southwestern Ethiopia. Agriculture, 2019. **9**(3): p. 48.

24. Bramel, P., et al., Global conservation strategy for coffee genetic resources. Crop Trust and World Coffee Research, 2017. **72**.

25. Madeira, F., et al., The EMBL-EBI Job Dispatcher sequence analysis tools framework in 2024. Nucleic acids research, 2024. **52**(W1): p. W521-W525.

26. Rawal, R., et al., The himalayan biodiversity: richness, representativeness, uniqueness and life-support values. GB Pant Institute of Himalayan Environment and Development (GBPIHED), Kosi-Katarmal, Almora, Uttarakhand, India, 84pp, 2013.

27. Sang, W., Plant diversity patterns and their relationships with soil and climatic factors along an altitudinal gradient in the middle Tianshan Mountain area, Xinjiang, China. Ecological research, 2009. **24**(2): p. 303-314.

28. McGraw, D.M., The CBD-key characteristics and implications for implementation. Rev. Eur. Comp. & Int'l Envtl. L., 2002. **11**: p. 17.

29. Agidew, A.-m.A. and K. Singh, The implications of land use and land cover changes for rural household food insecurity in the Northeastern highlands of Ethiopia: the case of the Teleyayen sub-watershed. Agriculture & Food Security, 2017. **6**(1): p. 56.

30. Hassen, A., et al., Local People's perception of forest ecosystem services, traditional conservation, and management approaches in North Wollo, Ethiopia. Journal of Environmental Management, 2023. **330**: p. 117118.

31. Kalele, D.N., et al., Climate change impacts and relevance of smallholder farmers' response in arid and semi-arid lands in Kenya. Scientific African, 2021. **12**: p. e00814.

32. Kuma, H.G., F.F. Feyessa, and T.A. Demissie, Land-use/land-cover changes and implications in Southern Ethiopia: evidence from remote sensing and informants. Heliyon, 2022. **8**(3).

33. Gole, T.W., et al., Floristic composition and environmental factors characterizing coffee forests in southwest Ethiopia. Forest Ecology and Management, 2008. **255**(7): p. 2138-2150.

34. Tadesse, B., et al., Foot and mouth disease outbreak investigation and estimation of its economic impact in selected districts in northwest Ethiopia. Veterinary medicine and science, 2020. **6**(1): p. 122-132.

35. Tafere, H.B. and S.L. Woreta, Tourism Potential and Its Development Challenges in Jabi Tehnan Woreda, West Gojjam Zone, Ethiopia.

36. Walle, Y. and D. Nayak, Factors determining the participation of natural resource cooperative members in forest management: A study of dry forest area in Ethiopia. Trees, Forests and People, 2022. **8**: p. 100241.

37. Mengistu, D.A. and A.T. Salami, Application of remote sensing and GIS inland use/land cover mapping and change detection in a part of south western Nigeria. African Journal of Environmental Science and Technology, 2007. **1**(5): p. 99-109.

38. Yauch, C.A. and H.J. Steudel, Complementary use of qualitative and quantitative cultural assessment methods. Organizational research methods, 2003. **6**(4): p. 465-481.

39. Getahun, D. and E.T. Keno, Attitudes and perceptions of the local community towards Yayo coffee forest biosphere reserve, ilu abba bora zone of Oromia national regional state. Ethiopian Journal of Science and Sustainable Development, 2019. **6**(1): p. 79-90.

40. Etana, D., et al., Climate change, in-situ adaptation, and migration decisions of smallholder farmers in central Ethiopia. Migration and Development, 2022. **11**(3): p. 737-761.

41. Newing, H., et al., Conducting research in conservation. Vol. 775. 2011: Routledge Oxfordshire.

42. Bernard, H.R., Research methods in anthropology: Qualitative and quantitative approaches. 2017: Bloomsbury Publishing PLC.

43. Woldie, B.A. and S.A. Tadesse, Views and attitudes of local people towards community versus state forest governance in Tehulederi District, South Wollo, Ethiopia. Ecological Processes, 2019. **8**(1): p. 4.

44. Krueger, R.A. and M.A. Casey, Focus group interviewing. Handbook of practical program evaluation, 2015: p. 506-534.

45. Bazeley, K. and A. Hayton, Practical cattle farming. 2013: Crowood.

46. Adhikari, B., S. Di Falco, and J.C. Lovett, Household characteristics and forest dependency: evidence from common property forest management in Nepal. Ecological economics, 2004. **48**(2): p. 245-257.

47. Benjamin, D.J., et al., Redefine statistical significance. Nature human behaviour, 2018. **2**(1): p. 6-10.

48. Zhang, Z., Residuals and regression diagnostics: focusing on logistic regression. Annals of translational medicine, 2016. **4**(10): p. 195.

49. Braun, V. and V. Clarke, Using thematic analysis in psychology. Qualitative research in psychology, 2006. **3**(2): p. 77-101.

50. Rahman, M.H. and K. Alam, Forest dependent indigenous communities' perception and adaptation to climate change through local knowledge in the protected area—A Bangladesh case study. Climate, 2016. **4**(1): p. 12.

51. Raimondo, E. and K.E. Newcomer, Mixed-methods inquiry in public administration: The interaction of theory, methodology, and praxis. Review of public personnel administration, 2017. **37**(2): p. 183-201.

52. Snyder, L.R., J.J. Kirkland, and J.L. Glajch, Practical HPLC method development. 2012: John Wiley & Sons.

53. Faaz, M. and Z.N. Khan, A study of academic achievement of upper primary school students in relation to their socio-economic status. Asian Journal of Research in Social Sciences and Humanities, 2017. **7**(6): p. 121-127.

54. Cerqua, A., C. Gauthier, and M. Dembélé, Education policy, teacher education, and pedagogy: A case study of UNESCO. Annual Review of Comparative and International Education 2014, 2014: p. 235-266.

55. Smith-Greenaway, E., Maternal reading skills and child mortality in Nigeria: a reassessment of why education matters. Demography, 2013. **50**(5): p. 1551-1561.

56. Alderman, H. and J.R. Behrman, Reducing the incidence of low birth weight in low-income countries has substantial economic benefits. The World Bank Research Observer, 2006. **21**(1): p. 25-48.

57. Fetensa, A.D., M.S. Dalu, and T. Bedada, Economic contribution of forest resource at household level in the bale mountains eco-region redd+ project, Southern Ethiopia. Journal of Resources Development and Management, 2019. **53**: p. 7-27.

58. Meleaku, T., PRODUCTION EFFICIENCY AND MARKET SUPPLY OF SORGHUM: THE CASE OF SMALLHOLDER FARMERS IN KAFTA-HUMERA DISTRICT OF TIGRAY, ETHIOPIA. 2018, Haramaya university.

59. Bishu, K.G., Risk management and the potential of cattle insurance in Tigray, Northern Ethiopia. 2014.

60. Hein, L. and F. Gatzweiler, The economic value of coffee (Coffea arabica) genetic resources. Ecological economics, 2006. **60**(1): p. 176-185.

61. Stellmacher, T. and P.P. Mollinga, The institutional sphere of coffee forest management in Ethiopia: local level findings from Koma forest, Kaffa zone. International journal of social forestry, 2009. **2**(1): p. 43-66.

62. Snyder, T.D. and S.A. Dillow, Digest of Education Statistics, 2011. NCES 2012-001. National Center for Education Statistics, 2012.

63. Lemenih, M. and H. Kassa, Re-greening Ethiopia: history, challenges and lessons. Forests, 2014. **5**(7): p. 1717-1730.

64. Gebremariam, A. and H. Gebremariam, Contraceptive use among lactating women in Ganta-Afeshum District, Eastern Tigray, Northern Ethiopia, 2015: a cross sectional study. BMC pregnancy and childbirth, 2017. **17**(1): p. 421.

65. Ojulu, O.M., Large-scale land acquisitions and minorities/indigenous peoples' rights under ethnic federalism in Ethiopia. A Case Study of Gambella Regional State. 2014.

66. Daba, A., et al., Weed species composition and abundance in the main coffee production systems and regions of Ethiopia. South African Journal of Plant and Soil, 2022. **39**(1): p. 41-55.

67. Tura, N., et al., Unlocking circular business: A framework of barriers and drivers. Journal of cleaner production, 2019. **212**: p. 90-98.

68. Gufu, G.D., et al., Do invasive exotic and native freshwater plant species respond similarly to low additional nitrate doses? Aquatic Botany, 2018. **151**: p. 1-8.

69. Terfassa Fida, G., B.N. Baatuuwie, and H. Issifu, Dynamics of land use/cover change and its drivers during 1992–2022 in Yayo Coffee Forest Biosphere Reserve, Southwestern Ethiopia. Sustainable Environment, 2024. **10**(1): p. 2374119.

70. Ostrom, E., Governing the commons: The evolution of institutions for collective action. 1990: Cambridge university press.

71. Cleaver, D.J., Z. Wang, and I. Gursul, Bifurcating flows of plunging aerofoils at high Strouhal numbers. Journal of Fluid Mechanics, 2012. **708**: p. 349-376.

72. Agrawal, A., Common property institutions and sustainable governance of resources. World development, 2001. **29**(10): p. 1649-1672.

73. Tesema, S.F., Impact of technological change on household production and food security in smallholders agriculture: the case of wheat-tef based farming systems in the central highlands of Ethopia. 2006: Cuvillier Verlag.

74. Tulu, A., The Practice and Challenges of School-Based Teachers' Continuous Professional Development: A Case of Government Secondary Schools of Hawassa City in Ethiopia. Educational Research and Reviews, 2019. **14**(1): p. 33-43.

75. Derso, W.G. and B. Gebremichael, The leasehold system and drivers of informal land transactions in bahir dar city, Ethiopia. Journal of Eastern African Studies, 2022. **16**(3): p. 415-433.

76. Cleaver, F., Development through bricolage: Rethinking institutions for natural resource management. 2017: Routledge.

77. Yusuf, A., THE DETERMINANTS OF RURAL HOUSEHOLDS FOOD SECURITY AND COPING STRATEGIES: THE CASE OF META DISTRICT, EAST HARARGHE ZONE OF OROMIA NATIONAL REGIONAL STATE OF ETHIOPIA. 2017, Haramaya university.

78. Eshetu, A.A., Forest resource management systems in Ethiopia: Historical perspective. International Journal of Biodiversity and Conservation, 2014. **6**(2): p. 121-131.

79. Asfaw, A. and L. Etefa, The contribution of non-timber forest products to the rural livelihood: The case of yayo district, illu ababora zone, oromia regional state, western Ethiopia. International Journal of Applied Agricultural Research, 2017. **12**(2): p. 157-169.

80. Moloro, H.B. and T. Abebe, Review on socio economic and ecological role of non timber forest products in South Western Ethiopia. Ethiopian Journal of Economics, 2022. **3**(2): p. 46-65.

81. Heron, R. and W. Juju, The marina-sustainable solutions for a profitable business. 2012: Lulu. com.

82. Mulugeta, S.B., Drought, vulnerability and adaptation: risk of food and livelihoods insecurity for pastoralists and agro-pastoralists in Borana zone, southern Ethiopia. 2023, University of Nairobi.

83. Birhanu, A.G., A. Gambardella, and G. Valentini, Bribery and investment: Firm-level evidence from A frica and L atin A merica. Strategic Management Journal, 2016. **37**(9): p. 1865-1877.

84. Seto, K.C., B. Güneralp, and L.R. Hutyra, Global forecasts of urban expansion to 2030 and direct impacts on biodiversity and carbon pools. Proceedings of the National Academy of Sciences, 2012. **109**(40): p. 16083-16088.

85. Agrawal, A. and C.C. Gibson, Enchantment and disenchantment: the role of community in natural resource conservation. World development, 1999. **27**(4): p. 629-649.

86. Alemu, B., et al., Land use and land cover changes and associated driving forces in north western lowlands of Ethiopia. International research journal of agricultural science and soil science, 2015. **5**(1): p. 28-44.

87. Herforth, A., et al., Cost and affordability of healthy diets across and within countries: Background paper for The State of Food Security and Nutrition in the World 2020. FAO Agricultural Development Economics Technical Study No. 9. Vol. 9. 2020: Food & Agriculture Org.

88. Teketay, D., et al., Enhancement of diversity, stand structure and regeneration of woody species through area exclosure: the case of a mopane woodland in northern Botswana. Ecological Processes, 2018. **7**(1): p. 5.

89. Wiersum, K.F. and T.B. Endalamaw, Governing forests for provisioning services: the example of honey production in southwest Ethiopia, in Governing the Provision of Ecosystem Services. 2012, Springer. p. 303-318.

90. Wiersum, K. and T. Endalamaw, Governing the provision of ecosystem services. Studies in ecological economics, vol 4. 2013.

91. Menashe-Oren, A. and D.A. Sánchez-Páez, Male fertility and internal migration in rural and urban Sub-Saharan Africa. European Journal of Population, 2023. **39**(1): p. 10.

92. Wassie, S.B., Natural resource degradation tendencies in Ethiopia: a review. Environmental systems research, 2020. **9**(1): p. 1-29.

93. Groth, O., The Great Remobilization: Strategies and Designs for a Smarter Global Future. 2023: MIT Press.

94. Woube, M., Effects of resettlement schemes on the biophysical and human environments: The case of the Gambela Region, Ethiopia. 2005: Universal-Publishers.

95. Morrow, N., Autochthonous and introduced stores of biomass value: Measuring resilience outcomes of enset and eucalyptus as green assets in three representative smallholder farm systems of Ethiopia. 2021, University of Maryland, College Park.

96. Molla, T., et al., Diversity of woody species in traditional agroforestry practices in Wondo district, south-central Ethiopia. Heliyon, 2023. **9**(2).

97. Descheemaeker, K., et al., Runoff on slopes with restoring vegetation: a case study from the Tigray highlands, Ethiopia. Journal of hydrology, 2006. **331**(1-2): p. 219-241.

98. Wolde, M. and E. Veldkamp. Impacts of land use changes on soil nutrients and erosion in Tigray, Ethiopia. in Conference on International Agricultural Research for Development, Deutscher Tropentag. 2005.

99. Worku Annota, C., S. Asfaw, and S. Tekaligni, DETERMINANTS OF SMALLHOLDER FARMERS'CHOICE OF ADAPTATION STRATEGIES TO THE EFFECTS OF CLIMATE CHANGE: THE CASE OF CHIRO DISTRICT, WEST HARARGHE ZONE, OROMIA REGION, ETHIOPIA. 2021, Haramaya University.

100. Amsalu, A. and J. De Graaff, Determinants of adoption and continued use of stone terraces for soil and water conservation in an Ethiopian highland watershed. Ecological economics, 2007. **61**(2-3): p. 294-302.

101. Nurrochmat, D.R., et al., Transformation of agro-forest management policy under the dynamic circumstances of a two-decade regional autonomy in Indonesia. Forests, 2021. **12**(4): p. 419.

102. Melese, S.M., Effect of land use land cover changes on the forest resources of Ethiopia. International Journal of Natural Resource Ecology and Management, 2016. **1**(2): p. 51.

103. Guala, A., et al., Wall shear stress predicts aortic dilation in patients with bicuspid aortic valve. Cardiovascular Imaging, 2022. **15**(1): p. 46-56.

104. Berhanu, A.A., Vulnerabilities, Response Strategies, and Resilience of Smallholder Farmers to Climate Change in the Upper Blue Nile Basin of Ethiopia. 2025.

105. Gebretsadik, T., Causes for biodiversity loss in Ethiopia: A review from conservation perspective. Journal of Natural Sciences Research, 2016. **6**(11): p. 32-40.

106. Lung, T. and G. Schaab, A comparative assessment of land cover dynamics of three protected forest areas in tropical eastern Africa. Environmental monitoring and assessment, 2010. **161**(1): p. 531-548.

107. Yadeta, T., et al., Recent resettlement programs, as drivers for Afromontane forest loss in the Hawa-Galan district of Ethiopia. Cogent Social Sciences, 2022. **8**(1): p. 2088462.

108. Mohammed, A., S. Bahmud, and A. Abdulahi, The status of forest development in Ethiopia: challenges and opportunities: a review paper. 2023.

109. Belayneh, Y., et al., Forest cover change and its driving forces in Fagita Lekoma District, Ethiopia. Journal of forestry research, 2020. **31**(5): p. 1567-1582.

110. Babulo, B., et al., Household livelihood strategies and forest dependence in the highlands of Tigray, Northern Ethiopia. Agricultural systems, 2008. **98**(2): p. 147-155.

111. Tadesse, W., Towards the sustainable conservation, management and utilization of dry forests in Ethiopia. A review, 2021: p. 38.

112. Ango, T.G., REGIONAL AND LOCAL DEVELOPMENT STUDIES. 2005.

113. Seboka, A.D., et al., Potentials and prospects of solid biowaste resources for biofuel production in Ethiopia: a systematic review of the evidence. Biomass Conversion and Biorefinery, 2024. **14**(24): p. 30929-30960.

Click here to access/download
Supporting Information
Supplimentary Informations.docx

IMPACT OF POPULATION PRESSURE ON FOREST RESOURCES DEPLETION IN YAYO COFFEE FOREST BIOSPHERE RESERVE, SOUTHWEST ETHIOPIA

Fikru Mosisa [1,*], Adanech Asfaw [2], and Tefera Jogora [2]

[1]*Department of Biology, College of Natural Science, Mattu University, Mattu, Ethiopia*

[2] *Department of Forestry, College of Agriculture and Forestry, Mattu University, Mattu, Ethiopia*

[*]*Corresponding Author: Email:* ***fikrumosisa@gmail.com****, phone number: +251917851910, P. O. Box: 381*

ABSTRACT

Forests regulate the climate and support rich biodiversity, but growing population pressure threatens these vital functions through deforestation and habitat loss. The research was carried out in the Yayo Coffee Forest Biosphere Reserve, located in the Oromia region of southwest Ethiopia. Data were gathered from key informants, kebele leaders, development agents, selected households, focus group discussions, and field observations. Land use and land cover change data were obtained from satellite imagery spanning 1984 to 2024. Population growth data were sourced from the Jimma branch of the Ethiopian Central Statistical Agency. Additionally, field visits included direct observations of the forest's condition. A total of 148 participants responded to both structured and semi-structured questionnaires designed to address the study's objectives. The communities in the study area do not rely on a single livelihood strategy but instead engage in various activities such as crop cultivation, forestry, livestock rearing, and off-farm work, with varying levels of dependence on each. Among the forest products collected, coffee, firewood, honey, and wild spices were the most common. The main factors contributing to forest degradation were ranked as agricultural expansion (23.13%), lack of local participation (21.95%), timber extraction (15.27%), urbanization (14%), effects of climate change (13.46%) and investment (12.2%) in decreasing order. Satellite image analysis revealed a negative correlation between population growth and forest cover, with a significant difference at $P<0.05$. The study highlights the impact of population pressure on forest resource degradation in the Yayo Coffee Forest Biosphere Reserve. Therefore, key players in forest management and biosphere reserve

implementation, policy makers, and community members should focus on risk mitigation and on sustainable management, conservation, and utilization of the biosphere reserve.

***Key words**: degradation; forest management; habitat loss; population pressure; land use change*

1 INTRODUCTION

Forests play a crucial role in keeping life on earth intact as the world's lungs, taking in carbon dioxide and expelling oxygen, which keeps the climate stable [1, 2]. They shelter incredible diversity of flora and fauna, with over 80% of land-based biodiversity in the world [2]. Besides keeping ecosystems intact, forests are needed for water purification, preventing soil erosion, and flood regulation. For millions, especially rural and indigenous populations, forests are a critical source of livelihood, food, fuel, medicine, and much more [3]. Forests hold cultural, spiritual, and recreational importance for communities worldwide [4]. The concept of biological carrying capacity, which defines the maximum population that an environment can sustain on a sustainable basis, is widely used to study the impact of demographic change on natural resources [5]. This capacity depends on a range of factors including economic development, socio-political processes, technology, trade, and patterns of consumption [6].

Sub-Saharan Africa land degradation in most areas is a result of a combination of direct and indirect human intervention [7]. Direct causes includes deforestation, overgrazing, unsustainable timber extraction, shifting cultivation and mismanagement of water and soil resources [3, 8, 9] These are compounded by inadequate conservation measures, poorly chosen crop rotations, marginal land use for agriculture, improper fertilizer application, inefficient irrigation, and excessive groundwater abstraction. [10]. Indirect causes include high population growth rates, restricted availability of land for cultivation, insecure tenure regimes, poverty, and pervasiveness of economic hardship [11].

Ethiopia is a limited exception where deforestation, after acute growth in population with low socio-economic development, has led to serious environmental degradation [9, 11]. Forest cover that once covered approximately 40% of land area in the early 1900s has drastically declined to less than 2.2% in recent decades [11 - 13]. The Yayo Coffee Forest Biosphere Reserve plays a critical role in carbon sequestration, supports international conservation of biodiversity, and aids global climate and sustainability agendas such as the SDGs and the Paris Agreement

[14]. Reforestation efforts under the Green Legacy Initiative have nonetheless been an influence that has witnessed modest forest cover increases, rising to approximately 6.4% from 17.2% in 2019 to 23.6% in 2023 [15]. Despite ongoing conservation efforts within the Yayo Coffee Forest Biosphere Reserve, there remain current challenges such as low community engagement and inadequate linkage of local livelihoods [16]. The majority of restoration initiatives aim at ecological goals while neglecting socio-economic needs [17]. Therefore, this research aims to overcome the impacts of population pressure on forest resource depletion in the Yayo coffee forest biosphere reserve, Ilubabor Zone, Oromia Regional State, Southwest Ethiopia.

2 METHODS AND MATERIALS

2.1 Study area description

The Yayo Coffee Forest Biosphere Reserve is located in the Ilu Abba Bora Zone of the Oromia Regional State in southwestern Ethiopia. It is recognized as the center of origin for Coffea arabica, the world's most widely consumed coffee, and Yayo is the largest and most significant forest for conserving wild coffee populations globally [18]. The reserve plays a crucial role in preserving both natural and cultural landscapes [19]. Situated in the southwestern part of the Oromia region, the Yayo Biosphere Reserve includes the Woredas of Hurumu, Yayo, Chora, Nopha, Alge Sachi, and Doreni, covering coordinates from 8°0'42" to 8°44'23" N and 35°20'31" to 36°18'20" E [Fig. 1].

The district's elevation ranges from 1,139.2 to 2,581.9 meters above sea level, with the lowest point located at Gaba River (1,100 meters) and the highest point at the summit of Sayi Mountain (2,581 meters) in Keresi. The area experiences a hot and humid climate, with an average annual temperature of around 23°C, ranging from a mean minimum of 18.59 ˚C to a mean maximum of 27.88 ˚C [20]. The varied physical conditions and altitudinal differences contribute to a rich diversity of climate, soil, and vegetation, fostering the development of numerous plant species with a high level of diversity [21]. The rainfall pattern of the districts varies annually from 1,191.6 to 1,960.7 mm, showing variations from year to year. It is a unimodal type of rainfall that increases from May to October and declines in November [22].

The study focuses on the Hurumu, Yayo, and Doreni districts located in the Yayo Coffee Forest Biosphere Reserve in southwestern Ethiopia, which is famous for its ecological importance and genetic diversity of wild Coffea arabica [23]. The biosphere reserve has a total surface area of 167,021 hectares and consists of three zones: a core zone (27,733 ha), a buffer zone (21,552 ha), and a transition zone (117,736 ha), balancing conservation and sustainable development [24]. Land in the study area comprises 3.5% highland (5,750.4 ha), 85% temperate zone (138,465.85 ha), and 11.47% lowland (18,684.75 ha), reflecting its heterogeneity and altitudinal gradients [25]. This diversity accounts for much of the biodiversity of the region and explains its status as a key location for environmental studies and resource management [26].

The diverse climatic conditions and habitats in these districts have contributed to a high level of species diversity in both plants and animals [27]. This biodiversity richness is one of the reasons why Ethiopia is considered one of the 20 most biodiverse countries in the world [28].

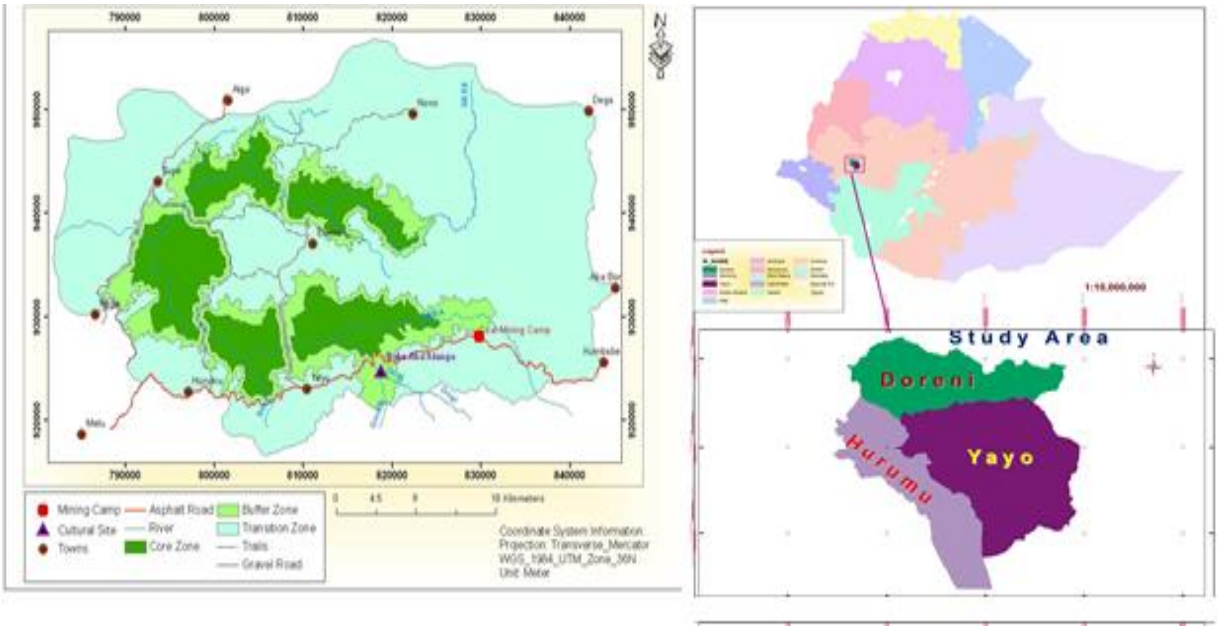

Fig. 1: Maps of the study area (Yayo coffee forest Biosphere Reserve, Southwest Ethiopia, 2024)

2.2 Study Design

This research employs a mixed-methods research design, integrating both quantitative and qualitative approaches to comprehensively investigate the impacts of population dynamics on the

Yayo Biosphere Reserve. The design is structured to systematically address the following objectives: assessing population growth, evaluating forest cover change, examining conservation practices, understanding community resource relationships, identifying stakeholder roles, and proposing sustainable management strategies.

2.3 Data collection

Following ethical approval, the study was conducted in several data collection waves between May 2024 and January 2025. The data for this study includes a sample household survey and land use/land cover change data. The research approach involves a consultative and interactive process, engaging respondents who are willing to provide essential information [29]. Interviews were conducted to collect data on the role of local communities in forest conservation, the impacts of human activities on the forest, and perceptions toward conservation efforts, as well as the challenges and consequences associated with these activities [30]. To collect data, formal survey questionnaires were used to gather quantitative information from selected households [31]. Focus group discussions and interviews were conducted to collect primarily qualitative data. Satellite imagery was utilized to generate land use land cover change data [32].

2.4 Population

The population of interest for this study comprised rural households and key stakeholders residing in and around the Yayo Coffee Forest Biosphere Reserve, specifically within the selected woredas of Yayo, Hurumu, and Doreni. During the study time, there were a total of 91,694 households; 89,039 were males and 2,659 were females, with a total population of 458,472 individuals. These communities were chosen due to their direct dependence on forest resources for their livelihoods and their proximity to the core and buffer zones of the biosphere reserve [33, 34].

The sampling frame included residents from nine kebeles, three from each of the selected woredas, identified based on geographic location, accessibility, and relevance to forest conservation activities [35]. Households were selected using purposive sampling techniques to ensure the inclusion of individuals with lived experience in forest resource use and conservation [36]. Criteria such as length of residence in the area, proximity to the forest, and knowledge of local forest management practices were considered during the selection process [37].

2.5 Sample Size and Sampling Techniques

To ensure a comprehensive understanding of the study area, a combination of preliminary assessments and quantitative and qualitative surveys was employed [38]. An initial exploratory survey was conducted to gain an overview of the spatial distribution of the Yayo Coffee Forest Biosphere Reserve and to facilitate the selection of appropriate study sites [23]. The Yayo Coffee Forest Biosphere Reserve encompasses six woredas of the Ilubabor and Bunobedele zones, namely Yayo, Hurumu, Chora, Bilo Nopha, Alge Sachi, and Doreni. From the foregoing, for the present study, Yayo, Hurumu, and Doreni were utilized as representative samples [39]. Among these sampled woredas, three rural kebeles were selected randomly, and nine kebeles were ultimately selected in the end: Haro, Gaba, and Wangegne of Hurumu woreda; Waboo, Geci, and Wixete of Yayo woreda; and Bocho, Badesa, and Henna of Doreni woreda. These kebeles were chosen on purpose since they are nearest to the forest zone. Respondents in these kebeles were chosen according to purposive criteria, including location near the forest, residence in the biosphere reserve for years, and knowledge on forest resource use and conservation methods [39].

To gather quantitative information, a systematic household survey was conducted with 148 respondents from the chosen kebeles. The survey collected baseline data on household characteristics, land use, livelihood strategies, and perceptions towards forest conservation [40, 41]. At the same time, qualitative information was gathered through key informant interviews and FGDs. 18 key informants, two from each kebele, were interviewed. These informants included village officials, kebele administration, development agents (DAs), and the relevant subject-matter experts. Their feedback provided contextual depth to the quantitative data and enriched the analysis as a whole [42]. Three FGDs were also organized to garner community-level perspectives regarding forest management and socio-economic issues [43]. Three FGDs were made, each containing seven members, one from each woreda, resulting in a total of 21 participants. These interviews enriched the qualitative data through the injection of locally grounded knowledge and lived experience [44].

2.6 Data Analysis

The quantitative data collected through household surveys were first cleaned, validated, and coded in Microsoft Excel to promote consistency and accuracy [45]. The cleaned data were then exported into SPSS version 20 for analysis. Descriptive statistics were used in summarizing key variables such as household characteristics, forest resource use, and conservation knowledge [46].

To analyze causal relationships, multiple linear regression and logistic regression tests were used. These methods allowed the assessment of the impact of independent variables, i.e., household size, income level, and education, on dependent variables like dependency on forest resources and conservation behavior [47]. The validity of the regression models was checked by using diagnostic tests [48].

For the qualitative data collected through key informant interviews and focus group discussions, thematic analysis was applied [49]. The data were coded, transcribed, and categorized into local perceptions, emerging themes, traditional knowledge, forest governance, and livelihood strategies. Representative statements of the participants were utilized to elaborate on these themes [50]. Through this mixed-methods approach with regression-based quantitative analysis complemented by rich qualitative feedback, there was a guaranteed holistic understanding of the drivers, effects, and community perspectives about the use and preservation of forest resources [51].

2.7 Ethical Considerations

The study was conducted in accordance with ethical standards, with ethical clearance granted by the Institutional Research and Ethics Review of Mattu University. Participants were informed about the study's purpose, procedures, and voluntary nature, and their identities were kept confidential. Participation was voluntary, with the option to withdraw at any point without consequence. No financial or material incentives were provided to influence participation. The research tools and interview protocols were culturally sensitive and respectful of local customs and norms. Permission to conduct the study was obtained from local authorities and community leaders, particularly in selected kebele and woreda. Data was securely handled and stored to protect participants' privacy, and access was restricted to the research team only.

3 RESULTS AND DISCUSSION

3.1 Socio-economic characteristics

The majority of the households, 89,039 (97.1%), were male-headed, while only 2,659 (2.9%) were female-headed. The ages of the respondents ranged from 20 to 80 years, with a mean age of 48 years. The family size varied between 1 and 12 members, with an average of 5.18, which is consistent with the national average family size. Regarding education, 15.9% of the respondents

were illiterate (unable to read or write), 30.4% had attended grades 1-5, 42% had completed grades 6-12, and 11.6% had completed secondary. On income, 39.8% of the households were low-income, 35.1% middle-income, and only 10.8% high-income. In terms of household size, the largest share (55.4%) included 4–5 members, then 23.6% included 6–7 members, and 12.8% included over 7 [Table 1]. Similarly, the family's socioeconomic status includes the household income, earners' education and occupation, as well as combined income when their own attributes are assessed [52]. In fact, the socio-economic status can be measured in a number of different ways, and most commonly, it is measured by education, occupation, and income [53]. Family illiteracy drastically restricts economic opportunities via limited availability of well-paying employment, resulting in poverty; affects the health services; and is unlikely to possess the capacity to aid their education [54- 56].

Table 1: Socio-demographic and economic characteristics of the participants among households in Yayo coffee forest Biosphere Reserve, Southwest Ethiopia, 2024.

Variables	Category	Frequency	Percent (%)
Sex	Male	144	97.2
	Female	4	2.8
	Total	148	100.0
Age	20-30	4	2.9
	31-40	58	39.1
	41-50	34	23.2
	51-60	22	14.5
	>61	30	20.3
	Total	148	100.0
Educational status	Illiterate (no formal education)	24	15.9
	Grade 1-5	45	30.4
	Grade 6-12	62	42.0
	Diploma & above	17	11.6
	Total	148	100.0
Income level	No income	21	14.2
	Low income	59	39.8
	Middle income	52	35.1
	High income	16	10.8
	Total	148	100
Family size	≤ 3	12	8.0
	4–5	82	55.4
	6 –7	35	23.6
	> 7	19	12.8
	Total	148	100.0

3.2 Livelihood strategies

In this study, among the sample households that participated in the survey, 37.7% of respondents indicated that their income came from crop production, 22.7% from forest products, and 18.7% from livestock production. Additionally, 27.5% reported that their income sources were crop production and forest products. The remaining 8.7% of respondents indicated that they relied on all types of household income-generating activities (Fig.2). Against the above result, 73% of forest dwellers depend on these products as a source of revenue in the Bale Zone, southern Ethiopia [57]. The value of income sources like forest products, crop production, livestock production, and off-farm activities depends on each other [58]. Research conducted in the adjacent district of Yayo, included under one National Forest Priority Area

(NFPA) with Gabba-Dogi, i.e., Yayo NFPA [59], revealed that 92.6 percent of the population in the study area have coffee in the forest, from which 57.3 kg of honey on average is harvested per household per year [59, 60].

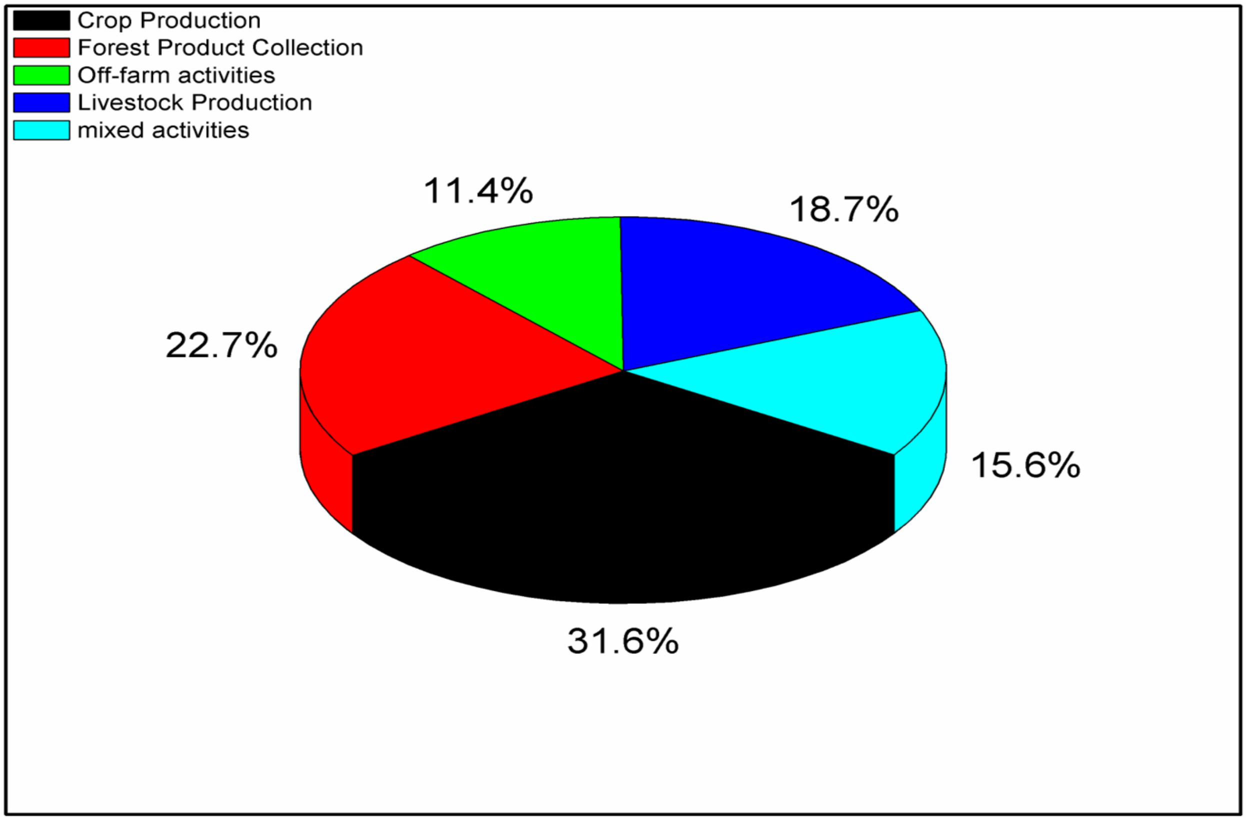

Fig. 2: Livelihood strategies among study participants in Yayo coffee forest Biosphere Reserve, Southwest Ethiopia, 2024

3.3 Formal Institutions in forest management

Based on the information gathered from interviews and focus group discussions, various institutions at the federal, regional, and local levels were identified, along with their interlinkages concerning the management of the coffee forest resource under investigation[61].

Changes in the institutional structure of the Ministry of Agriculture (MoA) since the early 1990s have failed to establish a dedicated government body for the management, conservation, and sustainable use of coffee forests [18]. This gap has contributed, to some extent, to the increasing lack of effective institutions for the sustainable management of coffee forests. Federal institutions,

mainly under the Institution for Biodiversity Conservation (IBC), provide technical support for Yayo Coffee Forest Biosphere Reserve conservation [62].

At the regional level, two institutions with potential connections to the coffee forest are examined: the Oromia Forest and Wildlife Enterprise Supervising Agency and the regional agriculture and rural development bureaus established in 1999 under proclamation 90/1999 as parts of decentralization [52]. The primary issues identified within these organizations include a lack of technical and direct focus on forest coffee biodiversity conservation, insufficient budget and technical personnel at the agricultural and rural development bureaus, inadequate decentralization of the budget, and limited community involvement in planning and execution at the Forest Enterprise Supervising Agency [63]. Another gap noted in the State Forest Enterprise is the absence of incentives to motivate local communities to conserve specific forests [64]. The study confirms that local indigenous institutions have been gradually marginalized due to the increasing control of the state, which enforced formal institutions under various regimes in the past [65].

At the local level, the institutions involved in the use, conservation, and management of the coffee forest include the Yayo Coffee Forest Biosphere Reserve Conservation Project, the district administration (comprising the kebele and development team), and the district [66].

3.4 Informal institutions contributing for forest management.

The study examines various informal institutions in terms of their structure and role in the livelihoods of the community in the study area. These institutions are categorized into four groups, with particular emphasis placed on two clusters [67]. The territorial-based administrative indigenous/customary institutions are further divided into four groups: Tuullaa, Xuxee, Shane, and Jaarsa Biyya, along with Muchoo. The study conducted by Tulu and Getahun revealed the same result with current study. The first cluster consists of territorial-based administrative indigenous/customary institutions, while the second includes a variety of self-help work organizations [68].

Shane, Xuxee, and Tuullaa make two important contributions to the management of coffee forests. A fundamental role in bringing local governance systems into alignment is played by Tuullaa, which organizes, leads, and enforces the work, norms, and regulations of various local customary institutions and self-help labor associations.By controlling coffee harvesting and enforcing customary regulations to stop overexploitation and resource degradation, for example, these organizations manage coffee forests both directly and indirectly [69]. This finding aligns with the broader literature on common-pool resource management, which emphasizes that strong social norms, particularly those founded on reciprocity and generalized trust, are instrumental for the sustainable management

and conservation of collective natural resources. The result highlights the central role of Tuullaa as a culturally grounded institution that governs not only the social and economic existence of the community in the coffee forest zone but also exercises a significant influence over governing the environment [70].

The ability to govern conduct among community members and facilitate the crafting and enforcement of local rules situates it as a significant mechanism for maintaining collective action for sustainable forest use. This is in harmony with common-pool resource governance theory, which praises the effectiveness of locally crafted institutions in shared ecosystem management [71].

Jaarsa Biyya and Muchoo, as customary Oromo institutions, play a crucial role in the sustainable management of the coffee forest and other natural resources by being local enforcers of customary law and mediators in resource disputes. Their authority, which has its foundation in respect by the community, allows them to enforce existing rules and, if supported by the locals, create, and implement new rules. This aligns with the contention that locally embedded institutions are more effective and legitimate in the governance of resources than externally introduced formal institutions [72]. Similarly, it contends for the importance of facilitating resource users to participate in rule-making, a function these institutions are strategically positioned to undertake [73]. This is also supported by who adds that local self-help associations among the Oromo have traditionally worked to improve food security and economic resilience [74 - 75]. Interpreted through the theory of institutional bricolage, Jaarsa Biyya and Muchoo demonstrate how traditional authority can be reshaped to contemporary challenges, blending cultural practice with evolving governance imperatives for promoting sustainability [76].

3.5 Conservation policies and its drawbacks

As indicated by various informants in the coffee forest study area, different forms of ownership rights existed prior to the demarcation process. The publicly owned and strictly conserved portion of 50,000 hectares of the Yayo Coffee Forest Biosphere Reserve is the most significant. The remaining 117,000 hectares fall under a mix of community, cooperative, and private regimes, which work to strengthen traditional forest coffee production. Such sites support over 150,000 people, with 40–50% of the reserve in mixed regimes [77]. Federal Forest Development, Conservation, and Utilization Proclamation no. (5, 6) has a sure implication for natural resource

management, even though in the practical implementation they have faced their own limitation [78].

3.6 The contributions of the Biosphere reserve to the local communities

In this study, the collection of forest products was identified as the primary source of household income. Approximately 43.9% of respondents stated that they are highly dependent on forest products to support their households. Additionally, 37.8% of the households surveyed indicated that collecting forest products is their top priority for sustaining their livelihoods, While 16.2% of the respondents said forest product collections are serving my household as a second income source, and 12.8% of them have ranked forest products as the third option for their livelihood ［Table 2］. This finding is similar with many studies conducted in different areas in Ethiopia [79]. For instance, in the Yayo district, the same study area, in the Sheka zone, and in the Bale Mountains, forest products were revealed as the primary source of income, with contributions of 54%, 49%, and 44.7%, followed by crop production [80, 81]. In contrast, other studies have indicated that forest products contribute as the fourth most important source of livelihood for households. For instance, at Liban Woreda, Borena, southern Ethiopia (32%), and at Gore District, southwest Ethiopia, similar agro-ecology (23%) [82, 83].

Table 2: Forest products priority to support the household's livelihood among study participants in Yayo coffee forest Biosphere Reserve, Southwest Ethiopia, 2024

Preference Category	No. of Respondents	Percentage (%)	Rank
General dependency on forest products	65	43.9%	1
Forest products as 1st priority	36	37.8%	2
Forest products as 2nd priority	24	16.2%	3
Forest products as 3rd priority	19	12.8%	4
Not engaged in forest products	4	2.7%	5
Total	148	100.0%	

3.7 The major factors of forest depletion

The analysis of the household survey results highlighted several factors contributing to the depletion of forest stocks in the Yayo Coffee Forest Biosphere Reserve. Among the households surveyed, approximately 23.13% of respondents identified agricultural expansion as a major factor in the depletion of forest resources due to the local population's reliance on primary activities such as wood logging, non-timber forest product collection, and farming. The findings in the Yayo Coffee Forest Biosphere Reserve from the survey identify three major causes of forest degradation: lack of indigenous community participation (21.95%), Timber extraction (15.27%) and Urbanization (14%). The Yayo Fertilizer Factory, which has removed about 115 hectares of forestland from the buffer zone of Yayo Biosphere Reserve, is a poor example of industrial investment, which 12.2% of respondents cited as one of the primary causes of forest degradation. This is consistent with research showing that industrial operations significantly speed up deforestation and pose serious risks to biodiversity [Fig.3]. These are strongly supported by findings in some well-accredited studies in comparable environmental and socio-economic contexts. For instance, it is projected that urban expansion would account for the conversion of over 2.5 million hectares of forests globally by 2030, primarily in developing nations, as a testament to the impact of urban spread on forest degradation [84].

The respondents' high agreement with the statement that "the lack of indigenous involvement results in forest depletion" indicates that 73.9% of respondents strongly agree with this statement, which has been confirmed [85]. Similarly, the 75.7% who identified timber extraction as a major cause aligns with previous studies and reports, which rank unsustainable and illegal logging among the leading drivers of forest loss [86, 87].

These trends emphasize the pressing need for more stringent environmental regulation and more environmentally friendly development practices. Moreover, the residents reported that the Yayo Fertilizer Factory displaced a high number of farmers from their farms, and they resorted to daily work as a source of survival [86, 88]. The research revealed that approximately 2,200 farming families were physically displaced, losing their farms, houses, and wild coffee farms as sources of livelihood to accommodate the factory's production [89].

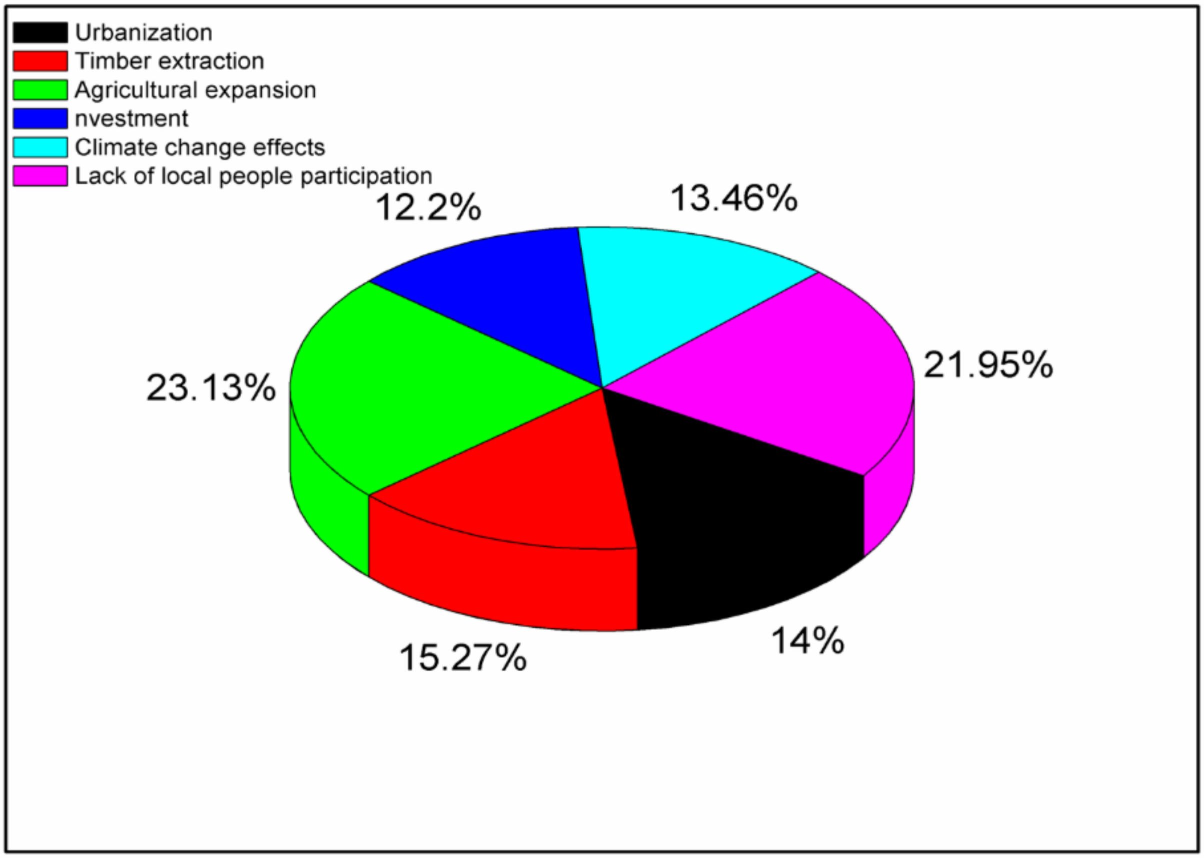

Fig. 2: Factors contributing for the possible depletion of forest stocks in the Yayo coffee forest Biosphere Reserve, Southwest Ethiopia, 2024.

Additionally, the crew saw firsthand how more than 26 low-income families depended on daily wage labor to make ends meet. The Community Forest Coffee Project's (COFCOP) arrival was considered noteworthy in this context because it provided some alternative livelihood support [90].

The coal mining initiative, launched in 2010, aimed to generate employment for thousands of jobless locals in the region. Between 2012 and 2013, over 6,000 skilled and unskilled workers were hired by the Yayo Fertilizer Factory, with most resettled in heavily forested villages such as Achebo and Wutete. However, starting in 2018, coal mining at the Achebo site came under the control of organized youth from displaced families. As a result of the project’s strict security measures, hundreds of villagers were physically displaced and denied access to their coffee farms, forest lands, and water sources. While compensation was provided, it was limited to losses of seasonal crops (like maize, sorghum, and barley), perennial crops (such as mango, pawpaw, and oranges), garden trees, and village homes and structures. The study also revealed that compensation was calculated for only 420 of the 2,200 displaced individuals. For example, compensation for a coffee stand was estimated at just 6.50 birr, and for one hectare of farmland, it was only 400 birr [90].

Other study results indicated a lack of economic opportunities and social amenities in rural areas; rural-urban migration has resulted in a considerable population increase in many sub-Saharan African cities [91].

3.8 Population growth and forest cover change

According to the statistical findings from the household survey, the main drivers of this activity were identified as farmland expansion (40.5%), forest product collection (29.6%), settlement (12%), and overgrazing (17.9%), with these factors emerging as the most significant contributors (Fig. 4). Ethiopia continues to have one of the highest population growth rates in sub-Saharan Africa, 2.5% to 3% annually, which adds pressure to land and other natural resources [92]. Over the past few decades, the region has been a big hub for migrants, with both rising numbers and rising ethnic diversity. Other groups who settled in the area include people from the Southern Nations, Nationalities, and Peoples' Region (SNNPR), Amhara, Tigray, and eastern Oromia [93]. Such movements relate to broader national patterns of domestic migration, often predicated on voluntary migration as well as government-sponsored resettlement programs aimed toward relatively sparsely populated forest zones [94].

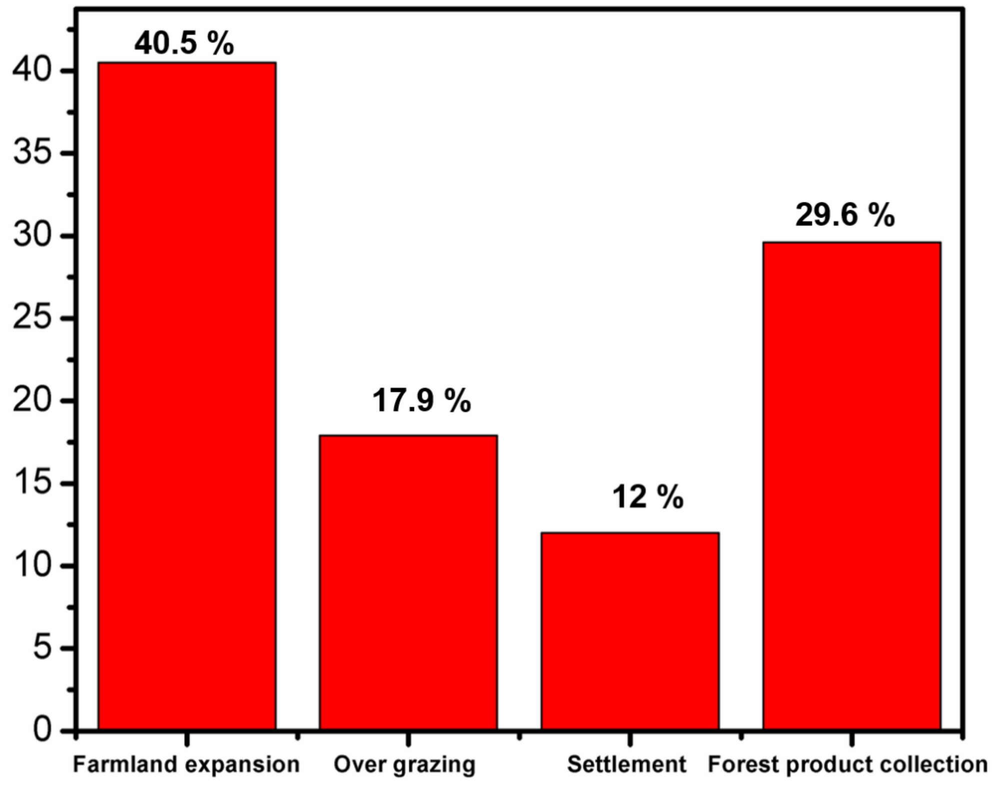

Fig. 3: Forest land Conversion in the Yayo coffee forest Biosphere Reserve, Southwest Ethiopia, 2024.

Household survey reports that 39.2% of the respondents view forest product collection, mainly firewood and forest coffee, as a leading cause of forest degradation. Hurumu Woreda informants report widespread overexploitation of tree species like Cordia africana for illegal timber [Fig. 5]. This aligns with general trends in Ethiopia and sub-Saharan Africa, where wood fuel and timber overextraction drive deforestation [95]. Cordia africana, of economic value for timber and shade, is most susceptible, threatening biodiversity and agroforestry. Despite its importance for livelihood, forest product collection accelerates degradation, particularly when combined with agricultural encroachment and settlement [96].

Farmland expansion, identified by 35.8% of respondents, is a leading cause of forest degradation in the area, while settlement activities contribute to 18.9% of the degradation [Table 5]. Overgrazing, however, is not as prominent since it entails the use of certain grazing fields (kaloo) that are different from forests. Similar findings in the Ethiopian highlands show that spatial land-use management, like exclosures, can avert degradation [97, 98]. In contrast, regions lacking these management systems, such as Afar and Borana, are facing severe soil degradation and loss of biodiversity, largely driven by uncontrolled overgrazing and the absence of sustainable land-use practices [99, 100].

3.9 Demographic Trends from 1984 to 2024 and forest cover change satellite image result

Population data for the past 40 years were obtained from the Central Statistical Agency (CSA), Jimma Branch, based on the Ethiopian Population Censuses conducted in 1984 and 1994, as well as projections from the 2007 census. The population of Yayo district increased from 29,837 in 1984 to 52,851 in 1994, then to 83,579 in 2007, and further to 139,001 by 2024. Similarly, the population of Hurumu district was 27,586 in 1984, rising to 42,667 in 1994, 106,294 in 2007, and 165,143 by 2024. In Dorani district, the population was 20,015 in 1984, increased to 36,705 in 1994, reached 58,938 in 2007, and grew to 97,491 by 2024 (Fig. 5).

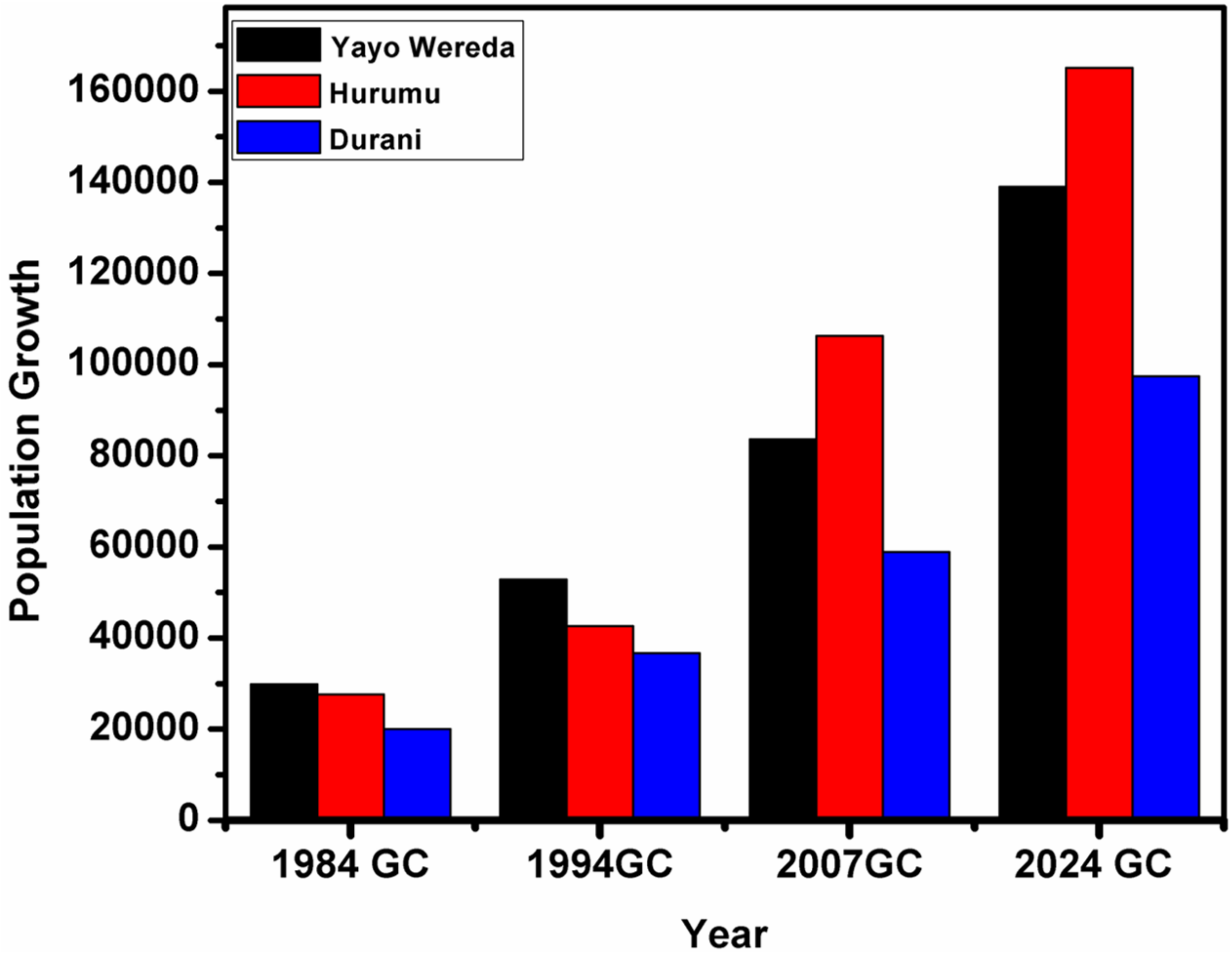

Fig.4: Demographic Changes in the Yayo coffee forest Biosphere Reserve from 1984 to 2024, Southwest Ethiopia [Yayo, Hurumu, and Doreni Districts]

The forest cover change in Yayo Biosphere Reserve over the past 40 years (from 1984 to 2024) is presented as a satellite image comparison. Landsat 5 TM for 1984, Landsat 5 TM and ETM for 1994, and Landsat 7 TM and ETM+ for 2024, each with a 30-meter resolution satellite image cover, showed Forest coverage decreased from 120087.2 hectares to 100772.9 hectares, or by 11.6%, over the 40-year period ［Table 3］. Forest coverage is replaced by agricultural land, and settlement land is the dominant land use type in the study area, which is divided into three compartments: core, buffer, and transitional zones, within the Yayo Biosphere Reserve, and LANDSAT/TM satellite images from 1986 to 1990 show that Ethiopia's forest cover had since then been reduced to 3.93%, or 45,055 sq km [101, 102].

The Yayo Coffee Forest Biosphere Reserve is a significant ecological location that sequesters carbon and mitigates climate change, which makes it both a national and global priority [103]. Agroforestry systems in the region, particularly those for coffee grown under shade, are a

sustainable land use practice that conserves the environment and promotes local development and illustration of a climate-resilient approach in that they enhance carbon sequestration and sustain rural livelihoods [104].

The identification of these land use changes as significant contributors to the region's forest degradation further supported the findings of statistical analysis and satellite imaging, and it makes it exceedingly dangerous for the biosphere reserve to maintain the biodiversity that made it possible for it to be identified and About 15 households have unlawfully established on 8 hectares of the biosphere reserve's core zone, according to data acquired from focus groups and key informant interviews with farmers in Yayo District, particularly in the Kori area close to Sayi Forest [105]. Similarly, in Ilu Abba Dinka Kebele, Janeh area, about 28 households have settled on 14 hectares of the core zone. In total, approximately 22 hectares of forest in the core zone have been cleared due to agricultural expansion and coffee production. Additionally, in some areas, particularly in Gechi Kebele, local people have raised concerns about the planting of exotic tree species, which they believe could rapidly threaten and negatively affect the indigenous species by clearing the native vegetation [106].

The present results align with a study conducted in the Hawa Galan district of Kelem Wollega, Ethiopia, which found that forest cover in the district was negatively correlated with both the district's overall population and the population within the forest area [107]. Another study reported that population growth has intensified pressure on scarce natural resources, leading to the degradation and destruction of forests and woodlands [108].

Table 3: Satellite Images Results of Land Use Land Cover in the Yayo coffee forest Biosphere Reserve from 1984 to 2024, Southwest Ethiopia

**Districts**	**Land use land cover**	**1984**		**1994**		**2024**	
		Area (Ha)	%	Area (Ha)	%	Area (Ha)	%
Doreni	Forest	29030.1	63.1	25395.59	55.2	24889.52	54.1
	Non-Forest land	16976.4	36.9	20610.91	44.8	21116.98	45.9
	Total	46006.5	100	46006.5	100	46006.5	100
Hurumu	Forest	30848.4	66.3	29638.65	63.7	24241.35	52.1
	Non-Forest	15680.1	33.7	16889.85	36.3	22287.15	47.9
	Total	46528.5	100	46528.5	100	46528.5	100
Yayo	Forest land	60208.67	74.5	57703.34	71.4	51642.06	63.9
	Non-Forest land	20608.34	25.5	23113.66	28.6	29174.94	36.1
	Total	80817	100	80817	100	80817	100
Grand total Forest land		120087.17	69.3	112737.58	65	100772.93	58.1

3.10 Socio-Economic Determinants of Forest Dependency and Conservation Behavior

The Pearson Correlation analysis results indicate a strong negative correlation between forest cover and population growth rate over the past 40 years in the surveyed forest biosphere reserve, with a coefficient value of -0.998. This negative value confirms an inverse relationship between population growth and forest cover, meaning that as population growth increases, forest area decreases, and vice versa [109]. The statistical analysis also shows that this correlation is significant at the 0.05 confidence level ($P < 0.05$) ［Table 4］. In line with the above findings, the impact and pressure of population on land use and land cover (LULC) changes are strongly influenced by population density. Pearson correlation analysis showed that forest cover (P = -0.006) is negatively associated with population pressure on forests [109].

Table 4: Pearson Correlation analysis of Land Use Land Cover in the Yayo coffee forest Biosphere Reserve from 1984 to 2024, Southwest Ethiopia

Factors	Population growth	Forest cover change	Year of change
Population growth Pearson Correlation	1	-0.998*	0.999*
Sig. value		0.043	0.020

A study of 148 households in the Yayo Coffee Forest Biosphere Reserve identified key socio-economic factors influencing forest dependence. Multiple linear regression analysis showed that household size (β = -0.32), income (β = -0.0011), and education (β = -0.21) were all significantly and negatively associated with forest dependency ($p < 0.001$ for all). Larger families, higher income, and better education were linked to reduced reliance on forest resources, likely due to diversified livelihoods and greater access to alternatives. These findings support previous research on the socio-economic drivers of forest use and conservation [110]. The inverse relationship between education and forest dependency (β = -0.21, $p < 0.001$), and its positive contribution to conservation behavior (β = 0.31, $p < 0.001$), the environmental awareness is enhanced and pressure on forest resources is reduced through education [111]. Conservation behavior was analyzed using a logistic regression model, with a binary variable indicating whether households participated in conservation. Education had a positive and significant effect (β = 0.31, $p < 0.001$), suggesting that higher education promotes environmental awareness. Income also showed a positive association (β = 0.0017, p = 0.005), implying that wealthier households are more likely to engage in sustainable practices. Similarly, household size was positively linked to conservation behavior (β = 0.28, p = 0.002), possibly due to greater labor availability for community forest management activities [Table 5].

Table 5: Socio-Economic Determinants of Forest Dependency and Conservation Behavior in Yayo coffee forest Biosphere Reserve, Southwest Ethiopia, 2024 [n = 148].

Variable	Linear Regression – Dependency on Forest Resources		Logistic Regression – Conservation Behavior	
	Coefficient (A)	*p-value*	Coefficient (B)	*p-value*
Family Size	-0.32	0.000	0.28	0.002
Income Level (ETB)	-0.0011	0.000	0.0017	0.005
Education status (Years)	-0.21	0.000	0.31	0.000

Similarly, income's role in decreasing forest dependency (β = -0.0011, $p < 0.001$) and increasing conservation participation (β = 0.0017, p = 0.005) aligns with the finding that income diversification enables households to meet their demands without over-exploiting forests [112].

Although it appears contradictory to assume that family size would be positively associated with conservation conduct, this study found that larger families were less dependent on the forest ($\beta = -0.32$, $p < 0.001$) and more involved in conservation activities ($\beta = 0.28$, $p = 0.002$), consistent with those who argued that larger families are capable of rendering more effort and labor towards conservation [113].

4 CONCLUSION AND IMPLICATIONS

The Yayo Coffee Forest Biosphere Reserve, which supports biodiversity conservation and sustainable development, is facing threats from population growth, agricultural encroachment, forest product extraction, and unregulated investments like coal mining. Deforestation is primarily driven by settlement and coffee land clearance, poor local involvement, poverty, and weak institutional coordination. To ensure long-term conservation, community participation, customary institutions, coffee production outside the forest, and impact assessments of extractive activities are crucial. Good governance, harmonization of customary systems with government conservation policies, and guidelines for resource management and local acceptance are also essential. The government must ensure investment supports local people and does not displace them, ensuring sustainable biosphere use and resilient livelihoods.

Furthermore, the finding suggests the Yayo Coffee Forest Biosphere Reserve is a key example of community-based conservation efforts that contribute to global agricultural climate change resilience. As the world grapples with climate change and biodiversity loss, biosphere reserves like Yayo should be prioritized for sustainable development and climate adaptation. Increasing household income can reduce pressure on forest ecosystems, and alternative income-generating opportunities like eco-tourism, apiculture, coffee certification schemes, and agroforestry-based enterprises can be promoted. Current forest restoration efforts often prioritize ecological goals over socio-economic development, emphasizing the need for holistic approaches that consider education, income, and household structure in forest use and conservation.

ABBREVIATIONS

CBOs: Community Based Organization; **DAs**: Developmental Agents; **EARO**: Ethiopian Agricultural Research Organization; **FAO:** Food and Agricultural Organization; **IUCN**: International Union for Conservation of Nature and Natural Resources; m.a.s.l.: Meter above sea

level; **MAB**: Program on Man and the Biosphere; **NGOs:** Non-Governmental Organizations; **SPSS**: Statistical Package for Social Sciences; **STCP**: Sustainable Tree Crops Program; **UNESCO**: United Nations Educational, Scientific and Cultural Organization; and **ZEF**: Zentrum fur Entwicklungsforschung (Center for Development Research).

DATA AVAILABILITY STATEMENT

The original contributions of the study are provided in the article/Supplementary Material; any additional inquiries can be directed to the corresponding author.

SUPPORTING INFORMATION

- S2: Figure S2: Satellite Images Results of Land Use Land Cover in the Yayo coffee forest Biosphere Reserve from 1984 to 2024, Southwest Ethiopia [Hurumu, Doreni and Yayo districts]

AUTHOR CONTRIBUTIONS

FM and AA, and TJ: Conceptualization, methodology, and software. TJ: formal analysis, FM: writing original draft preparation, TJ and FM; writing review and editing. All authors have read and agreed to the published version of the manuscript.

FUNDING

The authors have not declared a specific grant for this research from any funding agency in the public, commercial or not-for-profit sectors.

CONFLICTS OF INTEREST

The authors declare no conflicts of interest.

REFERENCES

1. Bonan, G.B., Forests and climate change: forcings, feedbacks, and the climate benefits of forests. science, 2008. **320**(5882): p. 1444-1449.

2. Canton, H., Food and agriculture organization of the United Nations—FAO, in The Europa directory of international organizations 2021. 2021, Routledge. p. 297-305.

3. Kumar, R., A. Kumar, and P. Saikia, Deforestation and forests degradation impacts on the environment, in Environmental degradation: Challenges and strategies for mitigation. 2022, Springer. p. 19-46.

4. Ngo, T.T.H., et al., Forest-related culture and contribution to sustainable development in the northern mountain region in Vietnam. Forest and Society, 2021. **5**(1): p. 32-47.

5. Odum, E. and G. Barrett, Fundamentals of Ecology.(5thedn). Belmont, Thomson Brooks/Cole, 2005.

6. Inversini, A., et al., The rise of regenerative hospitality. Journal of Tourism Futures, 2024. **10**(1): p. 6-20.

7. Nkonya, E., et al., Economics of land degradation in sub-Saharan Africa. Economics of land degradation and improvement–a global assessment for sustainable development, 2016: p. 215-259.

8. Vlek, P.L., A. Khamzina, and L.D. Tamene, Land degradation and the sustainable development goals: Threats and potential remedies. 2017.

9. Osman, K.T., Soil degradation, conservation and remediation. Vol. 820. 2014: Springer.

10. Sanz, M.J., et al. Sustainable land management contribution to successful land-based climate change adaptation and mitigation: A report of the science-policy interface. in Bonn, Germany: United Nations Convention to Combat Desertification (UNCCD). 2017.

11. Kidane, B., et al., Journal of Agriculture and Food Research.

12. Ramankutty, N., et al., Global land-cover change: Recent progress, remaining challenges. Land-use and land-cover change: local processes and global impacts, 2006: p. 9-39.

13. Kassaye, S.M., et al., Quantifying the climate change impacts on the magnitude and timing of hydrological extremes in the Baro River Basin, Ethiopia. Environmental Systems Research, 2024. **13**(1): p. 1-15.

14. Chofore, A.T., Native tree species based afforestation/reforestation for carbon sequestration: contributions to sustainable development through clean development mechanisms in Ethiopia. 2021: University of South Africa (South Africa).

15. Kovačević, V., et al., 6. Adaptation to climate change in agriculture–status, gaps and recommendations in Serbia. CLIMATE CHANGE ADAPTATION IN AGRICULTURE–STATUS AND PROSPECTS IN WESTERN, 2024: p. 271.

16. Muluneh, T., A. Belayneh, and M. Chimdessa, DIVERSITY AND CONSERVATION STATUS OF NON-TIMBER FOREST PRODUCTS (NTFPS) IN AMIGNA DISTRICT, EAST ARSI ZONE OF OROMIA REGIONAL STATE. 2023, Haramaya University.

17. Prabawani, B., et al., Socioeconomic perspective of agroforestry development in Central Java. Environmental and Sustainability Indicators, 2024. **22**: p. 100354.

18. Tulu, Z.J., Institutions, Incentives and Conflict in Coffee Forest Use and Conservation: the Case of Yayo Forest in Iluu Abba Bora Zone, Southwest Ethiopia. 2010.

19. Gole, T.W. and F. Senbeta, Sustainable management and promotion of forest coffee in Bale, Ethiopia. Bale Eco-Region Sustainable Management Programme SOS Sahel/FARM-Africa, Addis Ababa, 2008.

20. Assen, Y.M., et al., Climate change threats to UNESCO-Designated world heritage sites: empirical evidence from Konso cultural landscape, Ethiopia. Sustainability, 2024. **16**(19): p. 8442.

21. Donoghue, M.J., A phylogenetic perspective on the distribution of plant diversity. Proceedings of the National Academy of Sciences, 2008. **105**(supplement_1): p. 11549-11555.

22. Abera, W., et al., Characterizing and evaluating the impacts of national land restoration initiatives on ecosystem services in Ethiopia. Land Degradation & Development, 2020. **31**(1): p. 37-52.

23. Duguma, M.S., D.H. Feyssa, and L. Biber-Freudenberger, Agricultural biodiversity and ecosystem services of major farming systems: a case study in Yayo Coffee Forest Biosphere Reserve, Southwestern Ethiopia. Agriculture, 2019. **9**(3): p. 48.

24. Bramel, P., et al., Global conservation strategy for coffee genetic resources. Crop Trust and World Coffee Research, 2017. **72**.

25. Madeira, F., et al., The EMBL-EBI Job Dispatcher sequence analysis tools framework in 2024. Nucleic acids research, 2024. **52**(W1): p. W521-W525.

26. Rawal, R., et al., The himalayan biodiversity: richness, representativeness, uniqueness and life-support values. GB Pant Institute of Himalayan Environment and Development (GBPIHED), Kosi-Katarmal, Almora, Uttarakhand, India, 84pp, 2013.

27. Sang, W., Plant diversity patterns and their relationships with soil and climatic factors along an altitudinal gradient in the middle Tianshan Mountain area, Xinjiang, China. Ecological research, 2009. **24**(2): p. 303-314.

28. McGraw, D.M., The CBD-key characteristics and implications for implementation. Rev. Eur. Comp. & Int'l Envtl. L., 2002. **11**: p. 17.

29. Agidew, A.-m.A. and K. Singh, The implications of land use and land cover changes for rural household food insecurity in the Northeastern highlands of Ethiopia: the case of the Teleyayen sub-watershed. Agriculture & Food Security, 2017. **6**(1): p. 56.

30. Hassen, A., et al., Local People's perception of forest ecosystem services, traditional conservation, and management approaches in North Wollo, Ethiopia. Journal of Environmental Management, 2023. **330**: p. 117118.

31. Kalele, D.N., et al., Climate change impacts and relevance of smallholder farmers’ response in arid and semi-arid lands in Kenya. Scientific African, 2021. **12**: p. e00814.

32. Kuma, H.G., F.F. Feyessa, and T.A. Demissie, Land-use/land-cover changes and implications in Southern Ethiopia: evidence from remote sensing and informants. Heliyon, 2022. **8**(3).

33. Gole, T.W., et al., Floristic composition and environmental factors characterizing coffee forests in southwest Ethiopia. Forest Ecology and Management, 2008. **255**(7): p. 2138-2150.

34. Tadesse, B., et al., Foot and mouth disease outbreak investigation and estimation of its economic impact in selected districts in northwest Ethiopia. Veterinary medicine and science, 2020. **6**(1): p. 122-132.

35. Tafere, H.B. and S.L. Woreta, Tourism Potential and Its Development Challenges in Jabi Tehnan Woreda, West Gojjam Zone, Ethiopia.

36. Walle, Y. and D. Nayak, Factors determining the participation of natural resource cooperative members in forest management: A study of dry forest area in Ethiopia. Trees, Forests and People, 2022. **8**: p. 100241.

37. Mengistu, D.A. and A.T. Salami, Application of remote sensing and GIS inland use/land cover mapping and change detection in a part of south western Nigeria. African Journal of Environmental Science and Technology, 2007. **1**(5): p. 99-109.

38. Yauch, C.A. and H.J. Steudel, Complementary use of qualitative and quantitative cultural assessment methods. Organizational research methods, 2003. **6**(4): p. 465-481.

39. Getahun, D. and E.T. Keno, Attitudes and perceptions of the local community towards Yayo coffee forest biosphere reserve, ilu abba bora zone of Oromia national regional state. Ethiopian Journal of Science and Sustainable Development, 2019. **6**(1): p. 79-90.

40. Etana, D., et al., Climate change, in-situ adaptation, and migration decisions of smallholder farmers in central Ethiopia. Migration and Development, 2022. **11**(3): p. 737-761.

41. Newing, H., et al., Conducting research in conservation. Vol. 775. 2011: Routledge Oxfordshire.

42. Bernard, H.R., Research methods in anthropology: Qualitative and quantitative approaches. 2017: Bloomsbury Publishing PLC.

43. Woldie, B.A. and S.A. Tadesse, Views and attitudes of local people towards community versus state forest governance in Tehulederi District, South Wollo, Ethiopia. Ecological Processes, 2019. **8**(1): p. 4.

44. Krueger, R.A. and M.A. Casey, Focus group interviewing. Handbook of practical program evaluation, 2015: p. 506-534.

45. Bazeley, K. and A. Hayton, Practical cattle farming. 2013: Crowood.

46. Adhikari, B., S. Di Falco, and J.C. Lovett, Household characteristics and forest dependency: evidence from common property forest management in Nepal. Ecological economics, 2004. **48**(2): p. 245-257.

47. Benjamin, D.J., et al., Redefine statistical significance. Nature human behaviour, 2018. **2**(1): p. 6-10.

48. Zhang, Z., Residuals and regression diagnostics: focusing on logistic regression. Annals of translational medicine, 2016. **4**(10): p. 195.

49. Braun, V. and V. Clarke, Using thematic analysis in psychology. Qualitative research in psychology, 2006. **3**(2): p. 77-101.

50. Rahman, M.H. and K. Alam, Forest dependent indigenous communities' perception and adaptation to climate change through local knowledge in the protected area—A Bangladesh case study. Climate, 2016. **4**(1): p. 12.

51. Raimondo, E. and K.E. Newcomer, Mixed-methods inquiry in public administration: The interaction of theory, methodology, and praxis. Review of public personnel administration, 2017. **37**(2): p. 183-201.

52. Snyder, L.R., J.J. Kirkland, and J.L. Glajch, Practical HPLC method development. 2012: John Wiley & Sons.

53. Faaz, M. and Z.N. Khan, A study of academic achievement of upper primary school students in relation to their socio-economic status. Asian Journal of Research in Social Sciences and Humanities, 2017. **7**(6): p. 121-127.

54. Cerqua, A., C. Gauthier, and M. Dembélé, Education policy, teacher education, and pedagogy: A case study of UNESCO. Annual Review of Comparative and International Education 2014, 2014: p. 235-266.

55. Smith-Greenaway, E., Maternal reading skills and child mortality in Nigeria: a reassessment of why education matters. Demography, 2013. **50**(5): p. 1551-1561.

56. Alderman, H. and J.R. Behrman, Reducing the incidence of low birth weight in low-income countries has substantial economic benefits. The World Bank Research Observer, 2006. **21**(1): p. 25-48.

57. Fetensa, A.D., M.S. Dalu, and T. Bedada, Economic contribution of forest resource at household level in the bale mountains eco-region redd+ project, Southern Ethiopia. Journal of Resources Development and Management, 2019. **53**: p. 7-27.

58. Meleaku, T., PRODUCTION EFFICIENCY AND MARKET SUPPLY OF SORGHUM: THE CASE OF SMALLHOLDER FARMERS IN KAFTA-HUMERA DISTRICT OF TIGRAY, ETHIOPIA. 2018, Haramaya university.

59. Bishu, K.G., Risk management and the potential of cattle insurance in Tigray, Northern Ethiopia. 2014.

60. Hein, L. and F. Gatzweiler, The economic value of coffee (Coffea arabica) genetic resources. Ecological economics, 2006. **60**(1): p. 176-185.

61. Stellmacher, T. and P.P. Mollinga, The institutional sphere of coffee forest management in Ethiopia: local level findings from Koma forest, Kaffa zone. International journal of social forestry, 2009. **2**(1): p. 43-66.

62. Snyder, T.D. and S.A. Dillow, Digest of Education Statistics, 2011. NCES 2012-001. National Center for Education Statistics, 2012.

63. Lemenih, M. and H. Kassa, Re-greening Ethiopia: history, challenges and lessons. Forests, 2014. **5**(7): p. 1717-1730.

64. Gebremariam, A. and H. Gebremariam, Contraceptive use among lactating women in Ganta-Afeshum District, Eastern Tigray, Northern Ethiopia, 2015: a cross sectional study. BMC pregnancy and childbirth, 2017. **17**(1): p. 421.

65. Ojulu, O.M., Large-scale land acquisitions and minorities/indigenous peoples' rights under ethnic federalism in Ethiopia. A Case Study of Gambella Regional State. 2014.

66. Daba, A., et al., Weed species composition and abundance in the main coffee production systems and regions of Ethiopia. South African Journal of Plant and Soil, 2022. **39**(1): p. 41-55.

67. Tura, N., et al., Unlocking circular business: A framework of barriers and drivers. Journal of cleaner production, 2019. **212**: p. 90-98.

68. Gufu, G.D., et al., Do invasive exotic and native freshwater plant species respond similarly to low additional nitrate doses? Aquatic Botany, 2018. **151**: p. 1-8.

69. Terfassa Fida, G., B.N. Baatuuwie, and H. Issifu, Dynamics of land use/cover change and its drivers during 1992–2022 in Yayo Coffee Forest Biosphere Reserve, Southwestern Ethiopia. Sustainable Environment, 2024. **10**(1): p. 2374119.

70. Ostrom, E., Governing the commons: The evolution of institutions for collective action. 1990: Cambridge university press.

71. Cleaver, D.J., Z. Wang, and I. Gursul, Bifurcating flows of plunging aerofoils at high Strouhal numbers. Journal of Fluid Mechanics, 2012. **708**: p. 349-376.

72. Agrawal, A., Common property institutions and sustainable governance of resources. World development, 2001. **29**(10): p. 1649-1672.

73. Tesema, S.F., Impact of technological change on household production and food security in smallholders agriculture: the case of wheat-tef based farming systems in the central highlands of Ethopia. 2006: Cuvillier Verlag.

74. Tulu, A., The Practice and Challenges of School-Based Teachers' Continuous Professional Development: A Case of Government Secondary Schools of Hawassa City in Ethiopia. Educational Research and Reviews, 2019. **14**(1): p. 33-43.

75. Derso, W.G. and B. Gebremichael, The leasehold system and drivers of informal land transactions in bahir dar city, Ethiopia. Journal of Eastern African Studies, 2022. **16**(3): p. 415-433.

76. Cleaver, F., Development through bricolage: Rethinking institutions for natural resource management. 2017: Routledge.

77. Yusuf, A., THE DETERMINANTS OF RURAL HOUSEHOLDS FOOD SECURITY AND COPING STRATEGIES: THE CASE OF META DISTRICT, EAST HARARGHE ZONE OF OROMIA NATIONAL REGIONAL STATE OF ETHIOPIA. 2017, Haramaya university.

78. Eshetu, A.A., Forest resource management systems in Ethiopia: Historical perspective. International Journal of Biodiversity and Conservation, 2014. **6**(2): p. 121-131.

79. Asfaw, A. and L. Etefa, The contribution of non-timber forest products to the rural livelihood: The case of yayo district, illu ababora zone, oromia regional state, western Ethiopia. International Journal of Applied Agricultural Research, 2017. **12**(2): p. 157-169.

80. Moloro, H.B. and T. Abebe, Review on socio economic and ecological role of non timber forest products in South Western Ethiopia. Ethiopian Journal of Economics, 2022. **3**(2): p. 46-65.

81. Heron, R. and W. Juju, The marina-sustainable solutions for a profitable business. 2012: Lulu. com.

82. Mulugeta, S.B., Drought, vulnerability and adaptation: risk of food and livelihoods insecurity for pastoralists and agro-pastoralists in Borana zone, southern Ethiopia. 2023, University of Nairobi.

83. Birhanu, A.G., A. Gambardella, and G. Valentini, Bribery and investment: Firm-level evidence from A frica and L atin A merica. Strategic Management Journal, 2016. **37**(9): p. 1865-1877.

84. Seto, K.C., B. Güneralp, and L.R. Hutyra, Global forecasts of urban expansion to 2030 and direct impacts on biodiversity and carbon pools. Proceedings of the National Academy of Sciences, 2012. **109**(40): p. 16083-16088.

85. Agrawal, A. and C.C. Gibson, Enchantment and disenchantment: the role of community in natural resource conservation. World development, 1999. **27**(4): p. 629-649.

86. Alemu, B., et al., Land use and land cover changes and associated driving forces in north western lowlands of Ethiopia. International research journal of agricultural science and soil science, 2015. **5**(1): p. 28-44.

87. Herforth, A., et al., Cost and affordability of healthy diets across and within countries: Background paper for The State of Food Security and Nutrition in the World 2020. FAO Agricultural Development Economics Technical Study No. 9. Vol. 9. 2020: Food & Agriculture Org.

88. Teketay, D., et al., Enhancement of diversity, stand structure and regeneration of woody species through area exclosure: the case of a mopane woodland in northern Botswana. Ecological Processes, 2018. **7**(1): p. 5.

89. Wiersum, K.F. and T.B. Endalamaw, Governing forests for provisioning services: the example of honey production in southwest Ethiopia, in Governing the Provision of Ecosystem Services. 2012, Springer. p. 303-318.

90. Wiersum, K. and T. Endalamaw, Governing the provision of ecosystem services. Studies in ecological economics, vol 4. 2013.

91. Menashe-Oren, A. and D.A. Sánchez-Páez, Male fertility and internal migration in rural and urban Sub-Saharan Africa. European Journal of Population, 2023. **39**(1): p. 10.

92. Wassie, S.B., Natural resource degradation tendencies in Ethiopia: a review. Environmental systems research, 2020. **9**(1): p. 1-29.

93. Groth, O., The Great Remobilization: Strategies and Designs for a Smarter Global Future. 2023: MIT Press.

94. Woube, M., Effects of resettlement schemes on the biophysical and human environments: The case of the Gambela Region, Ethiopia. 2005: Universal-Publishers.

95. Morrow, N., Autochthonous and introduced stores of biomass value: Measuring resilience outcomes of enset and eucalyptus as green assets in three representative smallholder farm systems of Ethiopia. 2021, University of Maryland, College Park.

96. Molla, T., et al., Diversity of woody species in traditional agroforestry practices in Wondo district, south-central Ethiopia. Heliyon, 2023. **9**(2).

97. Descheemaeker, K., et al., Runoff on slopes with restoring vegetation: a case study from the Tigray highlands, Ethiopia. Journal of hydrology, 2006. **331**(1-2): p. 219-241.

98. Wolde, M. and E. Veldkamp. Impacts of land use changes on soil nutrients and erosion in Tigray, Ethiopia. in Conference on International Agricultural Research for Development, Deutscher Tropentag. 2005.

99. Worku Annota, C., S. Asfaw, and S. Tekaligni, DETERMINANTS OF SMALLHOLDER FARMERS'CHOICE OF ADAPTATION STRATEGIES TO THE EFFECTS OF CLIMATE CHANGE: THE CASE OF CHIRO DISTRICT, WEST HARARGHE ZONE, OROMIA REGION, ETHIOPIA. 2021, Haramaya University.

100. Amsalu, A. and J. De Graaff, Determinants of adoption and continued use of stone terraces for soil and water conservation in an Ethiopian highland watershed. Ecological economics, 2007. **61**(2-3): p. 294-302.

101. Nurrochmat, D.R., et al., Transformation of agro-forest management policy under the dynamic circumstances of a two-decade regional autonomy in Indonesia. Forests, 2021. **12**(4): p. 419.

102. Melese, S.M., Effect of land use land cover changes on the forest resources of Ethiopia. International Journal of Natural Resource Ecology and Management, 2016. **1**(2): p. 51.

103. Guala, A., et al., Wall shear stress predicts aortic dilation in patients with bicuspid aortic valve. Cardiovascular Imaging, 2022. **15**(1): p. 46-56.

104. Berhanu, A.A., Vulnerabilities, Response Strategies, and Resilience of Smallholder Farmers to Climate Change in the Upper Blue Nile Basin of Ethiopia. 2025.

105. Gebretsadik, T., Causes for biodiversity loss in Ethiopia: A review from conservation perspective. Journal of Natural Sciences Research, 2016. **6**(11): p. 32-40.

106. Lung, T. and G. Schaab, A comparative assessment of land cover dynamics of three protected forest areas in tropical eastern Africa. Environmental monitoring and assessment, 2010. **161**(1): p. 531-548.

107. Yadeta, T., et al., Recent resettlement programs, as drivers for Afromontane forest loss in the Hawa-Galan district of Ethiopia. Cogent Social Sciences, 2022. **8**(1): p. 2088462.

108. Mohammed, A., S. Bahmud, and A. Abdulahi, The status of forest development in Ethiopia: challenges and opportunities: a review paper. 2023.

109. Belayneh, Y., et al., Forest cover change and its driving forces in Fagita Lekoma District, Ethiopia. Journal of forestry research, 2020. **31**(5): p. 1567-1582.

110. Babulo, B., et al., Household livelihood strategies and forest dependence in the highlands of Tigray, Northern Ethiopia. Agricultural systems, 2008. **98**(2): p. 147-155.

111. Tadesse, W., Towards the sustainable conservation, management and utilization of dry forests in Ethiopia. A review, 2021: p. 38.

112. Ango, T.G., REGIONAL AND LOCAL DEVELOPMENT STUDIES. 2005.

113. Seboka, A.D., et al., Potentials and prospects of solid biowaste resources for biofuel production in Ethiopia: a systematic review of the evidence. Biomass Conversion and Biorefinery, 2024. **14**(24): p. 30929-30960.

Response to Reviewer Comments

Manuscript Title: Impact of Population Pressure on Forest Resources Depletion in Yayo Coffee Forest Biosphere Reserve, Ilubabor Zone, Oromia Regional State, Southwest Ethiopia
Manuscript ID: PONE-D-25-21231

Dear PLOS ONE Editorial Team,

We sincerely thank you for the opportunity to revise our manuscript and express our appreciation to the reviewers for their insightful comments and suggestions that have helped us to improve the quality and clarity of our paper.

We have carefully considered all the reviewer's comments and revised the manuscript accordingly. Below we provide a detailed, point-by-point response to the reviewer's remarks. For clarity, we have included each comment in bold followed by our responses in regular text. All changes made in the manuscript are highlighted for your convenience.

Reviewer #1:

Comment #1. Dear authors your study topic is interesting but it needs major revisions, especial grammar and lack of citations are the major problems of the study, please solve this problem and related ones. Also make the mape legevisible for readers!

Response #1. We sincerely thank the reviewer for the constructive feedback and appreciation of the study's topic. In response:

- We have thoroughly revised the manuscript for grammar, clarity, and academic writing quality. To ensure correctness, we conducted a full language edit and proofreading.
- We have reviewed the manuscript and added relevant and up-to-date citations to strengthen the literature support and properly credit prior research.
- The presentation of the Mean Absolute Percentage Error (MAPE) has been improved. Specifically, we have clarified its definition, usage, and interpretation in the methodology and results sections. We also ensured it is clearly labeled and easy to interpret in the tables and figures.

Reviewer #2:

Comment #1.

1. **Nobility of the Study:** Critical Review of the Manuscript

- **Strengths:** The manuscript focuses on a critical environmental issue in a globally significant biosphere reserve. It addresses the pressing need for sustainable forest management amidst growing population pressures.
- **Weaknesses:** The manuscript does not highlight the global implications of its findings. For instance, the impact of forest loss in the Yayo Coffee Forest Biosphere Reserve on carbon sequestration and global biodiversity hotspots could be better contextualized.
- **Recommendation:** Explicitly link the study's implications to global environmental challenges, such as climate change and biodiversity loss.

Response #1.

We sincerely thank the reviewer for the thoughtful evaluation and constructive suggestion. In response, we have revised the introduction, discussion and conclusion sections to better highlight the global significance of our findings. Specifically, we now discuss the broader implications of forest loss in the Yayo Coffee Forest Biosphere Reserve in terms of carbon sequestration, contributions to global biodiversity conservation, and relevance to international climate and sustainability goals. These additions help to clearly position our study within the context of global environmental challenges, such as climate change and biodiversity loss.

The revisions can be found on page2, paragraph 3; page 16, paragraph 2 and page 19, paragraph 2

Comment # 2. Statement of the Problem and Knowledge Gap

• **Strengths:** The problem is articulated in the context of population pressure and forest degradation.

• **Weaknesses:** The knowledge gap lacks specificity. While the manuscript states that population growth affects forest resources, it does not critically assess why current solutions are insufficient or how this study provides a novel perspective.

• **Recommendation:** Expand the introduction to include a critical review of existing solutions and their shortcomings. Clearly articulate how this research addresses those gaps.

Response # 2

We thank the reviewer for this insightful observation. In response, we have revised the introduction to include a more detailed and critical review of existing interventions aimed at mitigating forest degradation under population pressure. We now discuss the limitations of current policy frameworks, enforcement mechanisms, and community-based management approaches, particularly in the context of the Yayo Coffee Forest Biosphere Reserve. Furthermore, we have clearly articulated how our study offers a novel contribution by integrating socio-ecological analysis with geospatial data to assess the effectiveness of existing measures. These revisions help clarify the specific knowledge gap and the unique perspective our study brings.

The revised content can be found on page 3, paragraph 3.

Comment #3: Objective

• **Strengths:** Objectives are clear and focused.
• **Weaknesses:** The objectives could be more concise and aligned directly with the problem statement.
• **Recommendation:** Reframe the objectives to reflect measurable outcomes. For example, "Quantify the impact of population growth on forest cover using satellite imagery from 1984 to 2024."

Response # 3

We appreciate the reviewer's positive feedback and helpful recommendation. In response, we have revised the objectives to make them more concise and explicitly aligned with the problem statement. The updated objectives now clearly reflect measurable outcomes, including the quantification of forest cover change over time and the assessment of population growth impacts using geospatial data from 1984 to 2024. This reframing enhances the clarity and focus of the study and better communicates its purpose to the reader.

The revised objectives are presented in the introduction section on page 3 paragraph 4

Comment #4. Method of Data Collection

• Strengths: The mixed-methods approach adds depth and breadth to the analysis.
• Weaknesses: The manuscript fails to justify the selection of methods, particularly the use of satellite imagery spanning specific years and the choice of SPSS for analysis.
• **Recommendation:** Provide citations for methodological protocols. For instance, why were 1984, 1994, 2004 and 2024 chosen for satellite imagery? What validation steps were taken to ensure accuracy in satellite data interpretation? Include a clear rationale for the statistical tools employed.

Response #4

We thank the reviewer for this valuable feedback and for recognizing the strengths of our mixed-methods approach. In response to the concerns raised:

- We have now provided a detailed justification for the selection of satellite imagery years (1984, 1994, and 2024). These years were chosen based on data availability, major policy or demographic shifts in the region, and their relevance in capturing long-term trends in land cover change. We have added relevant references to support this temporal selection.
- We have also described the image classification and accuracy assessment procedures, including the use of ground truth data, historical Google Earth imagery, and confusion matrices to validate classification results. The methods and validation steps are now clearly stated in the revised methodology section.
- Furthermore, we have included a rationale for using SPSS, explaining its role in analyzing survey data and conducting descriptive and inferential statistical analyses, such as correlation and regression, to examine relationships between population dynamics and forest cover change. Supporting citations have been added where appropriate.

These revisions are included in the methodology section on pages 18 – 21.

Comment # 5: Method of Data Analysis

• Strengths: The use of SPSS and Pearson correlation is appropriate for quantitative data.

• Weaknesses: The analysis lacks depth in exploring causality. For example, while correlation is established, causation is not discussed.

• Recommendation: Include regression analysis or other advanced statistical methods to explore causality. For qualitative data, provide representative quotes or narratives to substantiate thematic findings.

Response #5

We appreciate the reviewer's thoughtful comments and suggestions to enhance the rigor of our data analysis. In response:

- We have extended our quantitative analysis by incorporating multiple linear regression models to better explore causal relationships between population growth variables and forest cover change. The regression results are now included and interpreted in the revised results section.
- In addition to Pearson correlation, the use of regression analysis enables us to assess the strength and direction of potential causal links while controlling for confounding variables.
- For the qualitative data, we have included representative quotes from key informant interviews and focus group discussions to support and illustrate the main themes identified. These narratives provide greater context and depth to the findings.

All additions and revisions are detailed in the results and conclusion sections on pages 21-22

Comment #6. Results

• Strengths: The results are comprehensive and supported by tables and figures.
• Weaknesses: The presentation is cluttered and lacks coherence. The socio-economic data is presented without linking it to the broader research question.

• Recommendation: Simplify tables and figures, ensuring each directly addresses a research objective. Use visual aids like trend lines or pie charts to make data more digestible.

Response #6

We thank the reviewer for acknowledging the comprehensiveness of our results and for the valuable suggestions to improve clarity and coherence. In response:

- We have simplified and reorganized the tables and figures to ensure each one directly corresponds to a specific research objective or key finding. Unnecessary or redundant visuals have been removed, and labels and captions have been improved for clarity.
- To enhance readability, we introduced more visual aids such as trend lines, pie charts, and bar graphs where appropriate—particularly for time-series and categorical socio-economic data. These revisions help highlight key trends and make the data more accessible to readers.
- Additionally, we revised the narrative surrounding the socio-economic data to better connect the findings with the main research question and theoretical framework. This strengthens the linkage between data and interpretation.

The revised figures and results are presented in the results section on pages Fig 1 on page 4, Figure 2 on page 10, figure 3 on page 15, and figure 4 on page 16, figure 5 on page 18, and Table 5 on page 21.

Comment #7. Discussion

• **Strengths:** The discussion connects findings to conservation and socio-economic issues. • **Weaknesses:** There is insufficient engagement with contemporary literature. Key findings are not critically analyzed against previous studies.

• **Recommendation:** Incorporate a comparative analysis with global studies on forest degradation. Critically evaluate the implications of key results, such as the role of local governance in conservation efforts.

Response # 7

We thank the reviewer for this important suggestion to deepen the analytical strength of the discussion. In response:

- We have substantially revised the discussion section to include a broader engagement with recent and relevant global literature on forest degradation, land use change, and population dynamics. Comparative insights from studies in other tropical biosphere reserves and conservation areas (e.g., Southeast Asia, Central Africa, and Latin America) have been incorporated to contextualize our findings.
- We have critically examined how our results align with or diverge from previous studies, particularly regarding the drivers of forest loss and the socio-economic dimensions of resource pressure.
- Moreover, we have expanded our evaluation of the role of local governance in forest conservation. This includes a discussion of community-based forest management, institutional challenges, and the effectiveness of local bylaws and stakeholder engagement, supported by recent studies and best practices from similar settings.

These additions provide a more nuanced understanding of our findings and better position them within the global discourse on forest conservation. The revised discussion is found on pages 7- 20

Comment # 8. Recommendations

- **Strengths:** The recommendations are actionable and community-oriented.
- **Weaknesses:** They are broad and lack prioritization.
- **Recommendation:** Categorize recommendations into immediate, medium-term, and long-term actions. Justify recommendations with data and evidence from the study.

Response # 8

We thank the reviewer for the constructive feedback and for recognizing the community-oriented nature of our recommendations. In response:

- We have revised the recommendations section to categorize proposed actions into **immediate**, **medium-term**, and **long-term** interventions. This prioritization provides a clearer implementation framework for stakeholders.
- Each recommendation is now more explicitly supported by data and findings from our study—for example, deforestation hotspots identified through satellite analysis and community perceptions gathered through surveys and interviews.
- We also clarified the linkage between the proposed actions and the underlying causes of forest degradation identified in the research, ensuring that each recommendation is both evidence-based and aligned with the study objectives.

These improvements are presented in the revised conclusion and recommendations sections on pages 23, and are highlighted accordingly in the updated manuscript.

Comment # 9: Alignment of Research Problem, Objective, Methods, Results, and Discussion

- **Strengths:** The manuscript maintains alignment throughout its sections.
- **Weaknesses:** The linkage between objectives, findings, and recommendations is weak. For example, the methods section does not explain how the data supports specific recommendations.
- **Recommendation:** Create explicit connections between each section. For instance, demonstrate how the satellite imagery data directly informs the policy recommendations.

Response # 9

We thank the reviewer for highlighting the importance of internal consistency and coherence across the manuscript. In response:

- We have revised the **methods** and **discussion** sections to more clearly explain how each type of data collected—particularly the satellite imagery and socio-economic survey results—contributes directly to the study's objectives and informs the recommendations.
- The **discussion** now includes explicit references to how land cover change data (e.g., forest loss from 1984 to 2024) supports targeted policy interventions such as zoning, reforestation efforts, and land use planning.
- We have also refined the **recommendations** section to include cross-references to the specific findings they are based on. This creates a stronger narrative thread from research problem to objective, methods, results, and actionable solutions.
- Where applicable, we included a summary table that links each research objective with the corresponding data, key findings, and resulting recommendations to make these connections more transparent to the reader.

These improvements appear on pages 18 to 21

Comment # 10: Suggestions for Authors

o Refine the introduction to frame the study within a global conservation context.
o Enhance methodological transparency by justifying data collection and analysis choices.
o Integrate more critical analysis in the discussion, contrasting findings with global studies.

Response # 10

We sincerely thank the reviewer for these valuable and constructive suggestions. In response:

- We have refined the **introduction** to more clearly position the study within a **global conservation context**. This includes discussion of the relevance of biosphere reserves to global biodiversity conservation, climate change mitigation, and sustainable development goals. We

have also added citations to recent international studies and frameworks to support this global framing.

- To enhance **methodological transparency**, we have added justifications for our choices in data collection (e.g., selection of satellite imagery years, survey tools) and data analysis (e.g., use of SPSS, regression analysis). Supporting literature has been cited to provide methodological grounding and demonstrate adherence to established protocols.
- The **discussion** section has been revised to incorporate more **critical analysis**, with a comparative perspective that contrasts our findings with similar research conducted in other tropical and subtropical forest regions globally. This includes discussion of consistencies, divergences, and the broader implications of our results in terms of forest governance and conservation policy.

These revisions are reflected in the updated manuscript on pages 7 to 21, and the changes have been clearly marked for ease of review.

We would like to thank the editor and reviewers once again for their constructive comments and valuable suggestions. We believe the revisions have substantially improved the clarity, rigor, and relevance of our manuscript. All changes have been carefully addressed and are highlighted in the revised version. We hope the manuscript now meets the journal's standards and expectations, and we respectfully submit it for your further consideration.

Sincerely,

Fikru Mosisa Hunde (Assistant professor)
On behalf of all authors
Department of Biology, College of Natural Sciences, Mattu University, Ethiopia
fikrumosisa@gmail.com
