# Supplementary material for: Impact of population pressure on forest resources depletion in Yayo coffee forest Biosphere Reserve, Southwest Ethiopia
Source: PLoS One. 2026 Jan 5;21(1):e0324407. doi: 10.1371/journal.pone.0324407 (PMC12768366; doi:10.1371/journal.pone.0324407)
Supplement: Attachment — Submitted filename: Response to Reviewers.docx [file pone.0324407.s005.docx]

**Response to Reviewer Comments**

**Manuscript Title**: Impact of Population Pressure on Forest Resources Depletion in Yayo Coffee Forest Biosphere Reserve, Ilubabor Zone, Oromia Regional State, Southwest Ethiopia
**Manuscript ID**: PONE-D-25-21231

Dear PLOS ONE Editorial Team,

We sincerely thank you for the opportunity to revise our manuscript and express our appreciation to the reviewers for their insightful comments and suggestions that have helped us to improve the quality and clarity of our paper.

We have carefully considered all the reviewer’s comments and revised the manuscript accordingly. Below we provide a detailed, point-by-point response to the reviewer’s remarks. For clarity, we have included each comment in bold followed by our responses in regular text. All changes made in the manuscript are highlighted for your convenience.

**Reviewer #1:**

**Comment #1.** Dear authors your study topic is interesting but it needs major revisions, especial grammar and lack of citations are the major problems of the study, please solve this problem and related ones. Also make the mape legevisible for readers!

**Response #1.** We sincerely thank the reviewer for the constructive feedback and appreciation of the study's topic. In response:

- We have thoroughly revised the manuscript for grammar, clarity, and academic writing quality. To ensure correctness, we conducted a full language edit and proofreading.
- We have reviewed the manuscript and added relevant and up-to-date citations to strengthen the literature support and properly credit prior research.
- The presentation of the Mean Absolute Percentage Error (MAPE) has been improved. Specifically, we have clarified its definition, usage, and interpretation in the methodology and results sections. We also ensured it is clearly labeled and easy to interpret in the tables and figures.

**Reviewer #2:**

**Comment #1.**

1. **Nobility of the Study:** Critical Review of the Manuscript
- **Strengths:** The manuscript focuses on a critical environmental issue in a globally significant biosphere reserve. It addresses the pressing need for sustainable forest management amidst growing population pressures.
- **Weaknesses:** The manuscript does not highlight the global implications of its findings. For instance, the impact of forest loss in the Yayo Coffee Forest Biosphere Reserve on carbon sequestration and global biodiversity hotspots could be better contextualized.
- **Recommendation:** Explicitly link the study's implications to global environmental challenges, such as climate change and biodiversity loss.

**Response #1.**

We sincerely thank the reviewer for the thoughtful evaluation and constructive suggestion. In response, we have revised the introduction, discussion and conclusion sections to better highlight the global significance of our findings. Specifically, we now discuss the broader implications of forest loss in the Yayo Coffee Forest Biosphere Reserve in terms of carbon sequestration, contributions to global biodiversity conservation, and relevance to international climate and sustainability goals. These additions help to clearly position our study within the context of global environmental challenges, such as climate change and biodiversity loss.

The revisions can be found on page2, paragraph 3; page 16, paragraph 2 and page 19, paragraph 2

**Comment # 2.** Statement of the Problem and Knowledge Gap

• **Strengths:** The problem is articulated in the context of population pressure and forest degradation.

• **Weaknesses:** The knowledge gap lacks specificity. While the manuscript states that population growth affects forest resources, it does not critically assess why current solutions are insufficient or how this study provides a novel perspective.

• **Recommendation:** Expand the introduction to include a critical review of existing solutions and their shortcomings. Clearly articulate how this research addresses those gaps.

**Response # 2**

We thank the reviewer for this insightful observation. In response, we have revised the introduction to include a more detailed and critical review of existing interventions aimed at mitigating forest degradation under population pressure. We now discuss the limitations of current policy frameworks, enforcement mechanisms, and community-based management approaches, particularly in the context of the Yayo Coffee Forest Biosphere Reserve. Furthermore, we have clearly articulated how our study offers a novel contribution by integrating socio-ecological analysis with geospatial data to assess the effectiveness of existing measures. These revisions help clarify the specific knowledge gap and the unique perspective our study brings.

The revised content can be found on page 3, paragraph 3.

**Comment #3: Objective**

• **Strengths:** Objectives are clear and focused.

• **Weaknesses:** The objectives could be more concise and aligned directly with the problem statement.
• **Recommendation:** Reframe the objectives to reflect measurable outcomes. For example, "Quantify the impact of population growth on forest cover using satellite imagery from 1984 to 2024."

**Response # 3**

We appreciate the reviewer’s positive feedback and helpful recommendation. In response, we have revised the objectives to make them more concise and explicitly aligned with the problem statement. The updated objectives now clearly reflect measurable outcomes, including the quantification of forest cover change over time and the assessment of population growth impacts using geospatial data from 1984 to 2024. This reframing enhances the clarity and focus of the study and better communicates its purpose to the reader.

The revised objectives are presented in the introduction section on page 3 paragraph 4

**Comment #4. Method of Data Collection**

• Strengths: The mixed-methods approach adds depth and breadth to the analysis.
• Weaknesses: The manuscript fails to justify the selection of methods, particularly the use of satellite imagery spanning specific years and the choice of SPSS for analysis.
• **Recommendation:** Provide citations for methodological protocols. For instance, why were 1984, 1994, 2004 and 2024 chosen for satellite imagery? What validation steps were taken to ensure accuracy in satellite data interpretation? Include a clear rationale for the statistical tools employed.

**Response #4**

We thank the reviewer for this valuable feedback and for recognizing the strengths of our mixed-methods approach. In response to the concerns raised:

- We have now provided a detailed justification for the selection of satellite imagery years (1984, 1994, and 2024). These years were chosen based on data availability, major policy or demographic shifts in the region, and their relevance in capturing long-term trends in land cover change. We have added relevant references to support this temporal selection.
- We have also described the image classification and accuracy assessment procedures, including the use of ground truth data, historical Google Earth imagery, and confusion matrices to validate classification results. The methods and validation steps are now clearly stated in the revised methodology section.
- Furthermore, we have included a rationale for using SPSS, explaining its role in analyzing survey data and conducting descriptive and inferential statistical analyses, such as correlation and regression, to examine relationships between population dynamics and forest cover change. Supporting citations have been added where appropriate.

These revisions are included in the methodology section on pages 18 – 21.

**Comment # 5:**  Method of Data Analysis

• Strengths: The use of SPSS and Pearson correlation is appropriate for quantitative data.

• Weaknesses: The analysis lacks depth in exploring causality. For example, while correlation is established, causation is not discussed.

• Recommendation: Include regression analysis or other advanced statistical methods to explore causality. For qualitative data, provide representative quotes or narratives to substantiate thematic findings.

Response #5

We appreciate the reviewer’s thoughtful comments and suggestions to enhance the rigor of our data analysis. In response:

- We have extended our quantitative analysis by incorporating multiple linear regression models to better explore causal relationships between population growth variables and forest cover change. The regression results are now included and interpreted in the revised results section.
- In addition to Pearson correlation, the use of regression analysis enables us to assess the strength and direction of potential causal links while controlling for confounding variables.
- For the qualitative data, we have included representative quotes from key informant interviews and focus group discussions to support and illustrate the main themes identified. These narratives provide greater context and depth to the findings.

All additions and revisions are detailed in the results and conclusion sections on pages 21-22

**Comment #6. Results**

• Strengths: The results are comprehensive and supported by tables and figures.
• Weaknesses: The presentation is cluttered and lacks coherence. The socio-economic data is presented without linking it to the broader research question.

• Recommendation: Simplify tables and figures, ensuring each directly addresses a research objective. Use visual aids like trend lines or pie charts to make data more digestible.

**Response #6**

We thank the reviewer for acknowledging the comprehensiveness of our results and for the valuable suggestions to improve clarity and coherence. In response:

- We have simplified and reorganized the tables and figures to ensure each one directly corresponds to a specific research objective or key finding. Unnecessary or redundant visuals have been removed, and labels and captions have been improved for clarity.
- To enhance readability, we introduced more visual aids such as trend lines, pie charts, and bar graphs where appropriate—particularly for time-series and categorical socio-economic data. These revisions help highlight key trends and make the data more accessible to readers.
- Additionally, we revised the narrative surrounding the socio-economic data to better connect the findings with the main research question and theoretical framework. This strengthens the linkage between data and interpretation.

The revised figures and results are presented in the results section on pages Fig 1 on page 4, Figure 2 on page 10, figure 3 on page 15, and figure 4 on page 16, figure 5 on page 18, and Table 5 on page 21.

**Comment #7. Discussion**

• **Strengths:** The discussion connects findings to conservation and socio-economic issues.
• **Weaknesses:** There is insufficient engagement with contemporary literature. Key findings are not critically analyzed against previous studies.

• **Recommendation:** Incorporate a comparative analysis with global studies on forest degradation. Critically evaluate the implications of key results, such as the role of local governance in conservation efforts.

**Response # 7**

We thank the reviewer for this important suggestion to deepen the analytical strength of the discussion. In response:

- We have substantially revised the discussion section to include a broader engagement with recent and relevant global literature on forest degradation, land use change, and population dynamics. Comparative insights from studies in other tropical biosphere reserves and conservation areas (e.g., Southeast Asia, Central Africa, and Latin America) have been incorporated to contextualize our findings.
- We have critically examined how our results align with or diverge from previous studies, particularly regarding the drivers of forest loss and the socio-economic dimensions of resource pressure.
- Moreover, we have expanded our evaluation of the role of local governance in forest conservation. This includes a discussion of community-based forest management, institutional challenges, and the effectiveness of local bylaws and stakeholder engagement, supported by recent studies and best practices from similar settings.

These additions provide a more nuanced understanding of our findings and better position them within the global discourse on forest conservation. The revised discussion is found on pages 7- 20

**Comment # 8. Recommendations**

- **Strengths:** The recommendations are actionable and community-oriented.
- **Weaknesses:** They are broad and lack prioritization.
- **Recommendation:** Categorize recommendations into immediate, medium-term, and long-term actions. Justify recommendations with data and evidence from the study.

**Response # 8**

We thank the reviewer for the constructive feedback and for recognizing the community-oriented nature of our recommendations. In response:

- We have revised the recommendations section to categorize proposed actions into **immediate**, **medium-term**, and **long-term** interventions. This prioritization provides a clearer implementation framework for stakeholders.
- Each recommendation is now more explicitly supported by data and findings from our study—for example, deforestation hotspots identified through satellite analysis and community perceptions gathered through surveys and interviews.
- We also clarified the linkage between the proposed actions and the underlying causes of forest degradation identified in the research, ensuring that each recommendation is both evidence-based and aligned with the study objectives.

These improvements are presented in the revised conclusion and recommendations sections on pages 23, and are highlighted accordingly in the updated manuscript.

**Comment # 9:** Alignment of Research Problem, Objective, Methods, Results, and Discussion

- **Strengths:** The manuscript maintains alignment throughout its sections.
- **Weaknesses:** The linkage between objectives, findings, and recommendations is weak. For example, the methods section does not explain how the data supports specific recommendations.
- **Recommendation:** Create explicit connections between each section. For instance, demonstrate how the satellite imagery data directly informs the policy recommendations.

**Response # 9**

We thank the reviewer for highlighting the importance of internal consistency and coherence across the manuscript. In response:

- We have revised the **methods** and **discussion** sections to more clearly explain how each type of data collected—particularly the satellite imagery and socio-economic survey results—contributes directly to the study’s objectives and informs the recommendations.
- The **discussion** now includes explicit references to how land cover change data (e.g., forest loss from 1984 to 2024) supports targeted policy interventions such as zoning, reforestation efforts, and land use planning.
- We have also refined the **recommendations** section to include cross-references to the specific findings they are based on. This creates a stronger narrative thread from research problem to objective, methods, results, and actionable solutions.
- Where applicable, we included a summary table that links each research objective with the corresponding data, key findings, and resulting recommendations to make these connections more transparent to the reader.

These improvements appear on pages 18 to 21

**Comment # 10:** Suggestions for Authors

o Refine the introduction to frame the study within a global conservation context.
o Enhance methodological transparency by justifying data collection and analysis choices.
o Integrate more critical analysis in the discussion, contrasting findings with global studies.

**Response # 10**

We sincerely thank the reviewer for these valuable and constructive suggestions. In response:

- We have refined the **introduction** to more clearly position the study within a **global conservation context**. This includes discussion of the relevance of biosphere reserves to global biodiversity conservation, climate change mitigation, and sustainable development goals. We have also added citations to recent international studies and frameworks to support this global framing.
- To enhance **methodological transparency**, we have added justifications for our choices in data collection (e.g., selection of satellite imagery years, survey tools) and data analysis (e.g., use of SPSS, regression analysis). Supporting literature has been cited to provide methodological grounding and demonstrate adherence to established protocols.
- The **discussion** section has been revised to incorporate more **critical analysis**, with a comparative perspective that contrasts our findings with similar research conducted in other tropical and subtropical forest regions globally. This includes discussion of consistencies, divergences, and the broader implications of our results in terms of forest governance and conservation policy.

These revisions are reflected in the updated manuscript on pages 7 to 21, and the changes have been clearly marked for ease of review.

We would like to thank the editor and reviewers once again for their constructive comments and valuable suggestions. We believe the revisions have substantially improved the clarity, rigor, and relevance of our manuscript. All changes have been carefully addressed and are highlighted in the revised version. We hope the manuscript now meets the journal’s standards and expectations, and we respectfully submit it for your further consideration.

**Sincerely,**
Fikru Mosisa Hunde (Assistant professor)
On behalf of all authors
Department of Biology, College of Natural Sciences, Mattu University, Ethiopia

[fikrumosisa@gmail.com](mailto:fikrumosisa@gmail.com)
