# Supplementary material for: Impact of population pressure on forest resources depletion in Yayo coffee forest Biosphere Reserve, Southwest Ethiopia
Source: PLoS One. 2026 Jan 5;21(1):e0324407. doi: 10.1371/journal.pone.0324407 (PMC12768366; doi:10.1371/journal.pone.0324407)
Supplement: Attachment — Submitted filename: PLOS.docx [file pone.0324407.s007.docx]

**Title:**

**Impact of Population Pressure on Forest Resource Depletion in Yayo Coffee Forest Biosphere Reserve, Ilubabor Zone, Oromia Regional State, Southwest Ethiopia**

**General Comments:**

The manuscript presents a potentially important topic concerning the impact of population pressure on forest resource depletion within the Yayo Coffee Forest Biosphere Reserve. However, the overall quality of the paper does not meet the expected scientific and publication standards. The manuscript lacks organization, clarity, and scientific rigor in several sections. Below is a detailed evaluation of each component.

**Technical Issue:**

- The manuscript lacks **continuous line numbering**, making it difficult to provide specific comments or refer to precise sections of the text.

**2. Major Issues**

**2.1 Title**

- The title is **appropriate and well formulated**, clearly indicating the study area and the core issue of investigation.

**2.2 Abstract**

- The **abstract** is **weakly constructed and lacks structure**.
- The **data collection process** is **confusing and incomplete**. The author should specify how many respondents were included (key informants, kebele leaders, development agents, households), and clarify the roles of focus group discussions and field observations.
- The **study design and analytical methods** are not mentioned, leaving readers uncertain about how data were collected or analyzed.
- Inclusion of **livelihood activities** in the abstract is unnecessary and distracts from the research focus.

**Recommendation:**
The abstract should be revised to follow a clear and concise format:

1. **Background and objective** of the study
2. **Methods and data** (sample size, design, and analytical tools)
3. **Key findings** with quantitative evidence
4. **Conclusions and implications**

**2.3 Introduction**

- The **introduction** is **poorly developed** and **lacks articulation**.
- The study area is not properly **contextualized**, and the section fails to provide a **strong background or justification** for the research.
- The **problem statement** and **research gap** are missing or not explicitly defined.
- The **justification** for the study is weak, making it unclear why the research was necessary or how it contributes to existing literature.
- Only **four citations** are used throughout the section, which is insufficient to meet scientific and journal standards. The authors should consult recent and relevant studies to strengthen the background and demonstrate awareness of previous work.

**Recommendation:**
The introduction should:

1. Present the **broader context** of population growth and forest depletion in Ethiopia.
2. The specific issues in the Yayo Coffee Forest Biosphere Reserve.
3. Clearly **state the research problem** and **objectives**.
4. Identify the **research gap** and justify why this study is necessary.
5. Integrate at least **10–15 recent and relevant references** to strengthen the academic foundation.

**2.4 Materials and Methods:**

- This section is **not well organized** and **lacks coherence**.
- It includes **unnecessary sentences and paragraphs** rather than clearly outlining the data collection process, sampling procedures, data sources, and analytical techniques.
- There is no clear description of how data were obtained from different respondents or how the qualitative and quantitative information was analyzed.

**Recommendation:**
The section should be restructured to include:

1. **Description of the study area** (location, population, ecology).
2. **Sampling design and techniques** (how respondents were selected).
3. **Data sources** (primary and secondary).
4. **Data collection tools** (household surveys, key informant interviews, FGDs, observations).
5. **Data analysis** (statistical methods, qualitative coding, GIS/remote sensing tools, etc.).

**2.5 Results and Discussion**

- The results and discussion are **not scientifically presented** and lack analytical interpretation.
- The content reads more like a **descriptive report** than a scientific discussion, focusing excessively on general socio-economic characteristics and institutional narratives.
- The subsection **“Population Growth and Forest Cover Change”** is weakly supported; it does not establish any statistical relationship between population pressure and forest cover dynamics.
- The **“Satellite Image Results of Land Use Land Cover (1984–2007)”** subsection contains potentially valuable information but lacks analysis linking observed land cover changes to the stated hypothesis.

**Recommendation:**

- Present results using **quantitative evidence** (tables, figures, statistical tests).
- Discuss findings in comparison with previous studies to provide **scientific interpretation**.
- Use **appropriate statistical methods** (e.g., correlation, regression, trend analysis) to demonstrate relationships between population growth and forest cover change.

**2.6 Conclusion and Recommendations**

- The **conclusion** does not effectively summarize key findings or their implications.
- The **recommendations** are **generic** and not derived from the study results.

**Recommendation:**

- Summarize the **main findings** succinctly, focusing on their implications for forest management, policy, and sustainable livelihoods.
- Ensure that recommendations are **evidence-based**, **specific**, and **actionable**, directly linked to the findings presented.

**Overall Evaluation:**

- The manuscript does not meet the required publication standards in its current form.
- Therefore, it is regrettably rejected for publication
