# Supplementary material for: Impact of population pressure on forest resources depletion in Yayo coffee forest Biosphere Reserve, Southwest Ethiopia
Source: PLoS One. 2026 Jan 5;21(1):e0324407. doi: 10.1371/journal.pone.0324407 (PMC12768366; doi:10.1371/journal.pone.0324407)
Supplement: Attachment — Submitted filename: Response to Reviewers_2.docx [file pone.0324407.s008.docx]

**Response to Reviewer Comments**

**Manuscript Title**: Impact of Population Pressure on Forest Resources Depletion in Yayo Coffee Forest Biosphere Reserve, Ilubabor Zone, Oromia Regional State, Southwest Ethiopia
**Manuscript ID**: PONE-D-25-21231

Dear PLOS ONE Editorial Team,

We sincerely thank you for the opportunity to revise our manuscript and express our appreciation to the reviewers for their insightful comments and suggestions that have helped us to improve the quality and clarity of our paper.

We have carefully considered all the reviewer’s comments and revised the manuscript accordingly. Below we provide a detailed, point-by-point response to the reviewer’s remarks. For clarity, we have included each comment in bold followed by our responses in regular text. All changes made in the manuscript are highlighted for your convenience.

**Reviewer #1:**

**Comment #1.** Dear author’s thank you for your corrections of my previous comments and now the manuscript needs only minor revisions as I track in the pdf. Please correct them and your manuscript will be ready to publish!

**Response #1.** We sincerely thank the editor for the helpful comments and for recommending our manuscript for publication.

**Reviewer #3:**

**Comment #1:** -Streamline some long paragraphs in the discussion for conciseness and improve the narrative flow.

-Ensure all citations are formatted consistently according to PLOS ONE style.
-The conclusions could be shortened to focus on key findings and their policy implications rather than restating results.

-If possible, include a clearer policy framework diagram linking population dynamics, land use, and conservation responses

**Response #1.** Thank you for the valuable comments & the responses are as follows:

- In response, I have shortened and restructured the lengthy paragraphs in the discussion to make the section more concise. These revisions also help improve the narrative flow and ensure a clearer, more coherent presentation of the key points.
- I reviewed all references and have ensured that every citation is now formatted consistently and in full compliance with PLOS ONE style requirements.
- We have shortened the conclusions to emphasize the key findings and their policy implications, avoiding unnecessary restatement of results.
- We have added a clear policy framework diagram illustrating the links between population dynamics, land use, and conservation responses to improve conceptual clarity.

Reviewer #4:

**General Comment’s#**

The manuscript presents a potentially important topic concerning the impact of population pressure on forest resource depletion within the Yayo Coffee Forest Biosphere Reserve. However, the overall quality of the paper does not meet the expected scientific and publication standards. The manuscript lacks organization, clarity, and scientific rigor in several sections. Below is a detailed evaluation of each component.

**Response on General committee #**

Thank you for your general comments. We appreciate your recognition of this potentially important topic concerning the impact of population pressure on forest resource depletion within the Yayo Coffee Forest Biosphere Reserve.

Technical Issue:

Comment #: The manuscript lacks continuous line numbering, making it difficult to provide specific comments or refer to precise sections of the text.

Response #: We have implemented continuous line numbering throughout the entire manuscript to facilitate review and ensure clarity.

**Comments on major Issue # 2. Major Issues**

2.1 Title

•The title is appropriate and well formulated, clearly indicating the study area and the core issue of investigation.

**Response # 2.1 Title**

We thank you for your feedback on the title and appreciate your positive assessment of its clarity and relevance to the study.

**Comments on # 2.2 Abstract**

•The abstract is weakly constructed and lacks structure.

•The data collection process is confusing and incomplete. The author should specify how many respondents were included (key informants, kebele leaders, development agents, households), and clarify the roles of focus group discussions and field observations.

•The study design and analytical methods are not mentioned, leaving readers uncertain about how data were collected or analyzed.

•Inclusion of livelihood activities in the abstract is unnecessary and distracts from the research focus.

**Recommendation:**

The abstract should be revised to follow a clear and concise format:

1.Background and objective of the study

2.Methods and data (sample size, design, and analytical tools)

3.Key findings with quantitative evidence

4.Conclusions and implications

**Response on # 2.2 Abstract**

1. **Background and Objective of the Study:**

We have revised the background section to provide a clearer context for the study, highlighting the significance of population pressure on forest resource depletion. The objectives are now stated more explicitly to guide the reader through the research focus.

2. **Methods and Data (Sample Size, Design, and Analytical Tools):**

The methods section has been clarified, including details on the sample size, study design, and analytical tools used. We ensured that all methodological choices are transparent and reproducible.

3. **Key Findings with Quantitative Evidence:**

The results section now emphasizes the key findings supported by quantitative evidence, making the outcomes more precise and clearly linked to the research questions.

4. **Conclusions and Implications:**

The conclusions have been condensed to focus on the main findings and their policy and conservation implications, avoiding unnecessary repetition of results presented in the discussion.

**Comments on #2.3 Introduction**

•The introduction is poorly developed and lacks articulation.

•The study area is not properly contextualized, and the section fails to provide a strong background or justification for the research.

•The problem statement and research gap are missing or not explicitly defined.

•The justification for the study is weak, making it unclear why the research was necessary or how it contributes to existing literature.

•Only four citations are used throughout the section, which is insufficient to meet scientific and journal standards. The authors should consult recent and relevant studies to strengthen the background and demonstrate awareness of previous work.

**Recommendation:**

The introduction should:

1. Present the broader context of population growth and forest depletion in Ethiopia.

2. The specific issues in the Yayo Coffee Forest Biosphere Reserve.

3. Clearly state the research problem and objectives.

4. Identify the research gap and justify why this study is necessary.

5. Integrate at least 10–15 recent and relevant references to strengthen the academic foundation

**Responses on #2.3 Introduction**

1. **Broader Context of Population Growth and Forest Depletion in Ethiopia:**

We have expanded the introduction to provide a broader overview of how population growth in Ethiopia contributes to forest degradation, including national-level trends in forest cover change, demographic pressures, and impacts on natural resources.

2. **Specific Issues in the Yayo Coffee Forest Biosphere Reserve:**

We now highlight the particular challenges faced by the Yayo Coffee Forest Biosphere Reserve, such as the mix of land ownership regimes (public, private, and community), agricultural expansion pressures, and the critical role of forest coffee in local livelihoods.

3. **Research Problem and Objectives:**

The manuscript has been revised to clearly define the research problem: assessing the impact of population pressure on forest resource depletion in the Yayo Reserve under varying land-use regimes. The objectives are explicitly stated, including quantifying population effects on land-use change, evaluating conservation policy effectiveness, and proposing strategies for sustainable forest management.

4. **Research Gap and Justification:**

The introduction now identifies a clear research gap: although forest loss in Ethiopia has been studied, few studies integrate population dynamics, land-use regimes, and conservation policy within a biosphere reserve context. This study is necessary to provide actionable insights for targeted conservation and policy planning.

5. **Integration of Recent and Relevant References:**

We have incorporated 12–15 recent, peer-reviewed references (post‑2018) to strengthen the academic foundation, covering demographic trends, forest conservation strategies, and land-use modeling in tropical forest environments.

**Comments on # 2.4 Materials and Methods:**

•This section is not well organized and lacks coherence.

•It includes unnecessary sentences and paragraphs rather than clearly outlining the data collection process, sampling procedures, data sources, and analytical techniques.

•There is no clear description of how data were obtained from different respondents or how the qualitative and quantitative information was analyzed.

**Recommendation:**

The section should be restructured to include:

1.Description of the study area (location, population, ecology).

2.Sampling design and techniques (how respondents were selected).

3.Data sources (primary and secondary).

4.Data collection tools (household surveys, key informant interviews, FGDs, observations).

5.Data analysis (statistical methods, qualitative coding, GIS/remote sensing tools, etc.).

**Response on # 2.4 Materials and Methods:**

1. **Description of the Study Area (Location, Population, Ecology):**

We have enhanced the manuscript to provide a comprehensive description of the study area, including its geographical location, population characteristics, and ecological attributes. Details on land use, forest types, and the socio-economic context of communities within the Yayo Coffee Forest Biosphere Reserve have been added.

2. **Sampling Design and Techniques:**

The sampling design and selection criteria for respondents are now clearly outlined. We describe how households were selected, including stratification by land-use regime, to ensure representativeness and reliability of the data.

3. **Data Sources (Primary and Secondary):**

We clarify the data sources, specifying both primary (surveys, interviews, observations) and secondary (forest cover maps, demographic statistics, and policy documents) sources utilized in the study.

4. **Data Collection Tools:**

Details on data collection methods have been added, including household surveys, key informant interviews, focus group discussions (FGDs), and direct field observations. The rationale for employing each method is also explained.

5. **Data Analysis:**

The data analysis section has been expanded to describe the techniques used: quantitative data were analyzed using statistical methods, qualitative data were thematically coded, and GIS/remote sensing tools were applied to assess land-use changes and forest cover dynamics.

**Comment on # 2.5 Results and Discussion**

•The results and discussion are not scientifically presented and lack analytical interpretation.

•The content reads more like a descriptive report than a scientific discussion, focusing excessively on general socio-economic characteristics and institutional narratives.

•The subsection “Population Growth and Forest Cover Change” is weakly supported; it does not establish any statistical relationship between population pressure and forest cover dynamics.

•The “Satellite Image Results of Land Use Land Cover (1984–2007)” subsection contains potentially valuable information but lacks analysis linking observed land cover changes to the stated hypothesis.

**Recommendation:**

•Present results using quantitative evidence (tables, figures, statistical tests).

•Discuss findings in comparison with previous studies to provide scientific interpretation.

•Use appropriate statistical methods (e.g., correlation, regression, trend analysis) to demonstrate relationships between population growth and forest cover change.

**Response on # 2.5 Results and Discussion**

1. **Presentation of Results Using Quantitative Evidence:**

We have revised the results section to include quantitative evidence, presenting key findings through tables, figures, and relevant statistical summaries to clearly illustrate the observed patterns.

2. **Discussion in Comparison with Previous Studies:**

The discussion has been strengthened by comparing our findings with previous studies, providing scientific interpretation and context. We highlight consistencies and differences with earlier research and discuss possible explanations for observed trends.

3. **Use of Appropriate Statistical Methods:**

We have applied suitable statistical analyses, including correlation, regression, and trend analysis, to examine and demonstrate the relationships between population growth and forest cover change. The results of these analyses are now clearly presented and interpreted in the context of the study objectives.

**Comments on # 2.6 Conclusion and Recommendations**

•The conclusion does not effectively summarize key findings or their implications.

•The recommendations are generic and not derived from the study results.

**Recommendation:**

•Summarize the main findings succinctly, focusing on their implications for forest management, policy, and sustainable livelihoods.

•Ensure that recommendations are evidence-based, specific, and actionable, directly linked to the findings presented.

**Response on # 2.6 Conclusion and Recommendations**

Summary of Main Findings:

We have revised the conclusions to succinctly summarize the main findings, emphasizing their implications for forest management, policy development, and the promotion of sustainable livelihoods within the Yayo Coffee Forest Biosphere Reserve.

We would like to thank the editor and reviewers once again for their constructive comments and valuable suggestions. We believe the revisions have substantially improved the clarity, rigor, and relevance of our manuscript. All changes have been carefully addressed and are highlighted in the revised version. We hope the manuscript now meets the journal’s standards and expectations, and we respectfully submit it for your further consideration.

**Sincerely,**
Fikru Mosisa Hunde (Assistant professor)
On behalf of all authors
Department of Biology, College of Natural Sciences, Mattu University, Ethiopia

[fikrumosisa@gmail.com](mailto:fikrumosisa@gmail.com)
